# Modeling wood product carbon flows in southern us pine plantations: implications for carbon storage

**DOI:** 10.1186/s13021-024-00254-4

**Published:** 2024-02-21

**Authors:** Sarah J. Puls, Rachel L. Cook, Justin S. Baker, James L. Rakestraw, Andrew Trlica

**Affiliations:** 1 Department Forestry and Environmental Resources, North Carolina State University, Raleigh, NC 27695 USA; 2 International Paper, Statesboro, GA 30458 USA

**Keywords:** Harvested wood products, Forest carbon modeling, Loblolly, Life cycle assessment

## Abstract

**Background:**

Wood products continue to store carbon sequestered in forests after harvest and therefore play an important role in the total carbon storage associated with the forest sector. Trade-offs between carbon sequestration/storage in wood product pools and managed forest systems exist, and in order for forest sector carbon modeling to be meaningful, it must link wood product carbon with the specific forest system from which the products originate and have the ability to incorporate in situ and ex situ carbon synchronously over time.

**Results:**

This study uses elements of a life cycle assessment approach, tracing carbon from US southern pine timber harvests to emission, to create a decision support tool that practitioners can use to inform policy design around land- and bioproduct-based mitigation strategies. We estimate that wood products from annual loblolly and shortleaf pine timber harvests across the southern US store 29.7 MtC in the year they enter the market, and 11.4 MtC remain stored after 120 years. We estimate fossil fuel emissions from the procurement, transportation, and manufacturing of these wood products to be 43.3 MtCO_2_e year^−1^. We found that composite logs, used to manufacture oriented strand board (OSB), were the most efficient log type for storing carbon, storing around 1.8 times as much carbon as saw logs per tonne of log over 120 years.

**Conclusions:**

Results from our analysis suggest that adjusting rotation length based on individual site productivity, reducing methane emissions from landfills, and extending the storage of carbon in key products, such as corrugated boxes, through longer lifespans, higher recycling rates, and less landfill decomposition could result in significant carbon gains. Our results also highlight the benefits of high site productivity to store more carbon in both in situ and ex situ pools and suggest that shorter rotations could be used to optimize carbon storage on sites when productivity is high.

**Supplementary Information:**

The online version contains supplementary material available at 10.1186/s13021-024-00254-4.

## Introduction

According to the 2022 IPCC Climate Change Mitigation Report, net CO_2_ emissions from 2010 to 2019 were around 33% of our remaining carbon budget from 2020 onwards for a 67% probability of limiting global warming to the 2 °C limit [1]. Effective climate change mitigation requires efforts across many sectors to both reduce emissions of greenhouse gases (GHG) and to actively remove them from the atmosphere [1]. The global forest sector is in a unique position to contribute to these mitigation goals. Harris et al. [2] estimate that, globally, forests sequester around 7.6 GtCO_2_e year^−1^, and recent estimates [3] indicate that the global forest sector could support an additional 1.2–5.8 GtCO_2_e year^−1^ in carbon sequestration under climate policy incentives. Studies have highlighted the potential role of wood-based bioenergy [4, 5] and wood product substitution for more energy-intensive materials [6] as important mitigation strategies. Other studies point to forest carbon gains from deferred harvests, reforestation, or risk mitigation efforts [7–9]. Forests also provide wood products, which continue to store an estimated 335 MtCO_2_e year^−1^ globally after harvest [10], impacting the total carbon storage capacity of forest stands over time. However, despite the significant impact of wood product carbon storage on total forest carbon, wood products are often excluded from modeling efforts to evaluate possible forest sector mitigation strategies.

Studies that do include wood product carbon vary considerably in their findings. One recent study suggests that harvested wood products (HWPs) could generate a substantial source of emissions globally over the next decade using a simulation approach that ignores systematic changes in forest productivity and management responses to growing wood demand [11]. Other recent studies using economic models that capture interactions between forest product markets and management interventions suggest that wood product and terrestrial forest carbon pools can grow jointly at global and regional scales under certain market and policy conditions [3, 12, 13]. Integrated assessment and forest sector models that more adequately capture the complexities of carbon flows in processing and final wood product streams are required to inform management and policy changes and optimize wood product and forest carbon storage for climate mitigation.

Further, while there is a large and growing literature that has applied attributional life cycle analysis techniques to simulate wood product carbon flows, these analyses often do not capture interactions between HWP carbon flows and forest management regimes, which can affect both carbon sequestration and wood product disposition. More detailed accounting of wood product carbon flows is particularly important for high productivity commercial species where adjustments to silvicultural management regimes (e.g., rotation length) across many stands could have significant effects on forest sector carbon storage.

Southern pine plantations offer a unique opportunity to analyze HWP carbon through this approach in a way that contributes significantly to climate change mitigation strategy for the US. Composed primarily of loblolly pine (*Pinus taeda* L.), southern pine plantations account for 22% of forest area, 71% of all planted timberland, and 51% of forest growth in the southern US [14]. Collectively, the southern US produces around 60% of US timber products and more timber than any individual country in the world [15].

The goal of this study is to provide a current comprehensive analysis of the wood product carbon flow associated with southern pine grown in the southern US and identify several potential areas of improvement for climate benefits. Our objectives are to (1) create a novel modeling framework that can project ex situ carbon flows across different wood product pools and over time and is adaptable to different species and regions, (2) develop parameters to adapt this framework to southern pine grown in the southern US, (3) evaluate the sensitivity of loblolly and shortleaf pine ex situ carbon flows to key model parameters (e.g., lifespan, milling efficiency, and recycling rates), (4) link the developed framework with a growth and yield model to assess total (i.e., in situ and ex situ) carbon storage from two common silvicultural management regimes for loblolly pine plantations (20-year pulpwood regime, and 30-year sawtimber regime), and (5) compare the estimated business as usual carbon flows from loblolly and shortleaf pine wood products to hypothetical wood usage scenarios involving changes in wood consumption and technology parameters.

### Wood product carbon modeling background

Wood product carbon models are used to estimate and project stored carbon over time for various purposes. HWP carbon was accepted as part of a country’s GHG reduction contributions at the 17th Conference of the Parties (COP17) and must be reported by all Annex I Parties. The US Environmental Protection Agency (EPA) adopted the methodology developed by Skog [16] for harvested wood product stock estimates in national greenhouse gas inventories [17]. HWP carbon is sometimes included in carbon offset projects as an additional benefit from changes in forest management associated with the project [18, 19]. HWP carbon may also be used in decision making for sustainable forest management, studies analyzing the effectiveness of climate change mitigation strategies, carbon inset accounting (i.e., reducing GHG emissions or increasing removals within an actor’s own supply chain, such that the actor’s net emissions are reduced), and the design and evaluation of climate smart commodities programs [20–26].

Typical wood product carbon models use some input of harvested timber, whether from a growth and yield model or actual harvest data, and sort the timber by product class [27–31] and either species type (i.e., softwoods or hardwoods) [28, 30, 31] or forest type [32, 33]. These models typically simulate a milling process through mill efficiency factors that may be specific to product class, species type, and/or region. Mill residues, such as wood chips and bark, are often considered to be immediately emitted [28, 31, 34], though some models attempt to capture a simplified version of residue carbon flow [35]. Fossil fuels burned in the procurement, transportation, or milling process are sometimes accounted for in these models [34, 35]. A portion of stored carbon is sometimes deducted between the milling process and the products’ useful life to account for construction or manufacturing waste [31]. After the simulated milling process, products enter use and are typically transitioned out of use according to a first-order decay formula with half-lives for each category of primary products (lumber, plywood, paper, etc.) [32, 36]. If the recycling of products such as paper is included, it is typically built into the half-life parameters [32]. After their useful life, products are discarded into landfills or burned. Models often categorize carbon at reported years as being either still in use, emitted with energy capture, emitted without energy capture, or in a landfill [28, 30–32, 34, 37]. After being landfilled, some portion of product carbon typically continues to decay according to solid wood-specific and paper-specific half-lives [31, 34, 38, 39].

Though these models vary in complexity and use a wide range of parameters, their boundaries are similar, beginning with some regional or national timber input and reporting carbon stock estimates derived from that timber. While this approach is sufficient for stock estimates, when analyzing the carbon potential of alternative mitigation strategies, this approach considers only one component of a larger system [40]. A wood product carbon model parameterized for a specific species and region that can be linked with a forest carbon model for the same species and region has the potential to analyze trade-offs between carbon sequestration/storage in wood product pools and forest systems. For example, there are trade-offs involved in the timeline of both the forest systems that grow the wood and the use and disposal of the wood products. Many products that remain in use for a shorter period of time are grown in forest systems with shorter rotations, and vice versa. Therefore, though the longer-lived products will store more carbon in the HWP pool over time, the shorter-lived products are concurrent with younger forest systems that sequester carbon at a faster rate. Rotation lengths also vary greatly among species and forest ownership types, suggesting that the most effective mitigation strategy within the context of one species or ownership type may not be the most effective in another.

Significant losses also occur when carbon is transferred from in situ carbon, or carbon in the forest, to ex situ carbon, or carbon in wood products. The US Forest Service estimated harvest residue (i.e., growing and non-growing stock) from softwood removals in the southern US to be around 16% of the aboveground tree carbon [41]. Estimates for belowground tree carbon are rarely included post-harvest though significant amounts can remain for decades [42]. More carbon is emitted during the milling process as mill residue is used to fuel production [43]. Additionally, forest managers, especially in the context of plantations, have considerable control over both the carbon sequestration rates of a forest stand and the types of wood products that will be produced when the stand is harvested. Silvicultural management tools such as rotation length, planting density, fertilization, herbicide application, genetic material, and thinning are all used to manipulate forest sequestration rates (i.e., growth rates) and timber product classes at harvest. Therefore, in order for forest sector carbon modeling to be meaningful, it must link wood product carbon with the specific forest system (e.g., species, region, management, etc.) from which the products originate and have the ability to incorporate in situ and ex situ carbon synchronously over time.

## Model documentation and methods

### LobWISE model description

**Fig. 1:**
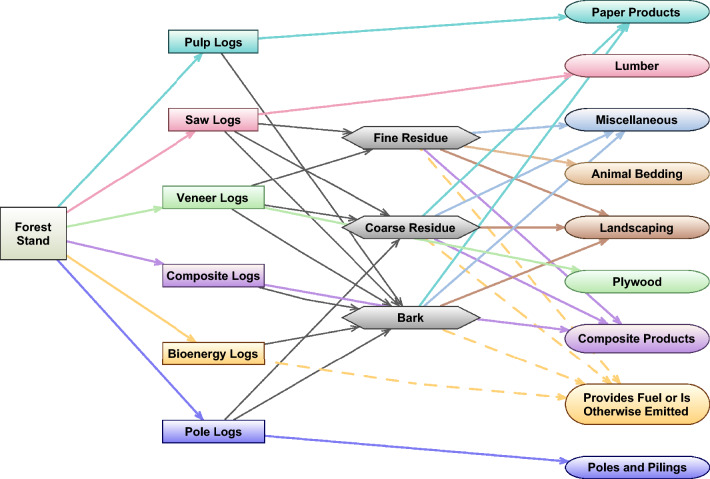
Flow of carbon from harvest, through the milling process, and into primary products

This study uses elements of a life cycle assessment (LCA) approach to create a framework, based in Microsoft Excel, that traces wood product carbon through five processes: (1) harvest, (2) milling (Fig. 1), (3) post-mill manufacturing, construction, and use, (4) recycling, downcycling, and landfilling, and (5) emission (Additional file 1: Figure S1). The framework takes inputs of four different log size classes or eight different log types, in either green weight or volume, and returns a number of outputs, including carbon remaining stored in various uses and in landfills at any given point in time, biogenic CO_2_ and methane emissions, and fossil fuel emissions associated with the production of the HWPs. The parameters for the framework can be developed for any species or region if data are available and updated as consumption patterns and technology change and new studies shed light on uncertainties.

#### System boundaries

For this study, we construct a model, Loblolly Wood Inventory, Storage, and Emissions (LobWISE), by developing parameter values (Additional file 1: Tables S1–25) for the proposed framework that are specific, where data exist, to loblolly pine grown in the southern US. The following model description is specific to LobWISE, which was used to conduct the analyses in this study, but the concepts and formulas described are those used in the adaptable framework as well.

Where data exist, LobWISE is specific to carbon that originated from loblolly pine grown in southern US forests, regardless of where the products are consumed. The southern US is defined as including Alabama, Arkansas, Florida, Georgia, Kentucky, Louisiana, Mississippi, North Carolina, Oklahoma, South Carolina, Tennessee, Texas, and Virginia. It assumes that exported products are consumed similarly to domestic products and that there is a national market for wood products. Since most wood product data only distinguish between softwoods and hardwoods, rather than between species, softwood was frequently used to represent loblolly wood. Four species of pine in the southern US are typically referred to as southern pine: loblolly pine (*Pinus taeda* L.), longleaf pine (*Pinus palustris* Mill.), shortleaf pine (*Pinus echinata* Mill.), and slash pine (*Pinus elliottii* Engelm.). The wood from these four species is similar and is used for the same products [44]. Therefore, though this study focuses on loblolly pine because of its prevalence in southern pine plantations, it is appropriate to derive wood product carbon outputs from LobWISE for any of the southern pine species. The framework produces 120 years of outputs after the initial harvest to be consistent with the common 100-year definition of permanence in carbon offset accounting and national stock estimations and to allow for the comparison of 20- vs. 30-year loblolly pine rotations that was conducted in this study. Year 0 is considered to be the time at which the wood products enter the market, thus we do not account for cumulative storage or emissions from an existing wood product stock.

#### Harvest

Inputs for LobWISE are in the form of whole logs that are harvested from a timber stand by either log type or diameter class (i.e., 13–22 cm, 23–32 cm, 33–47 cm, and 48+ cm), depending on the data available. When size class inputs are used, LobWISE uses regional cull factors (Additional file 1: Table S17) developed from US Forest Service Timber Products Output (TPO) data to sort the logs into various types [45]. In this study, the silvicultural management comparison used diameter-based inputs. All other inputs were by log type. Log types include pulp logs, chip-n-saw (CNS) logs, saw logs, large saw logs, veneer logs, pole logs, composite logs, and bioenergy logs.

#### Milling

In the milling process, whole logs undergo a number of transformations to produce primary products. First, inputs are converted into tC. Next, mill residue, including bark, coarse residue (chips, slabs, edgings, trims, cores, etc.), and fine residue (sawdust and shavings) is removed from the log, leaving primary products, according to mill efficiencies for each log type (Additional file 1: Table S18). Residue is then used to manufacture wood pellets, otherwise burned or emitted, used for landscaping or animal bedding, used for fiber production (pulp and paper products or non-structural panels), or used for a miscellaneous category. Resulting primary products include softwood lumber, softwood plywood, oriented strand board (OSB), non-structural panels, engineered wood, poles, pulp and paper products, landscaping, animal bedding, and miscellaneous. The breakdown of specific primary products is thus based on the timber product inputs, mill efficiencies, and residue uses. Regional averages are used to determine residue production and use (Additional file 1: Tables S19, 20). Fossil fuel emissions associated with the procurement of logs, including planting, silviculture, and harvest operations, transportation of logs from the harvest site to the mill, and production of primary products from logs at the mill are also calculated based on the type and amount of primary product produced. These are reported in tCO_2_e and are not distinguished between different greenhouse gases. The fossil fuel emission factors used are based on LCA studies of each product and are based on typical activities for the southern US, according to the respective study authors (e.g., typical silviculture, average distance to mill, etc.) (Additional file 1: Table S12). Fossil fuel emissions for post-mill activities (e.g., recycling, construction, post-mill transportation, etc.) are not included.

#### Manufacturing, construction, and use

Primary products are sorted into secondary products based on national consumption averages. Secondary products include new single family homes, new multi-family homes, new manufactured homes, single family home upkeep, multi-family home upkeep, manufactured home upkeep, new buildings, other new structures, other construction (i.e., building upkeep, etc.), shipping pallets, other shipping, utility poles, posts and pilings, animal bedding, landscaping, corrugated boxes, sanitary products, packaging cartonboard, disposable food related products, miscellaneous paper products, furniture, other manufacturing, and miscellaneous (see Additional file 1 for descriptions). A portion of secondary product carbon for certain products is transitioned out of use in year 0, according to waste deduction parameters. The construction waste deduction (5.6%) applies to new residential construction, residential repair, new non-residential construction, and other construction [32, 46, 47]. The manufacturing waste deduction (8%) applies to shipping pallets, other shipping, posts and pilings, furniture, other manufacturing, and miscellaneous [31]. After year 0, the remaining secondary product carbon transitions out of use according to Weibull distribution functions (Eq. 1). The functions use 2.63 as a shape factor (*k*) and estimated modes unique to each product according to the literature to calculate the scale factor (λ) (Eq. 2) [48].

1 $$f\left( x \right) = C - C\left( {1 - e^{{ - \left( {x/\lambda } \right)^{k} }} } \right)$$

2 $$\lambda = \frac{mode}{{\left( {1 - \frac{1}{k}} \right)^{\frac{1}{k}} }}$$

where *C* is the carbon in products that enter the market, *x* is the time since harvest in years, *k* is the shape factor, λ is the scale factor, *mode* is an approximation of the product’s lifespan, and *f(x)* is the carbon remaining in use at year *x*. Typically, transition distributions in ex situ carbon models are based on a first order decay function. However, there is no consensus suggesting that this distribution is best suited to model product transitions out of use, and other models, including Weibull, lognormal, gamma, and Gompertz have been suggested as possible alternatives [49, 50]. A well-suited distribution should result in slow transition during the earlier years, the majority of the transition close to some estimated lifespan for the product, and transition in the later years that resembles a first order decay curve [50].

Carbon that is sorted into new single and multi-family homes transitions according to specific applications within the home (e.g., floors, walls, doors, etc.) and type of primary product, rather than the home as a whole. All other secondary products transition according to the lifespan of the secondary product, regardless of its different pieces or product makeup.

#### Recycling, downcycling, and landfilling

When carbon transitions out of its first use, it is either recycled, downcycled, landfilled, or emitted. Shipping pallets and all paper products can be recycled within the framework, though the default values within LobWISE assume no recycling of sanitary products or disposable food related products [51]. An individual paper product can only be recycled as the same product. For example, packaging cartonboard can be recycled as packaging cartonboard but not as corrugated boxes. This structure is not realistic, as some types of paper are more likely to be made from recycled material than others, but building a network of recycling exchanges would be very challenging given the complexity of the paper recycling product flow and lack of data. Furthermore, landfilling, recycling, and incineration rates are typically available only on the basis of all waste entering the waste stream and do not differentiate newer products from products that can no longer be recycled [51]. Within this framework, these generalized rates potentially overestimate emissions from wood products in the first few years and underestimate storage and emissions from landfills in later years. Pallets and paper products are recycled a limited number of times. Carbon that transitioned out of use due to construction and manufacturing waste are not recycled. The framework is structured such that all products can be downcycled as landscaping, animal bedding, posts and pilings, furniture, and other manufacturing, according to national averages, though default values within LobWISE include downcycling only for construction products, shipping products, and utility poles. Products are landfilled according to national averages in either municipal solid waste (MSW) landfills or construction and demolition (C&D) landfills. A portion of carbon in landfills decays according to a first order decay function with half-lives for a solid wood product category and a paper product category (Eq. 3).

3 $$f\left( x \right) = \mathop \sum \limits_{i} \left( {C_{i} \times DOC_{f} \times e^{{\left( { - x\left( {\frac{\ln \left( 2 \right)}{k}} \right)} \right)}} + C_{i} \times \left( {1 - DOC_{f} } \right)} \right)$$

where *C*_*i*_ is the carbon that enters a landfill in year *i*, *DOC*_*f*_ is the fraction of carbon in the product that can decay over time, *x* is the time since the product was landfilled in years, *k* is the half-life of the product in a landfill, and *f(x)* is the carbon remaining stored in a landfill at time *x*.

#### Emission

As products transition out of use, product carbon that is not recycled, downcycled, or landfilled is assumed to be emitted as biogenic CO_2_. Product carbon is also emitted as biogenic CO_2_ as landfilled products decay. Methane produced by decaying products in landfills that is not captured or oxidized is emitted as biogenic methane. CO_2_ and methane emissions are reported in tCO_2_e. No distinction is made between biogenic carbon emitted with and without energy capture (i.e., only scope 1 emissions). Lag time, sometimes used in landfill modeling to account for the time between product disposal and the time at which conditions are suitable for methane production [52], is assumed to be 0 years [38].

### Sensitivity analysis

LobWISE incorporates over 600 parameters, ranging in certainty from fairly high to very low. We conducted a sensitivity analysis to examine which parameters have the greatest leverage on model outputs to provide insight into the real-world factors that have the most effect on the climate impact of wood products. The sensitivity analysis can also help to identify those factors with low certainty and high influence, about which further scientific research would provide the most benefit. Parameters that were expected to have meaningful leverage on model outputs were tested, with the exception of parameters related to market conditions and consumer patterns (i.e., log type mix and end use category for primary products), as these tests are better suited for an economic analysis. The sensitivity analysis method used is the Morris method [53], a method of global sensitivity analysis that uses a design of experiments approach to calculate the elementary effect of each input parameter on the model output. This method is computationally frugal, making it suitable for models with slow run time or many parameters, and is appropriate for Excel-based life cycle models [54]. In this method, sample parameter sets are randomly generated based on value bounds specific to each parameter. The results indices are µ, µ*, and σ. The µ index represents the mean elementary effect of an individual input parameter and indicates the overall influence of the parameter on the model output. The µ* index represents the mean of the absolute values of the elementary effects and indicates both the direct effects of an input parameter and the effects of interactions with other input parameters on the model output. When µ ~ µ*, the parameter has a linear positive effect on the model output. When µ ~ − µ*, the parameter has a linear negative effect on the model output. The σ index represents the standard deviation of the elementary effects and indicates the level of interaction between the parameter and other parameters. When |µ|≠ µ* or σ is not negligible compared to µ*, then either the effect of the parameter is nonlinear or there are interactions between the parameter and other parameters [55, 56]. A list of all parameters tested and their value bounds is provided in the Additional file 1: Table S36. For most parameters, value bounds were ± 10% of the default value for parameters with high certainty and ± 20% of the default value for parameters with low and moderate certainty. High certainty is defined as having similar values for the specific parameter that were empirically derived in one or more studies or from published data. Moderate certainty is defined as having similar numbers for a related or generalized parameter that were empirically derived from one or more studies or from published data, or the specific parameter was cited in multiple studies but was not empirically derived. Low certainty is defined as having a related or generalized parameter that was cited in one or more studies but not empirically derived, the specific parameter was cited in only one study and not empirically derived, or information on the parameter is unavailable and based on the professional judgement of the authors. Some parameters had different adjustments based on unique situations (e.g., to capture the range of estimates for the lifespan of paper products present in the literature, the lifespan of paper products was decreased by 50% and increased by 200%). The programming software, Python 3.11.1, was used to generate the sample sets, interact with LobWISE to run each sample set and collect outputs, and analyze the outputs. Each parameter was discretized into 10 levels, and the number of approaches for each parameter was set to 30 [54].

**Fig. 2:**
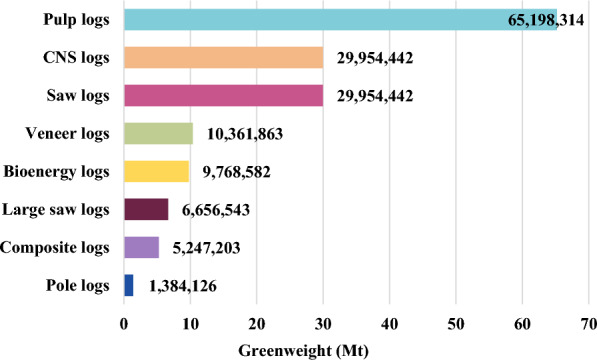
Southwide loblolly and shortleaf pine timber production from 2020. Adapted from [45]

For each sample set, emission pulses from each year, including both biogenic and fossil fuel emissions, were discounted back to year 0 at a 3% rate [57], such that a change in carbon at year 100 was less significant than an equivalent change at year 10 (Eq. 4).

4 $$GHG_{PV} = \frac{{GHG_{FV} }}{{\left( {1 + r} \right)^{x} }}$$

where *GHG*_*FV*_ is the future value of the GHG pulse emission in year *x*, *r* is the discount rate, *x* is the time since harvest in years, and *GHG*_*PV*_ is the present value of the GHG pulse emission in year *x*. Discount rates represent the heightened value that society places on emissions avoided today rather than tomorrow because of the compounding damage that results the longer atmospheric GHG levels are not reduced, the current cost of GHG emissions to society (i.e., social cost of carbon), and the potential for technology and policy improvements that lower GHG emissions to be implemented in the future. This methodology also reduces the effect of an arbitrary end year on the results of carbon impact comparisons [23, 58, 59]. It is particularly important to consider the time value of carbon within the context of forest systems because of the temporal trade-offs associated with changing annual growth rates and the longevity of HWP carbon. The sum of all discounted emission pulses was used as the model output for the sensitivity analysis. GHG emissions, rather than carbon storage, were used as the collected output because the interest of the analysis was climate impact, and, ultimately, it is the radiative forcing effect of increasing atmospheric levels of GHGs that cause atmospheric warming. To fully capture the temporal effects of emission pulses, dynamic carbon accounting should be used to decay pulse emissions over time and observe the cumulative radiative forcing effects of each GHG [60]. However, dynamic accounting is beyond the scope of this study, and we believe emission pulse sums to be a sufficient metric for this analysis. The inputs used for the analysis were loblolly and shortleaf pine timber production across the southern US from 2020, as reported in TPO data (Fig. 2) [45]. Shortleaf pine was included because loblolly and shortleaf pine are reported as a single species category in TPO harvest data. Saw logs were assumed to be 45% CNS logs, 45% saw logs, and 10% large saw logs [61].

### Silvicultural management scenarios

Four silvicultural management scenarios were analyzed by modeling both in situ and ex situ carbon storage over 120 years. In situ carbon and harvested green weight were modeled with the NC State University-Virginia Tech Forest Productivity Cooperative’s Loblolly Decision Support System (LobDSS v.3.1.0.1), which is the simulator wrapped around the FASTLOB 3.1 growth and yield model [62, 63]. LobDSS was chosen because it is specific to loblolly pine, and more accurately models green weight than publicly available models, such as PMRC 1996 and PTAEDA [64]. The scenarios were designed to represent typical regimes for loblolly pine plantations and included: (1) a low productivity pulp regime, (2) a low productivity sawtimber regime, (3) a high productivity pulp regime, and (4) a high productivity sawtimber regime (Table 1). Shorter rotations *(*i.e., ~ 20–25 years) are typically used to produce lower value timber products, such as pulpwood, and longer rotations (i.e., ~ 25–30 years) are typically used to produce higher value timber products, such as sawtimber. Therefore, we denote the pulp regime by a 20-year rotation and the sawtimber regime by a 30-year rotation [39]. Both scenarios for the sawtimber regime included a thin at age 15 down to 16.1 m^2^ of basal area hectare^−1^. The initial planting density used for all scenarios was 1236 trees hectare^−1^. Productivity was modeled by changing site index, which is a measure of the height of dominant and co-dominant trees at a given base age (i.e., 25 years). No fertilizer or herbicide applications were simulated. The in situ carbon pools modeled included crop tree stems, branches, foliage, coarse roots, fine roots, and coarse woody debris. Soil and forest floor carbon was assumed to be constant between the management scenarios [65–67]. Carbon in non-crop tree vegetation is outside the scope of LobDSS, though this pool may be somewhat constant between management scenarios since herbicide use was not modeled. All root, harvest residue, and coarse woody debris carbon was considered to be emitted in the year of final harvest. Because the outputs generated by LobDSS are in terms of tons of logs per size class, regional cull factors for each size class were used to convert size class tonnage to log type tonnage (Additional file 1: Table S17). To account for the varying levels of fossil fuel emissions from procurement, transportation, and production that occur from different log type mixes, the amount of GHG remaining in the atmosphere after the initial pulse emission in the harvest year was calculated according to the revised version of the Bern carbon cycle model, as reported in the 5th Assessment Report of the IPCC (Eq. 5) [68].

5 $$C_{{CO_{2} }} \left( t \right) = a_{0} + \mathop \sum \limits_{k} a_{k} e^{{ - t/\tau_{k} }}$$

*a*_0_ = 0.2173; *a*_1_ = 0.224; *a*_2_ = 0.2824; *a*_3_ = 0.2763; τ_1_ = 394.4 years; τ_2_ = 36.54 years; τ_3_ = 4.304 years.

**Table 1:** Silvicultural management scenarios

Scenario	Site index (SI) (m)	Rotation length (years)	Thinning
S1. Low productivity pulp (Pulp-20)	20	20	No thin
S2. Low productivity sawtimber (ST-20)	20	30	Thin, age 15
S3. High productivity pulp (Pulp-24)	24	20	No thin
S4. High productivity sawtimber (ST-24)	24	30	Thin, age 15

High and low productivity is measured by site index at base age 25; pulp and sawtimber regimes are noted by rotation length

where C_CO2_(*t*) is the amount of CO_2_ that remains in the atmosphere, *t* years after harvest. The tC equivalent to this amount was then subtracted from the ex situ storage for each year to deduct the fossil fuel emissions from the overall storage produced in the silvicultural management scenario. Sums of annual storage over 120 years, discounted at 3%, were used to compare scenarios. Undiscounted annual storage values for years 60 and 120 are also provided in Additional file 1: Tables S37–38.

### Wood usage scenarios

Three wood usage scenarios, intended to represent possible real-world shifts in consumer preferences, policy changes, and technological developments, were tested by modifying specific parameters (Table 2). The first scenario represents a path in which sawmills prefer smaller diameter logs over larger logs. The breakdown of saw logs was changed from 45% CNS logs, 45% saw logs, and 10% large saw logs to 80% CNS logs, 20% saw logs, and no large saw logs, creating around 7% less lumber and 4.5% more of each saw residue. The lifespan of furniture was decreased to account for more furniture made with non-structural panels. Fossil fuel rates were also increased because rates are based on tonnage of product produced, and it was assumed that smaller logs consumed similar rates of fossil fuels. The second scenario represents a more waste-conscious society, in which products are used for longer and recycled at higher rates and more times. The third scenario represents a policy shift towards wood energy production. The tonnage of non-growing stock timber (tops, limbs, stumps, and cull sections) used for bioenergy was increased by 100%, and the tonnage of growing stock timber used for bioenergy was increased by 600%, with 90% coming from pulp log volume and 10% from saw log volume. These represent the highest increases possible (to the nearest 100%) without increasing total harvest volume or decreasing total log volume (non-growing stock + growing stock) by more than 40% (for pulp logs) and 5% (for saw logs). For simplicity, all tested harvest combinations had the same total tonnage. Sums of annual storage and emission pulses over 120 years, discounted at 3%, were used to compare scenarios. Undiscounted annual storage and emission pulse values are also provided in Additional file 1: Tables S41–48.

**Table 2:** Parameters were adjusted to test the relative carbon benefits of three potential wood usage scenarios

Scenario	Parameter name	Adjusted value of parameter	% change
S1. Timber product transition: smaller logs preferred by sawmills (> CNS)	CNS produced	53,252,326 t	+ 78%
	Sawtimber produced	13,313,081 t	− 56%
	Large sawtimber produced	0 t	− 100%
	Furniture lifespan	10 years	− 23%
	Fossil fuel consumption per ton of lumber carbon produced		
	Procurement	0.01986454	+ 7%
	Transportation	0.01793715	+ 7%
	Production	0.09434880	+ 7%
S2. Products are used for longer periods of time and recycled at higher rates (> Lifespan)	All product lifespans (except sanitary products, disposable food related products, packaging cartonboard, animal bedding, and landscaping)	–	+ 10%
	Pallet recycling		
	Recycling rate	0.8	+ 10%
	Number of times recycled	5	+ 2 times
	Paper products recycling		
	Corrugated boxes	0.964	+ 5%
	Sanitary products	0	–
	Packaging cartonboard	0.608	+ 40%
	Disposable food related products	0.1	+ 10%
	Miscellaneous paper products	0.181	+ 10%
	Number of times recycled	8	+ 3 times
	Paper products landfilling		
	Corrugated boxes	0.028	–
	Sanitary products	0.656	–
	Packaging cartonboard	0.24	− 20%
	Disposable food related products	0.719	− 10%
	Miscellaneous paper products	0.478	− 10%
	C&D waste		
	Downcycling to non-structural panels	0.129	+ 10%
	Landfilling	0.622	− 10%
S3. Use of potential pulpwood for bioenergy production (> Bioenergy)	Pulp logs produced	39,724,441 t	− 39%
	CNS logs produced	28,680,741 t	− 4%
	Sawtimber logs produced	28,680,741 t	− 4%
	Large sawtimber logs produced	6,373,498 t	− 4%
	Veneer logs produced	10,361,863 t	–
	Pole logs produced	1,384,126 t	–
	Composite logs produced	5,247,203 t	–
	Bioenergy logs produced	38,072,862 t	+ 290%

## Results

### LobWISE results for the southern US

#### Estimated HWP storage and emissions across the southern US from loblolly and shortleaf pine timber, based on TPO harvest data for 2020

**Fig. 3:**
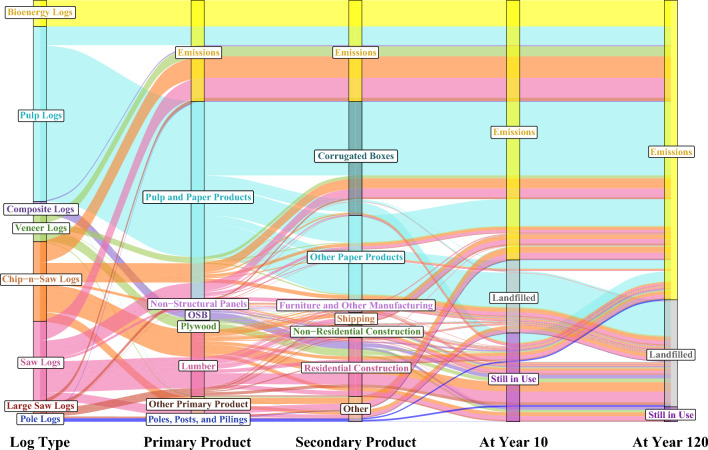
The flow of HWP carbon from southwide loblolly and shortleaf pine timber production

Using TPO harvest data for 2020 as inputs, we estimate that HWPs from loblolly and shortleaf pine timber harvested across the entire southern US store 29.7 MtC in the year they enter the market (year 0), 15.2 MtC after 10 years, and 11.4 MtC after 120 years in use and in landfills (Fig. 3) (Additional file 1: Table S26). Fossil fuel emissions from the procurement, transportation, and manufacturing of these wood products are estimated to be 43.3 MtCO_2_e in year 0. We estimate biogenic emissions from burned log carbon (e.g., pellets, residue burned at mills, etc.) in year 0 to be 34.5 MtCO_2_e. More carbon went into pulp and paper products than any other category of primary or secondary products (Table 3), though much of this carbon was quickly emitted or landfilled. Residential construction and landfills provided the most stable carbon storage over time (Table 3). The secondary product categories with the most carbon storage in year 0 were corrugated boxes, sanitary products, single family homes, packaging cartonboard, single family home upkeep, and disposable food related products (Additional file 1: Table S28).

**Table 3:** Stored HWP carbon in use from southwide loblolly and shortleaf timber by sector (MtC)

Sector	Year 0	Year 10	Year 120	Discounted sum over 120 years (3%)
Residential construction	5.5	5.4	1.4	148.4
Non-residential construction	1.2	1.2	< 0.1	27.5
Pulp and paper products	18.9	1.3	0	85.7
Shipping	1.1	< 0.1	0	3.9
Manufacturing	0.8	0.3	< 0.1	7.6
Other	2.1	0.3	< 0.1	12.2
Landfills	0.1	6.7	9.9	236.4

#### Carbon storage and emissions by log type and primary product

**Fig. 4:**
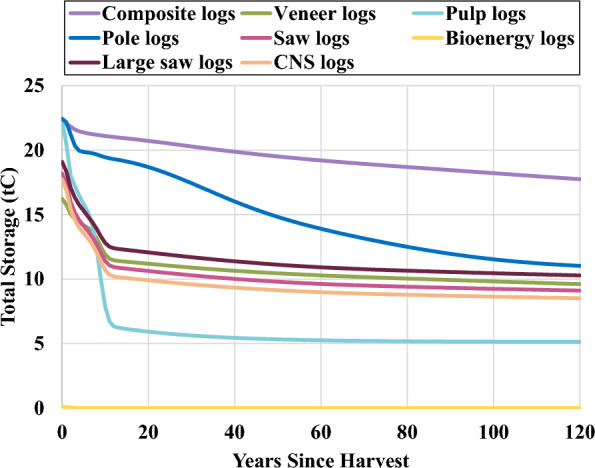
Total carbon storage (in use and in landfills) from 100 green tonnes of logs

**Fig. 5:**
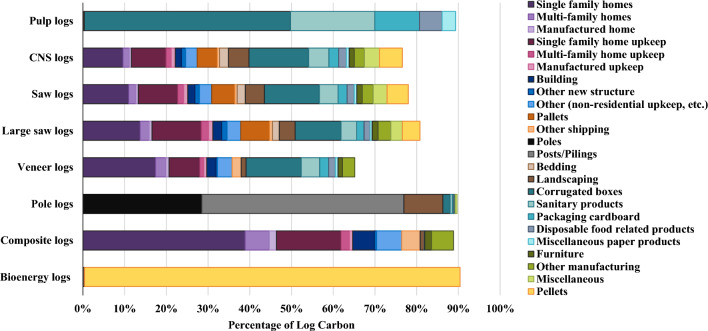
Percentage of carbon from harvested loblolly pine logs in secondary products by log type

Storage and emissions sums for each log type show that pulp logs have the greatest total emissions while composite logs, which are used to manufacture OSB and make up around 3% of southwide loblolly and shortleaf timber production, have the least (Table 4). Composite logs also store the most carbon compared to other log types per 100 green tonnes (Fig. 4; Additional file 1: Table S29). The advantage of composite logs can be attributed mainly to higher mill efficiency (35–50% for saw logs vs. 90% for composite logs), resulting in a higher portion of saw log carbon that is burned in year 0 (21–27% vs. 8%) or used for pulp and paper production (19–24% vs. < 1%) (Fig. 5). OSB is used more commonly in longer-lasting products than lumber (e.g., 48.5% of OSB is used in new residential construction vs. 31.7% of lumber), but the similar shape of the carbon storage curves after year 20 suggests that the secondary product mix is less important than mill efficiency and primary product mix. All solid wood primary products provided relatively stable carbon storage over time (Table 5). Engineered wood provided the most stable carbon storage due primarily to its high usage in new residential construction (80%), though this parameter value was based solely on the professional judgement of the authors. The portion of carbon remaining in other primary products, including lumber, plywood, OSB, non-structural panels, and pulp and paper, can be considered more certain.

**Table 4:** Carbon storage and emission pulses by log type

Log type	Total storage (tC)	CO_2_ emissions (biogenic) (tCO_2_e)	Methane emissions (biogenic) (tCO_2_e)	Total emissions (biogenic + FF) (tCO_2_e)
Pulp logs	285.4	− 60.1	− 6.4	− 108.9
CNS logs	359.9	− 53.8	− 2.0	− 73.4
Saw logs	381.7	− 51.6	− 2.0	− 70.7
Large saw logs	425.3	− 47.2	− 1.8	− 65.4
Veneer logs	392.6	− 50.2	− 1.9	− 74.6
Pole logs	587.0	− 56.4	− 1.1	− 68.1
Composite logs	681.7	− 20.2	− 0.8	− 41.4
Bioenergy logs	0.3	− 91.8	0.0	− 98.9

Sum of stored carbon (in use and in landfills) and GHG emission pulses from 100 green tonnes of timber over 120 years, discounted at 3%; FF = fossil fuel

**Table 5:** Portion of primary product carbon remaining in use and in landfills in year 100

Ranking	In use	In landfills	Total	Smith et al. estimation (total)
Lumber	0.184	0.470	0.654	0.639
Plywood	0.307	0.492	0.798	0.645
OSB	0.306	0.493	0.798	0.696
Non-structural panels	0.009	0.703	0.712	0.592
Engineered wood	0.487	0.377	0.862	–
Poles	0.082	0.509	0.555	–
Pulp and paper	0.000	0.228	0.228	0.151
Landscaping and Bedding	0.000	0.131	0.131	–
Miscellaneous	0.000	0.718	0.718	0.521

Estimates from Smith et al. [32], one of the most commonly cited wood product carbon studies [28, 31, 34, 37, 39, 69–72], are provided for comparison. Portions from year 100 are presented to match the timeframe of Smith et al. Estimates up to year 120 are provided in Additional file 1: Tables S33–35

### Sensitivity analysis

The results of the sensitivity analysis indicate relatively high influence for parameters related to short-lived products and landfills, including the fraction of organic carbon that decays in landfills, the portion of methane from MSW landfills that is oxidized and recovered, and parameters related to all life stages of corrugated boxes, including production, lifespan, recycling, and landfill decay (Table 6). Other influential parameters included those related to C&D waste, milling efficiency, and the lifespan of single family home upkeep and repair products. We estimate that total landfill emissions (CO_2_ + methane) account for around 9% of all discounted (3% rate) biogenic and fossil fuel emissions from loblolly and shortleaf pine harvested wood products over 120 years, and methane emissions from landfills account for around 4% (tCO_2_e). Though these percentages are relatively small, most of the parameters related to landfills within the proposed framework are for large categories of carbon (e.g., all landfilled solid wood products or all wood products in MSW landfills), intensifying the influence of these parameters compared to parameters that are specific to secondary or primary product categories.

**Table 6:** Sensitivity of emissions from loblolly and shortleaf pine wood products to the most influential parameters

Ranking	Parameter	Bounds	Units	µ	µ*
1	DOC_f_ for paper in MSW landfills	0.25–0.93	Portion	− 16.68	16.68
2	Portion of CH_4_ recovered (R) in MSW landfills	0.4019–0.8019	Portion	9.62	9.62
3	Lifespan of corrugated boxes	0.5–3	Years	8.29	8.29
4	Downcycling to non-structural manufacturing of C&D waste	0–0.129	Portion	− 4.84	4.84
5	Production FF for pulp and paper	0.3746–0.4579	tCO2e emitted/tCO2e in product	− 4.81	4.81
6	Portion of CH_4_ oxidized (OX) in MSW landfills	0.0221–0.4221	Portion	4.73	4.73
7	Average sawmill efficiency	0.3–0.5	Portion	4.45	4.45
8	DOC_f_ for wood in C&D landfills	0–0.5	Portion	− 4.19	4.19
9	Recycling rate for corrugated boxes	0.814–1	Portion	3.98	3.98
10	Recycling times for corrugated boxes	3–7	Times	3.97	3.97
11	Lifespan of single family home upkeep	12–62	Years	2.77	2.77
12	Pulp mill efficiency	0.79–0.99	Portion	− 2.68	2.68
13	Construction waste	0–0.156	Portion	− 2.61	2.61
14	DOC_f_ for wood in MSW landfills	0–0.5	Portion	− 2.49	2.49
15	Half-life of landfilled paper	12–18	Years	2.4	2.4

DOC_f_ = the fraction of landfilled organic carbon that will decay over time; MSW = municipal solid waste; C&D = construction and demolition; average sawmill efficiency = sawmill efficiency for 33–47 cm saw logs, with smaller logs having − 0.05 efficiency and larger logs having + 0.1 efficiency; the magnitude of µ* indicates the influence of the parameter; the sign of µ indicates the direction of the effect; the bounds indicate the tested range of parameter values (see “Sensitivity analysis” section); the model output of which sensitivity was evaluated was the total (biogenic + fossil fuel) annual emission pulses from southwide loblolly and shortleaf pine harvested wood products produced over 120 years, discounted at 3%; a table of all parameters tested and their sensitivity indices is available in Additional file 1: Table S36

A positive µ value generated by the sensitivity analysis indicates that increasing the value of the parameter will generate fewer emissions, and thus have climate benefits. For example, extending the lifespan of corrugated boxes or the half-life of landfilled paper products will have climate benefits. Conversely, a negative µ value indicates that decreasing the value of the parameter will have climate benefits. For example, decreasing the fraction of organic carbon that decays in landfills or lowering fossil fuel emission rates from pulp and paper production will have climate benefits. Interestingly, the results indicate that downcycling less C&D waste into manufacturing products (i.e., furniture and other manufacturing products) would result in climate benefits. This is likely due in part to the relatively short lifespans (7 and 13 years) of manufacturing products. However, because the samples are generated randomly within the Morris method global sensitivity analysis, the rates of other fates of C&D products (i.e., landfilled, downcycled to landscaping, and emitted) that were tested alongside fluctuating rates of downcycling to manufacturing are uncertain, and further analysis is required to analyze the interactions between these parameters.

### Silvicultural management scenarios

**Fig. 6:**
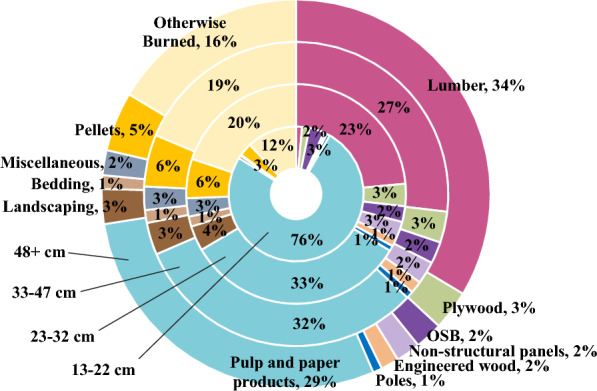
Percentage of harvested loblolly pine log carbon in primary products by diameter at breast height (DBH)

Because LobDSS generates outputs in terms of tons of logs per size class, regional cull factors were used to convert size classes into log types (Additional file 1: Table S17). After accounting for cull factors and mill residue use, 13–22 cm DBH logs had the highest portion of carbon enter the market as non-energy products, followed by 48+ cm DBH logs (Fig. 6).

**Fig. 7:**
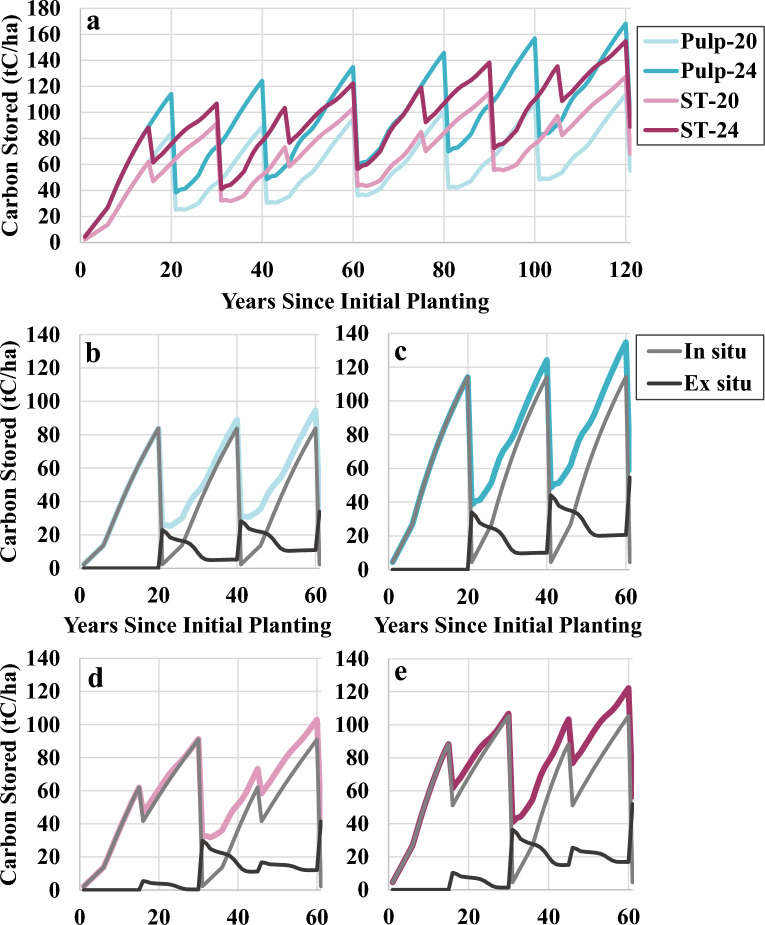
Total (colored), in situ (gray), and ex situ (black) carbon storage. **a** Four silvicultural management scenarios, **b** Pulp-20 (20-year rotation with SI-20 m), **c** Pulp-24 (20-year rotation with SI-24 m), **d** ST-20 (30-year rotation with SI-20 m), **e** ST-24 (30-year rotation with SI-24 m); (SI = site index for base age 25)

Our results show that on low productivity sites, the sawtimber regime stored more total carbon (in situ + ex situ) over 120 years (1649 tC ha^−1^) than the pulp regime (1449 tC ha^−1^) at a 3% discount rate. For high productivity sites, however, the pulp regime stored slightly more total carbon (2246 tC ha^−1^) than the sawtimber regime (2227 tC ha^−1^) (Fig. 7). In situ carbon storage was higher in the high productivity sites and sawtimber regimes, and ex situ carbon storage was higher in the high productivity sites and pulp regimes, as was total harvest tonnage (Table 7). Between the high productivity sites, the sawtimber regime produced slightly more lumber and substantially less pulp and paper over 120 years, compared to the pulp regime (Additional file 1: Table S39). While the portion of carbon that entered the market as longer-lived products increased for the sawtimber regimes compared to the pulp regimes on a per harvest basis, the high productivity pulp regime produced the highest total amount of carbon in longer-lived products over 120 years (Additional file 1: Table S40). In all scenarios, more carbon was used for corrugated boxes than any other secondary product, with corrugated boxes accounting for between 37 and 43% of all carbon that entered the market over 120 years (Additional file 1: Table S40). In all scenarios, ex situ storage accumulated over each rotation.

**Table 7:** Total harvested log tonnage over 120 years for four silvicultural management scenarios

Scenario	Pulp-20	Pulp-24	ST-20	ST-24
13–22 cm logs (DBH)	781	854	510	643
23–32 cm logs (DBH)	437	903	576	649
33–47 cm logs (DBH)	0	12	112	264
Total	1218	1769	1198	1556

DBH = diameter at breast height; 0% discount rate for harvest tonnage; values are in tonnes green weight hectare^−1^

### Wood usage scenarios

**Fig. 8:**
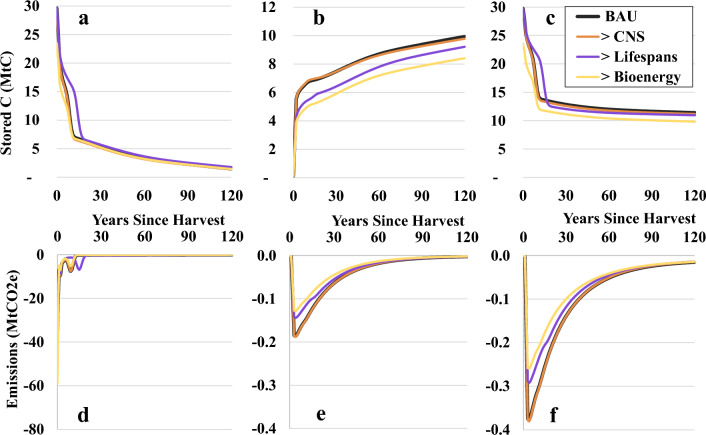
For each wood usage scenario: **a** carbon stored in use, **b** carbon stored in landfills, **c** total carbon storage, **d** biogenic CO_2_ emission pulses from wood products, **e** biogenic CO_2_ emission pulses from landfills, and **f** biogenic methane emission pulses from landfills

Most differences in storage and emission pulses between the wood usage scenarios took place within the first 20 years after harvest (Fig. 8). With the exception of pellets (+ 16.0%) and pulp and paper products (− 14.7%) in S3, no scenarios generated substantially different primary product mixes from the business as usual (BAU) scenario. S1 showed little change in either storage or emissions (Table 8). Product carbon remained in use for a longer period of time in S2, likely primarily due to the increased lifespan and recycling of corrugated boxes. Since less carbon overall entered the market as non-energy products in S3, biogenic emission pulses from products burned and decaying in landfills were 15.2 MtCO_2_e lower, with the exception of year 0 in which the pellets were burned and biogenic emissions increased by 22.6 MtCO_2_e. Additionally, while carbon that was diverted to pellets in S3 was emitted as CO_2_, a portion of that carbon in the BAU scenario was emitted as methane (5.8 MtCO_2_e) from products decaying in landfills, resulting in higher biogenic emissions in the BAU scenario from the same amount of log carbon. Fossil fuel emissions were lower in S3 than in the BAU scenario (− 9.2 MtCO_2_e), primarily due to the lower fossil fuel consumption rates associated with pellet production (0.3179 tCO_2_e emissions/tC product) as compared to pulp and paper production (1.8992 tCO_2_e emissions/tC product) [73, 74].

**Table 8:** Difference (Δ) in stored carbon and GHG emission pulses between BAU and wood usage scenarios

Scenario Scenario No	BAU	> CNS (S1)	> Lifespans (S2)	> Bioenergy (S3)
Storage in use (MtC)	285.3	− 8.2	+ 55.8	− 32.2
Storage in landfill (MtC)	236.4	+ 0.2	− 34.0	− 51.1
Total storage (MtC)	521.1	− 8.0	+ 20.6	− 83.3
Biogenic CO_2_ emission pulses from products (MtCO_2_e)	− 87.2	+ 0.7	+ 3.2	+ 10.0
Biogenic CO_2_ emission pulses from landfills (MtCO_2_e)	− 2.7	+ 0–0.1	− 0.5	− 0.8
Biogenic CH_4_ emission pulses from landfills (MtCO_2_e)	− 5.8	+ 0–0.1	− 1.1	− 1.7
Fossil fuel emissions (MtCO_2_e)	− 43.3	+ 0.3	0	− 9.2
Total emissions (MtCO_2_e)	− 139.0	+ 1.1	+ 1.6	− 1.8

BAU = business as usual; CNS = chip-n-saw; BAU values are sums of annual storage or emission pulse values over 120 years, assuming the default parameter values for LobWISE, discounted at 3%; values presented for S1, S2, and S3 (right side columns) indicate the change (Δ) in the absolute value of the presented BAU value (e.g., we estimate that storage in use was decreased by 8.2 MtC and CO_2_ emissions from products were increased by 0.7 MtCO_2_e in S1 over 120 years)

## Discussion

Results from this analysis suggest that the combined carbon storage of pine plantations and resulting HWPs have the potential to grow over time, despite the temporary loss of carbon storage on forested stands that results from harvests. Many studies have come to similar conclusions for both southern pine [8, 23, 75, 76] and other forest types [77–79]. However, we find that future carbon storage and emissions are sensitive to both silvicultural management and parameters along the wood product flow. Our results suggest that adjusting rotation length based on individual site productivity, as well as extending the storage of carbon in key products, such as corrugated boxes and residential upkeep products, and in landfills could result in substantial carbon gains.

Longer rotations are frequently recommended as a strategy for increasing both in situ and ex situ carbon in even-aged stands [8, 75, 80, 81] due to the amount of in situ carbon that is emitted soon after each harvest (i.e., slash, roots, competing vegetation) and the higher portion of long rotation harvests that are saw logs, resulting in more long-term ex situ storage. Our findings similarly suggest that longer rotations (30 years) may be advantageous when site productivity is low (20 SI vs. 24 SI). However, they also indicate that shorter rotations (20 years) may be advantageous under certain conditions, such as high site productivity, suggesting that landowners hoping to optimize total forest carbon storage should make site specific management decisions, rather than opting for a longer rotation in all cases. Between silvicultural management scenarios with the same rotation lengths, sites with higher productivity had higher total carbon storage. Gonzalez-Benecke et al. [8] similarly found that lowering site index to 15 m from a default of 22 m resulted in 28% less net (in situ + ex situ—fossil fuel emissions from silviculture) carbon storage for loblolly pine, while increasing site index to 30 m resulted in 38% more storage. This finding suggests possible benefits of using silviculture or genetics to improve site productivity.

We also identify several changes within the wood product flow that could lead to significant carbon gains if realized, including reducing decay from landfills, increasing methane capture and oxidation in MSW landfills, extending the lifespan of corrugated boxes and residential upkeep and repair products, recycling corrugated boxes at a higher rate and more times, increasing the mill efficiency of saw logs, and favoring composite and CNS logs over veneer and higher-diameter saw logs. Despite the importance of landfill decay parameters, such as the fraction of organic carbon in various materials that decays under anaerobic landfill conditions (DOC_f_), few studies have quantified these parameters for individual materials [82, 83]. IPCC guidance recommends a default DOC_f_ factor of 0.5 for all materials [38], but studies have shown wide ranges in this factor, depending on the material, from 0–3% in solid wood [52] to 93% in copy paper [82]. Further scientific research on this topic could benefit GHG emission estimations from landfills and enhance the understanding of wood product carbon flows.

According to our findings, corrugated boxes represent the largest portion of new product carbon from real southern US loblolly and shortleaf harvests, as well as all simulated silvicultural management scenarios. According to the US EPA, corrugated boxes make up 11.4% of all US MSW waste generation, including non-wood materials [51]. Most literature surrounding ex situ carbon uses 1–3 years as the lifespan for all paper products, including corrugated boxes [8, 10, 32, 36]. The US EPA categorizes corrugated boxes as “containers and packaging,” which are assumed to be discarded in the year they are purchased [84]. Corrugated boxes are generally considered to have one of the highest rates of recycling of any material in the US, with rate estimates ranging from 91% [85] to 96% [84]. Even with already high rates, we estimate that further improvements in corrugated box recycling would result in substantial carbon gains. Several studies have found that increasing recycling rates of various products, including certain paper products, leads to minimal GHG emission reductions and may even increase GHG emissions due to economic ramifications, causing production of additional primary products, and to additional emissions involved with recycling and product loss during the recycling process [86–88]. These contradictory results highlight the complexity of waste systems and the need to incorporate economic responses into future studies.

Results of the sensitivity analysis also indicated sensitivity to mill efficiency for saw and pulp logs. While pulp mill residues are exclusively bark that cannot be made into paper products, residues from saw mills include bark, coarse residue, and fine residue, and are affected by log attributes (diameter, length, taper, defects, etc.), scanning and sawing machinery, and product mix [89]. Giasson et al. [90] also found sawmill efficiency to be an important factor in reducing emissions from softwood products. Estimates for sawmill efficiencies range from 36% [45] to 46% [91], and these efficiencies increase over time as sawmill technology develops. Milota et al. [91] surveyed sawmills in the southern US and found that over half of the mills had upgrades to scanning, optimization, grading, or planer systems in the few years preceding the survey. It is assumed in this study that larger logs have higher conversion efficiencies than smaller logs, but the degree to which this is true is not well studied [89]. Many contemporary mills retain data on both log characteristics and lumber recovery factors, and these data could potentially be used to analyze the relationship between log attributes and mill efficiency.

Smith et al. [32] is one of the most commonly cited studies for the portion of wood remaining in wood products after a period of time, especially when regional values are used [28, 31, 34, 37, 39, 69–72]. In comparison to estimates from this study, we found carbon remaining in use in year 100 from primary products to be slightly lower, while carbon remaining in landfills and total carbon remaining were slightly higher (Additional file 1: Tables S33–35). The most stable primary products were engineered wood, plywood, and OSB. Since composite logs, which are used to manufacture OSB, have the highest mill efficiency rate (90%) of any non-energy log type, and since composite logs can generally be grown in short rotations, OSB has potential as a more efficient method of carbon storage than similar products, such as plywood. Lumber also provides relatively stable carbon storage, with an estimated 65% remaining stored in year 100 (Smith et al. [32] estimate 64%). CNS logs, much like OSB logs, can be grown more quickly than higher diameter saw logs to produce lumber, and the minimal effects of increased CNS consumption (− 8.3 MtC of total storage over 120 years) in the wood usage scenarios (S1) points to another product shift with carbon storage potential.

### Limitations and future directions

While fossil fuel emissions from silvicultural activities such as planting, fertilizing, applying herbicide, and harvesting are generally considered to be low, compared to the resulting in situ storage gains [8], fossil fuel emissions from transportation and especially production of wood products are significant when compared to the carbon benefits of the resulting products [35, 73, 74, 91–101]. We have accounted for fossil fuel emissions in the silvicultural management scenarios by deducting the tC equivalent to the CO_2_ remaining in the atmosphere for a given year from the storage in wood products for that year. However, one tC stored in wood products for one year is not equivalent to one tC emitted to the atmosphere, since emitted carbon will continue to have radiative forcing effects on the atmosphere for many years. Rather, emissions should be contrasted to sequestration, which actively pulls carbon out of the atmosphere and thus prevents future radiative forcing effects. Levasseur et al. [60] propose using dynamic carbon accounting to compare each pulse of sequestration and emissions in the year in which they occur. This method can better analyze the temporal tradeoffs that exist in forest and wood product systems and should be incorporated into further analysis.

Furthermore, we do not incorporate any substitution effects from using wood products rather than alternative construction materials (e.g., concrete, steel, etc.) or deriving energy from wood pellets, mill residue, wood waste, or landfill methane rather than from fossil fuels into our analyses or the proposed framework. Quantifying the potential GHG implications of forest-based bioenergy and wood products substitution would require an integrated assessment approach, with coverage of energy, industry, and construction sectors and a carefully designed counterfactual scenario. Such an analysis could also require a more involved LCA of alternative construction materials that additional wood would replace. While beyond the scope of this analysis, further research is needed to understand the full potential GHG benefits (or costs) of wood product substitution. Despite these limitations, the framework presented in this manuscript provides a detailed tool to support economic or policy analysis on the benefits of wood use over other construction, energy, and related products. Fossil fuel emissions are also not included in the proposed framework for the recycling process, construction, or any other process in a product’s lifetime after the mill.

The proposed framework can be used to assess carbon storage in HWPs under various hypothetical scenarios. In most of our analyses, we assumed constant cull factors, tonnage of log types produced, secondary product mix, and total harvest volume for the southern US. However, market and policy conditions that affect forest management, HWP production and consumption, and land use all influence these parameters, and analyses that consider these effects are essential to understanding the total carbon contribution of southern US pine plantations.

Finally, the silvicultural management scenarios represent only four silvicultural management alternatives based on static, modeled in situ carbon estimates. Further analysis is needed to understand the effects of other silvicultural decisions, such as herbicide and fertilizer application, variable planting densities, and genetic material; to include carbon pools such as root and harvest residue carbon; to test LobWISE in combination with empirical in situ carbon data; to understand the effect that climate change and rising atmospheric CO_2_ levels will have on forest carbon sequestration and harvest outputs; and to understand the effects of spatial parameters on total carbon storage.

## Conclusions

This study uses elements of a LCA approach to develop and parameterize a detailed framework that models carbon in wood products sourced from managed loblolly pine plantation systems, which are a critical input to the global forest products sector. We evaluate carbon storage performance of various product allocation and silvicultural management scenarios, as well as parametric sensitivity analysis to understand key drivers of variability in emissions projections. The framework provides a flexible decision support tool that practitioners can use to evaluate potential emissions implications of both silvicultural and end product allocation decisions, which can support carbon offset project and corporate insetting strategy development, as well as informing policy design around land- and bioproduct-based mitigation strategies.

Our findings also have relevance to the broader scientific literature on wood product carbon storage. Although we do not account for the GHG emissions displacement potential of wood products and bioenergy, our results show lasting GHG benefits from wood products sourced from southern loblolly pine systems. While our methodology differs substantially from Peng et al. [11], our results similarly point to the important role that HWPs play in the long-term GHG emissions profile of the wood products sector.

Also much of the literature surrounding HWP carbon storage is focused on silvicultural management regimes that produce saw logs and on longer-lasting products, such as housing and buildings [6]. This focus on longer-lived wood product carbon storage is consistent with research focus on reducing permanence risk of in situ forest carbon management or arguing in favor of climate solutions with long-term carbon storage potential [102]. This perspective ignores the important potential role of temporary carbon storage benefits of forest management interventions [59, 103].

However, our research findings emphasize the need for a better understanding of carbon flows in short-lived products, such as corrugated boxes, and in landfills. Our findings also emphasize the role of chip-n-saw logs as a potential source of lumber that allows for more efficient carbon sequestration and storage without significantly increasing emissions near- and long-term. The proposed framework can also be linked with growth and yield models for further analysis involving optimization of forest management and product allocation strategies for carbon storage and wood product supply.

### Supplementary Information

**Additional file 1: Text 1**. Descriptions of secondary product categories used in LobWISE and sources. **Table S1**. General end use category by primary product. **Table S2**. Specific end use category by primary product and general end use. **Table S3**. Lifespans of single family housing parts. **Table S4**. Lifespans of multi-family housing parts. **Table S5**. Lifespans of other products. **Table S6**. Portion of transitioned product that is landfilled. **Table S7**. Portion of transitioned product that is downcycled as landscaping. **Table S8**. Portion of transitioned product that is downcycled as bedding. **Table S9**. Portion of transitioned product that is downcycled as posts and pilings. **Table S10**. Portion of transitioned product that is downcycled as structural panels. **Table S11**. Portion of transitioned product that is downcycled as non-structural panels. **Table S12**. Fossil fuel emission factors. **Table S13**. Landfill decay parameters. **Table S14**. Landfill methane parameters. **Table S15**. Waste deduction parameters. **Table S16**. Portion of primary product carbon used to produce engineered wood. **Table S17**. Portion of carbon in logs of each size class that is used as each log type (i.e., cull factors). **Table S18**. Portion of log carbon used in primary products and mill residue. **Table S19**. Mill residue use. **Table S20**. “Fiber product” mill residue use. **Table S21**. Specific gravity of loblolly pine wood. **Table S22**. Recycling parameters. **Table S23**. Primary product carbon usage in housing (structural vs. non-structural). **Table S24**. Portion of structural housing carbon in each application. **Table S25**. Portion of non-structural housing carbon in each application. **Table S26**. Carbon storage and pulse emissions from loblolly and shortleaf pine timber harvested in 2020. **Table S27**. Division of carbon from loblolly and shortleaf pine timber harvested in 2020 by primary product category. **Table S28**. Division of carbon from loblolly and shortleaf pine timber harvested in 2020 by secondary product category. **Table S29**. Total carbon storage from 100 green tonnes of loblolly pine logs (tC). **Table S30**. Annual biogenic CO_2_ pulse emissions from 100 green tonnes of loblolly pine logs (tCO_2_e). **Table S31**. Annual biogenic CH_4_ pulse emissions from 100 green tonnes of loblolly pine logs (tCO_2_e). **Table S32**. Total biogenic and fossil fuel annual pulse emissions from 100 green tonnes of loblolly pine logs (tCO_2_e). **Table S33**. Portion of primary product carbon remaining in use up to 120 years after production. **Table S34**. Portion of primary product carbon remaining in landfills up to 120 years after production. **Table S35**. Portion of primary product carbon remaining in use and in landfills up to 120 years after production. **Table S36**. Sensitivity of emissions from loblolly and shortleaf pine wood products to all tested parameters. **Table S37**. Carbon storage from four silvicultural management scenarios, 60 years since initial planting (tC ha^−1^). **Table S38**. Carbon storage from four silvicultural management scenarios, 120 years since initial planting (tC ha^−1^). **Table S39**. Total carbon that enters the market or is burned by primary product for four silvicultural management scenarios (tC ha^−1^). **Table S40**. Total carbon that enters the market by secondary product category for four silvicultural management scenarios (tC ha^−1^). **Table S41**. Carbon storage in use from three wood usage scenarios and a business as usual (BAU) scenario (MtC). **Table S42**. Carbon storage in landfills from three wood usage scenarios and a business as usual (BAU) scenario (MtC). **Table S43**. Total carbon storage from three wood usage scenarios and a business as usual (BAU) scenario (MtC). **Table S44**. Biogenic CO_2_ emissions from wood products (excluding landfills) from three wood usage scenarios and a business as usual (BAU) scenario (MtCO_2_e). **Table S45**. Biogenic CO_2_ emission pulses from landfills from three wood usage scenarios and a business as usual (BAU) scenario (MtCO_2_e). **Table S46**. Biogenic CH_4_ emission pulses from landfills from three wood usage scenarios and a business as usual (BAU) scenario (MtCO_2_e). **Table S47**. Fossil fuel emission pulses from procurement, transportation, and production from three wood usage scenarios and a business as usual (BAU) scenario (MtCO_2_e). **Table S48**. Total (biogenic + fossil fuel) emission pulses from three wood usage scenarios and a business as usual (BAU) scenario (MtCO_2_e). **Figure S1**. Flow of carbon from primary products to eventual emission. Shows the transition to secondary products, recycling, downcycling, landfilling, and emission.

## Data Availability

US Forest Service Timber Products Output data are available from https://www.fia.fs.usda.gov/program-features/tpo/ (last access August 8, 2023). RISI Fastmarkets data were accessed from https://www.risiinfo.com/millassets/millLanding.html, but are not publicly available. FIA data used to determine the diameter distribution of saw logs is available from https://apps.fs.usda.gov/fiadb-api/evalidator. Other model inputs and parameter assumptions are documented in this manuscript and in Additional file 1, and the Excel framework can be made available for research purposes on request.

## Abbreviations

BAU: Business as usual
DOC_f_: Fraction of decayable organic carbon in landfills
C&D: Construction and demolition
CNS: Chip-n-saw
DBH: Diameter at breast height
EPA: Environmental Protection Agency
GHG: Greenhouse gas
HWP: Harvested wood product
LCA: Life cycle assessment
LobWISE: Loblolly Wood Inventory, Storage, and Emissions
MSW: Municipal solid waste
OSB: Oriented strand board
SI: Site index
TPO: Timber Products Output

## References

[1] IPCC. Summary for policymakers. Clim Change 2022 Mitig Clim Change; 2022

[2] Harris NL, Gibbs DA, Baccini A, Birdsey RA, De Bruin S, Farina M (2021). Global maps of twenty-first century forest carbon fluxes. Nat Clim Change.

[3] Daigneault A, Baker JS, Guo J, Lauri P, Favero A, Forsell N (2022). How the future of the global forest sink depends on timber demand, forest management, and carbon policies. Glob Environ Change.

[4] Favero A, Yoo J, Daigneault A, Baker J (2023). Temperature and energy security: will forest biomass help in the future?. Clim Change Econ.

[5] Kim SJ, Baker JS, Sohngen BL, Shell M (2018). Cumulative global forest carbon implications of regional bioenergy expansion policies. Resour Energy Econ.

[6] Mishra A, Humpenöder F, Churkina G, Reyer CPO, Beier F, Bodirsky BL (2022). Land use change and carbon emissions of a transformation to timber cities. Nat Commun.

[7] Fargione JE, Bassett S, Boucher T, Bridgham SD, Conant RT, Cook-Patton SC (2018). Natural climate solutions for the United States. Sci Adv.

[8] Gonzalez-Benecke CA, Martin TA, Jokela EJ, Torre RDL (2011). A flexible hybrid model of life cycle carbon balance for loblolly pine (*Pinus taeda* L.) management systems. Forests.

[9] Haight RG, Bluffstone R, Kline JD, Coulston JW, Wear DN, Zook K (2020). Estimating the present value of carbon sequestration in U.S. forests, 2015–2050, for evaluating federal climate change mitigation policies. Agric Resour Econ Rev.

[10] Johnston CMT, Radeloff VC (2019). Global mitigation potential of carbon stored in harvested wood products. Proc Natl Acad Sci.

[11] Peng L, Searchinger TD, Zionts J, Waite R (2023). The carbon costs of global wood harvests. Nature.

[12] Baker JS, Wade CM, Sohngen BL, Ohrel S, Fawcett AA (2019). Potential complementarity between forest carbon sequestration incentives and biomass energy expansion. Energy Policy.

[13] Wade CM, Baker JS, Jones JPH, Austin KG, Cai Y, De Hernandez AB (2022). Projecting the impact of socioeconomic and policy factors on greenhouse gas emissions and carbon sequestration in U.S. Forestry and Agriculture. J For Econ.

[14] Shephard NT, Narine L, Peng Y, Maggard A (2022). Climate smart forestry in the Southern United States. Forests.

[15] Johnston CMT, Guo J, Prestemon JP (2023). RPA forest products market data for U.S. RPA Regions and the world, historical (1990–2015), and projected (2020–2070) using the Forest Resource Outlook Model (FOROM).

[16] Skog KE (2008). Sequestration of carbon in harvested wood products for the United States. For Prod J.

[17] EPA. Inventory of U.S. greenhouse gas emissions and sinks: 1990–2021. U.S. Environmental Protection Agency, EPA; 2023. Report No.: EPA 430-R-23-002.

[18] Galik CS, Mobley ML, de Richter DB (2009). A virtual “field test” of forest management carbon offset protocols: the influence of accounting. Mitig Adapt Strateg Glob Change.

[19] Richards KR, Huebner GE (2012). Evaluating protocols and standards for forest carbon-offset programs, Part B: leakage assessment, wood products, validation and verification. Carbon Manag.

[20] Cabiyo B, Fried JS, Collins BM, Stewart W, Wong J, Sanchez DL (2021). Innovative wood use can enable carbon-beneficial forest management in California. Proc Natl Acad Sci.

[21] FSC-US Forest Management Standard (V1.1); 2019. https://us.fsc.org/en-us/certification/forest-management-certification

[22] SFI 2022: Standards and Rules; 2022. https://forests.org/new-sfi-2022-standards-updates/

[23] Fuller M, Dwivedi P (2021). The cost of carbon stored on afforested lands in the southern United States. Trees For People.

[24] Krankina ON, Harmon ME, Schnekenburger F, Sierra CA (2012). Carbon balance on federal forest lands of Western Oregon and Washington: the impact of the Northwest Forest Plan. For Ecol Manag.

[25] Lippke B, Puettmann M, Oneil E, Dearing OC (2021). The plant a trillion trees campaign to reduce global warming—fleshing out the concept. J Sustain For.

[26] UNFCCC. Race to Zero Lexicon; 2021. https://unfccc.int/climate-action/race-to-zero-campaign

[27] Brunet-Navarro P, Jochheim H, Muys B (2016). Modelling carbon stocks and fluxes in the wood product sector: a comparative review. Glob Change Biol.

[28] CARB (2014). Compliance offset protocol: U.S. forest projects.

[29] Houghton RA, Hobbie JE, Melillo JM, Moore B, Peterson BJ, Shaver GR (1983). Changes in the carbon content of terrestrial biota and soils between 1860 and 1980: a net release of CO"2 to the atmosphere. Ecol Monogr.

[30] Plantinga AJ, Birdsey RA (1993). Carbon fluxes resulting from U.S. private timberland management. Clim Change.

[31] Stockmann KD, Anderson NM, Skog KE, Healey SP, Loeffler DR, Jones G (2012). Estimates of carbon stored in harvested wood products from the United States forest service northern region, 1906–2010. Carbon Balance Manag.

[32] Smith JE, Heath LS, Skog KE, Birdsey RA. Methods for calculating forest ecosystem and harvested carbon with standard estimates for forest types of the United States. Newtown Square, PA: U.S. Department of Agriculture, Forest Service, Northeastern Research Station; 2006 p. NE-GTR-343. Report No.: NE-GTR-343. https://www.fs.usda.gov/treesearch/pubs/22954

[33] VCS VCS. VCS Module VMD0026: Estimation of carbon stocks in the long lived wood products pool. The Earth Partners LLC; 2012. https://verra.org/methodologies/vmd0026-estimation-of-carbon-stocks-in-the-long-lived-wood-products-pool-v1-0/

[34] Hennigar C, Amos-Binks L, Cameron R, Gunn J, MacLean DA, Twery M. ForGATE—a forest sector greenhouse gas assessment tool for maine: calibration and overview. Newtown Square, PA: U.S. Department of Agriculture, Forest Service, Northern Research Station; 2013 [cited 2022 Dec 12] p. NRS-GTR-116. Report No.: NRS-GTR-116. https://www.nrs.fs.usda.gov/pubs/43540

[35] Ganguly I, Pierobon F, Sonne HE (2020). Global warming mitigating role of wood products from Washington state’s private forests. Forests.

[36] Pingoud K, Skog KE. 2006 IPCC guidelines for national greenhouse gas inventories: harvested wood products. 2006. Report No.: Volume 4: Chapter 12. https://www.ipcc-nggip.iges.or.jp/public/2006gl/vol4.html

[37] Hoover CM, Beukema SJ, Robinson DCE, Kellock KM, Abraham DA. PRESTO: online calculation of carbon in harvested wood products. Newtown Square, PA: U.S. Department of Agriculture, Forest Service, Northern Research Station; 2014. p. NRS-GTR-141. Report No.: NRS-GTR-141. Available from: https://www.nrs.fs.usda.gov/pubs/47240. Accessed 21 Dec 2022.

[38] Pingoud K, Skog KE. 2006 IPCC guidelines for national greenhouse gas inventories: solid waste disposal; 2006. Report No.: Volume 5: Chapter 3. https://www.ipcc-nggip.iges.or.jp/public/2006gl/vol5.html

[39] Hoover C, Birdsey R, Goines B, Lahm P, Marland G, Nowak D, et al. Chapter 6: quantifying greenhouse gas sources and sinks in managed forest systems. 2014; Technical bulletin number 1939.

[40] Lemprière TC, Kurz WA, Hogg EH, Schmoll C, Rampley GJ, Yemshanov D (2013). Canadian boreal forests and climate change mitigation. Environ Rev.

[41] Smith WB, Miles P, Perry C, Pugh S (2009). Forest resources of the United States.

[42] Anderson PH, Johnsen KH, Butnor JR, Gonzalez-Benecke CA, Samuelson LJ (2018). Predicting longleaf pine coarse root decomposition in the southeastern US. For Ecol Manag.

[43] Milota MR (2005). Gate-to-gate life-cycle inventory of softwood lumber production. WOOD FIBER Sci.

[44] Gaby LI. The Southern Pines. US For Serv; 1985. FS-256.

[45] USFS. Timber products output; 2020. https://www.fia.fs.usda.gov/program-features/tpo/

[46] Brandeis C, Taylor M, Abt KL, Alderman D, Buehlmann U. Status and trends for the U.S. forest products sector: a technical document supporting the forest service 2020 RPA assessment. Asheville, NC: U.S. Department of Agriculture, Forest Service, Southern Research Station; 2021. p. SRS-GTR-258. Report No.: SRS-GTR-258. https://www.fs.usda.gov/treesearch/pubs/61862. Accessed 7 Nov 2022.

[47] US EPA, Zimmer T, Weitz K, Padhye A, Sifleet S. Wood waste inventory: final report. 2018. Report No.: EPA/600/R-18/262.

[48] Ianchenko A, Simonen K, Barnes C (2020). Residential building lifespan and community turnover. J Archit Eng.

[49] Arehart JH, Pomponi F, D’Amico B, Srubar WV (2022). Structural material demand and associated embodied carbon emissions of the United States building stock: 2020–2100. Resour Conserv Recycl.

[50] Pingoud K, Wagner F (2006). Methane emissions from landfills and carbon dynamics of harvested wood products: the first-order decay revisited. Mitig Adapt Strateg Glob Change.

[51] EPA. Advancing sustainable materials management: 2018 tables and figures; 2020. (Assessing trends in materials generation and management in the United States). https://www.epa.gov/facts-and-figures-about-materials-waste-and-recycling/advancing-sustainable-materials-management

[52] Micales JA, Skog KE (1997). The decomposition of forest products in landfills. Int Biodeterior Biodegrad.

[53] Morris MD (1991). Factorial sampling plans for preliminary computational experiments. Taylor Francis Ltd Am Stat Assoc.

[54] Di Lullo G, Gemechu E, Oni AO, Kumar A (2020). Extending sensitivity analysis using regression to effectively disseminate life cycle assessment results. Int J Life Cycle Assess.

[55] Campolongo F, Cariboni J, Saltelli A (2007). An effective screening design for sensitivity analysis of large models. Environ Model Softw.

[56] Saltelli A, Ratto M, Andres T, Campolongo F, Cariboni J, Gatelli D (2008). Global sensitivity analysis: the primer.

[57] Young S. Memorandum for the Heads of Executive Departments and Agencies: 2023 Discount Rates for OBM Circular N0. A-94; 2023. (Guidelines and Discount Rates for Benefit-Cost Analysis of Federal Programs). Report No.: OMB Circular No. A-94.

[58] Nordhaus W (2014). Estimates of the social cost of carbon: concepts and results from the DICE-2013R model and alternative approaches. J Assoc Environ Resour Econ.

[59] Parisa Z, Marland E, Sohngen B, Marland G, Jenkins J (2022). The time value of carbon storage. For Policy Econ.

[60] Levasseur A, Lesage P, Margni M, Deschênes L, Samson R (2010). Considering time in LCA: dynamic LCA and its application to global warming impact assessments. Environ Sci Technol.

[61] USFS. Forest Inventory and Analysis: Average annual harvest removals of sawlog volume of sawtimber trees on timberland; 2013. https://apps.fs.usda.gov/fiadb-api/evalidator. Accessed 1 Jun 2023.

[62] Amateis RL, Burkhart HE. FASTLOB 3.1: A stand-level growth and yield model for fertilized and thinned loblolly pine plantations; 2014.

[63] Montes CR (2001). A silvicultural decision support system for loblolly pine plantations.

[64] Peay WS, Bullock BP, Montes CR. Growth and yield model comparisons for mid-rotation loblolly pine (*Pinus taeda* L.) plantations in the southeastern US. Fassnacht F, editor. For Int J For Res. 2022;14:616–33.

[65] Butnor JR, Johnsen KH, Sanchez FG, Nelson CD (2012). Impacts of pine species, stump removal, cultivation, and fertilization on soil properties half a century after planting. Can J For Res.

[66] Johnson DW, Knoepp JD, Swank WT, Shan J, Morris LA, Van Lear DH (2002). Effects of forest management on soil carbon: results of some long-term resampling studies. Environ Pollut.

[67] Vogel JG, Bracho R, Akers M, Amateis R, Bacon A, Burkhart HE (2021). Regional assessment of carbon pool response to intensive silvicultural practices in loblolly pine plantations. Forests.

[68] Myhre G, Shindell D, Bréon FM, Collins W, Fuglestvedt J, Huang J, et al. Anthropogenic and Natural Radiative Forcing Supplementary Material. IPCC; 2013. (Climate Change 2013: The Physical Science Basis. Contribution of Working Group I to the Fifth Assessment Report of the Intergovernmental Panel of Climate Change). www.climatechange2013.org and www.ipcc.ch

[69] Law BE, Waring RH (2015). Carbon implications of current and future effects of drought, fire and management on Pacific Northwest forests. For Ecol Manag.

[70] Hoover C, Rebain S. The Kane Experimental Forest Carbon Inventory: Carbon Reporting with FVS. In: USDA Forest Service Proceedings; 2008. (RMRS-P-54).

[71] Heath LS, Nichols MC, Smith JE, Mills JR. FORCARB2: An updated version of the U.S. Forest Carbon Budget Model. Newtown Square, PA: U.S. Department of Agriculture, Forest Service, Northern Research Station; 2010. p. NRS-GTR-67. Report No.: NRS-GTR-67. https://www.fs.usda.gov/research/treesearch/35613. Accessed 13 Jan 2024.

[72] Loeffler D, Anderson N, Stockmann K, Skog K, Healey S, Jones JG, et al. Estimates of carbon stored in harvested wood products from United States Forest Service Southern Region, 1911–2012. 2014; 10.1186/1750-0680-7-1 PMC3276408 22244260

[73] Morrison B, Golden JS (2018). Southeastern United States wood pellets as a global energy resource: a cradle-to-gate life cycle assessment derived from empirical data. Int J Sustain Energy.

[74] Tomberlin KE, Venditti R, Yao Y (2020). Life cycle carbon footprint analysis of pulp and paper grades in the United States using production-line-based data and integration. BioResources.

[75] Sohngen B, Brown S (2008). Extending timber rotations: carbon and cost implications. Clim Policy.

[76] Jonker JGG, Van Der Hilst F, Markewitz D, Faaij APC, Junginger HM (2018). Carbon balance and economic performance of pine plantations for bioenergy production in the Southeastern United States. Biomass Bioenergy.

[77] De Rosa M, Schmidt J, Brandão M, Pizzol M (2017). A flexible parametric model for a balanced account of forest carbon fluxes in LCA. Int J Life Cycle Assess.

[78] Baul TK, Alam A, Strandman H, Seppälä J, Peltola H, Kilpeläinen A (2020). Radiative forcing of forest biomass production and use under different thinning regimes and initial age structures of a Norway spruce forest landscape. Can J For Res.

[79] Heath LS, Smith JE, Woodall CW, Azuma DL, Waddell KL (2011). Carbon stocks on forestland of the United States, with emphasis on USDA Forest Service ownership. Ecosphere.

[80] Nepal P, Grala RK, Grebner DL (2012). Financial feasibility of increasing carbon sequestration in harvested wood products in Mississippi. For Policy Econ.

[81] Parajuli R, Chang SJ (2012). Carbon sequestration and uneven-aged management of loblolly pine stands in the Southern USA: a joint optimization approach. For Policy Econ.

[82] Wang X, De la Cruz FB, Ximenes F, Barlaz MA (2015). Decomposition and carbon storage of selected paper products in laboratory-scale landfills. Sci Total Environ.

[83] Krause MJ (2018). Intergovernmental panel on climate change’s landfill methane protocol: Reviewing 20 years of application. Waste Manag Res J Sustain Circ Econ.

[84] EPA. Advancing sustainable materials management: 2018 Fact Sheet; 2020.

[85] AF&PA. How Does AF&PA Calculate Paper and Cardboard Recycling Rates?. 2022. https://www.afandpa.org/news/2022/how-does-afpa-calculate-paper-and-cardboard-recycling-rates. Accessed 1 Jun 2023.

[86] Zink T, Geyer R, Startz R (2016). A market-based framework for quantifying displaced production from recycling or reuse. J Ind Ecol.

[87] Levis JW, Barlaz MA, De Carolis JF, Ranjithan SR (2014). Systematic exploration of efficient strategies to manage solid waste in U.S. municipalities: perspectives from the solid waste optimization life-cycle framework (SWOLF). Environ Sci Technol.

[88] Chang JC, Beach RH, Olivetti EA (2019). Consequential effects of increased use of recycled fiber in the United States pulp and paper industry. J Clean Prod.

[89] Steele PH. Factors determining lumber recovery in sawmilling. Madison, WI: U.S. Department of Agriculture, Forest Service, Forest Products Laboratory; 1984. p. FPL-GTR-39. Report No.: FPL-GTR-39. https://www.fs.usda.gov/treesearch/pubs/8907. Accessed 7 Nov 2022.

[90] Giasson LA, Thiffault E, Lebel L, Carle JF (2023). Carbon balance of forest management and wood production in the boreal forest of Quebec (Canada). Front For Glob Change.

[91] Milota M, Puettmann M. Life cycle assessment for the production of southeastern softwood lumber. CORRIM Final Report. 2019.

[92] Puettmann M, Kaestner D, Taylor A. Life cycle assessment for the production of southeast softwood plywood. CORRIM Final Report. 2020.

[93] Puettmann M, Kaestner D, Taylor A. Life cycle assessment for the production of oriented strandboard production. CORRIM Final Report. 2020.

[94] Puettmann M, Salazar J. Cradle to gate life cycle assessment of North American particleboard production. CORRIM Final Report. 2018.

[95] Puettmann M, Salazar J. Cradle to gate life cycle assessment of North American medium density fiberboard production. CORRIM Final Report. 2019.

[96] Bergman RD, Alanya-Rosenbaum S. Cradle-to-gate life cycle assessment of laminated veneer lumber (LVL) Produced in the southeast region of the United States. CORRIM Final Report. 2017.

[97] CORRIM. Cradle to gate life cycle assessment of North American laminated strand lumber production. CORRIM Final Report. 2015.

[98] Puettmann M, Bergman R, Oneil E. Cradle-to-gate life cycle assessment of North American hardboard and engineered wood siding and trim production. CORRIM Final Report. Seattle, WA: University of Washington; 2016.

[99] Puettmann M, Oneil E, Johnson L. Cradle to gate life cycle assessment of glue-laminated timbers production from the southeast. CORRIM Final Report. 2013.

[100] Puettmann M, Bergman R, Oneil E. Cradle to gate life cycle assessment of North American cellulosic fiberboard production. CORRIM Final Report. 2016.

[101] Puettmann M, Sinha A, Ganguly I. Life cycle assessment of cross laminated timbers produced in Oregon. CORRIM Final Report. 2018.

[102] Griscom BW, Adams J, Ellis PW, Houghton RA, Lomax G, Miteva DA (2017). Natural climate solutions. Proc Natl Acad Sci.

[103] Galik CS, Baker JS, Daigneault A, Latta G (2022). Crediting temporary forest carbon: Retrospective and empirical perspectives on accounting options. Front For Glob Change.

