# Supplementary material for: Modeling wood product carbon flows in southern us pine plantations: implications for carbon storage
Source: Carbon Balance Manag. 2024 Feb 21;19:8. doi: 10.1186/s13021-024-00254-4 (PMC10882772; doi:10.1186/s13021-024-00254-4)
Supplement: Supplementary file 1 — Additional file 1: Text 1. Descriptions of secondary product categories used in LobWISE and sources. Table S1. General end use category by primary product. Table S2. Specific end use category by primary product and general end use. Table S3. Lifespans of single family housing parts. Table S4. Lifespans of multi-family housing parts. Table S5. Lifespans of other products. Table S6. Portion of transitioned product that is landfilled. Table S7. Portion of transitioned product that is downcycled as landscaping. Table S8. Portion of transitioned product that is downcycled as bedding. Table S9. Portion of transitioned product that is downcycled as posts and pilings. Table S10. Portion of transitioned product that is downcycled as structural panels. Table S11. Portion of transitioned product that is downcycled as non-structural panels. Table S12. Fossil fuel emission factors. Table S13. Landfill decay parameters. Table S14. Landfill methane parameters. Table S15. Waste deduction parameters. Table S16. Portion of primary product carbon used to produce engineered wood. Table S17. Portion of carbon in logs of each size class that is used as each log type (i.e., cull factors). Table S18. Portion of log carbon used in primary products and mill residue. Table S19. Mill residue use. Table S20. “Fiber product” mill residue use. Table S21. Specific gravity of loblolly pine wood. Table S22. Recycling parameters. Table S23. Primary product carbon usage in housing (structural vs. non-structural). Table S24. Portion of structural housing carbon in each application. Table S25. Portion of non-structural housing carbon in each application. Table S26. Carbon storage and pulse emissions from loblolly and shortleaf pine timber harvested in 2020. Table S27. Division of carbon from loblolly and shortleaf pine timber harvested in 2020 by primary product category. Table S28. Division of carbon from loblolly and shortleaf pine timber harvested in 2020 by secondary product category. Table S29. Tot [file 13021_2024_254_MOESM1_ESM.docx]

**Supplemental Material**

**Text 1. Secondary Product Use Category Descriptions**

New residential construction- All wood products used in the construction of housing built in year 0 for single family housing, multi-family housing, and manufactured housing. Does not include furniture but does include permanent furnishings. Sub-categories include floors, walls, roofs, foundations, decks, exterior doors, interior doors, garage doors, windows, moulding, shelving, cabinets, and countertops. Does not include outdoor fence posts. (1–3)

Residential upkeep - All wood products used in maintenance, repair, additions, alterations, and replacements done on single family housing, multi-family housing, and manufactured housing. (2)

New building construction - All wood products used in the construction of lodging, office, commercial, healthcare, educational, religious, public safety, amusement and recreation, and manufactured buildings built in year 0. (2)

Other new structures - All wood products used in the construction of transportation, communication, dams, power, highway and street, sewage and waste disposal, water supply, and conservation and development projects built in year 0. Does not include utility poles or marine pilings. (2)

Other construction (non-residential upkeep, etc.) - All wood products used in maintenance, repair, additions, alterations, and replacements done to non-residential buildings and structures, and any other wood products used in construction and not included elsewhere (2).

Pallets - Includes wooden shipping pallets.

Other shipping - Includes wood boxes, crates, hampers, baskets, dunnage, blocking, bracing, and all other wood products used in shipping and handling, excluding pallets. (2)

Poles - All wooden utility poles.

Posts and pilings - Marine pilings and fence posts. Consumption data for this category, as separate from “poles,” “other new structures,” or “miscellaneous,” were not available. This category was created separately from “poles” to account for the shorter life span of marine pilings vs. utility poles. It was created separately from “other new structures” to account for recycling of treated wood from utility poles and the shorter lifespans of these downcycled products.

Bedding - Wood chips, shavings, or sawdust used by animals, including livestock, poultry, horses, small pets, etc.

Landscaping - Includes bark and chip mulch and any other wood product or byproduct used in landscaping.

Corrugated Boxes - Includes linerboard kraft, which typically makes up the outer layer of corrugated boxes, and corrugating medium, which typically is used for the fluting or middle layer (4).

Sanitary Products - Includes commercial and retail bathroom tissue, facial tissue, and other tissue. Excludes food and kitchen related tissues such as napkins and paper towels. Also includes products manufactured with wood fluff, such as diapers, incontinence pads, and menstrual products (4).

Packaging Cartonboard - Includes virgin cartonboard products used for packaging a variety of grocery store and other retail items. This category is intended for cartonboard that is kept relatively clean and may therefore be recycled. It includes cartonboard products such as soda, beer, and other beverage carriers; boxes containing dry and frozen food, auto parts, toys, etc.; cigarette cartons; milk and juice cartons; etc. (4).

Disposable Food Related Products - Includes of variety of paper products used to package, carry, eat, or clean food and other contaminating products. This category is intended for items that are not typically recycled. It includes products such as paper bags; pet food and cement bags; fast food wrappers; microwavable popcorn bags; butter and meat wrappers; paper napkins and towels; paper plates, cups, and bowls; etc. (4).

Miscellaneous Paper Products - Includes cigarette filters, cellophane, rayon, newsprint, bristols, and any other pulp and paper products not included in previous categories (4).

Furniture - Includes household, commercial, and institutional furniture. (2)

Other manufacturing - Includes all manufactured items that are not furniture, such as musical instruments, sports equipment, toys, brooms and mops, caskets, etc. (2)

Miscellaneous - Wood products that do not fit into other categories, including craft and hobby projects, advertising structures, excelsior, mining supports, wood flour, non-structural wood used in non-residential construction, etc. (2)

**Tables S1-25. Parameters Used in LobWISE**

**Table S1.** General end use category by primary product

| **Primary Product** | **End Use Category** | **Portion** | **Source** |
| --- | --- | --- | --- |
| Lumber | New Residential | 0.317 | (2) |
|  | Residential Upkeep | 0.305 | (2) |
|  | New Non-residential | 0.067 | (2) |
|  | Shipping | 0.169 | (2) |
|  | Manufacturing | 0.08 | (2) |
|  | Other | 0.054 | (2) |
| Softwood Plywood | New Residential | 0.485 | (2) |
|  | Residential Upkeep | 0.232 | (2) |
|  | New Non-residential | 0.058 | (2) |
|  | Shipping | 0.059 | (2) |
|  | Manufacturing | 0.09 | (2) |
|  | Other | 0.077 | (2) |
| OSB | New Residential | 0.485 | (2) |
|  | Residential Upkeep | 0.232 | (2) |
|  | New Non-residential | 0.058 | (2) |
|  | Shipping | 0.059 | (2) |
|  | Manufacturing | 0.09 | (2) |
|  | Other | 0.077 | (2) |
| Non-Structural Panels | New Residential | 0.014 | (2) |
|  | Residential Upkeep | 0.216 | (2) |
|  | New Non-residential | 0.058 | (2) |
|  | Shipping | 0.03 | (2) |
|  | Manufacturing | 0.37 | (2) |
|  | Other | 0.314 | (2) |
| Engineered Wood | New Residential | 0.8 | Professional judgment |
|  | Residential Upkeep | 0.05 | Professional judgment |
|  | New Non-residential | 0.15 | Professional judgment |
|  | Shipping | 0 | Professional judgment |
|  | Manufacturing | 0 | Professional judgment |
|  | Other | 0 | Professional judgment |
| Poles and Pilings | Poles | 0.35 | (5) professional judgment |
|  | Posts and Pilings | 0.65 | (5) professional judgment |
|  | New Residential | 0 | Professional judgment |
|  | Residential Upkeep | 0 | Professional judgment |
|  | New Non-residential | 0 | Professional judgment |
|  | Shipping | 0 | Professional judgment |
|  | Manufacturing | 0 | Professional judgment |
|  | Other | 0 | Professional judgment |
| Paper and Paper Products |  |  |  |
| From Roundwood | Corrugated Boxes | 0.556 | (4) |
|  | Sanitary Products | 0.227 | (4) |
|  | Packaging Cartonboard | 0.120 | (4) |
|  | Disposable Food Related Products | 0.061 | (4) |
|  | Miscellaneous Paper Products | 0.036 | (4) |
| From Coarse Residue | Corrugated Boxes | 0.608 | (4) |
|  | Sanitary Products | 0.196 | (4) |
|  | Packaging Cartonboard | 0.099 | (4) |
|  | Disposable Food Related Products | 0.068 | (4) |
|  | Miscellaneous Paper Products | 0.029 | (4) |
| From Fine Residue | Corrugated Boxes | 0 | (4) |
|  | Sanitary Products | 0.498 | (4) |
|  | Packaging Cartonboard | 0 | (4) |
|  | Disposable Food Related Products | 0.502 | (4) |
|  | Miscellaneous Paper Products | 0 | (4) |

**Table S2.** Specific end use category by primary product and general end use

| **Primary Product** | **General End Use** | **Specific End Use** | **Portion** | **Source** |
| --- | --- | --- | --- | --- |
| Lumber | New Residential | Manufactured | 0.042 | (2) |
|  |  | Multi-family homes | 0.125 | (2) |
|  |  | Single family homes | 0.833 | (2) |
|  | Residential Upkeep | Manufactured | 0.042 | * |
|  |  | Multi-family homes | 0.125 | * |
|  |  | Single family homes | 0.833 | * |
|  | New Non-residential | Buildings | 0.627 | (2) |
|  |  | Other | 0.373 | (2) |
|  | Shipping | Pallets | 0.92 | (2) |
|  |  | Other | 0.08 | (2) |
|  | Manufacturing | Furniture | 0.284 | (2) |
|  |  | Other | 0.716 | (2) |
| Softwood Plywood | New Residential | Manufactured | 0.037 | (2) |
|  |  | Multi-family homes | 0.11 | (2) |
|  |  | Single family homes | 0.853 | (2) |
|  | Residential Upkeep | Manufactured | 0.037 | * |
|  |  | Multi-family homes | 0.11 | * |
|  |  | Single family homes | 0.853 | * |
|  | New Non-residential | Buildings | 0.882 | (2) |
|  |  | Other | 0.118 | (2) |
|  | Shipping | Pallets | 0 | Professional judgment |
|  |  | Other | 1 | Professional judgment |
|  | Manufacturing | Furniture | 0.225 | (2) |
|  |  | Other | 0.775 | (2) |
| OSB | New Residential | Manufactured | 0.037 | (2) |
|  |  | Multi-family homes | 0.11 | (2) |
|  |  | Single family homes | 0.853 | (2) |
|  | Residential Upkeep | Manufactured | 0.037 | * |
|  |  | Multi-family homes | 0.11 | * |
|  |  | Single family homes | 0.853 | * |
|  | New Non-residential | Buildings | 0.882 | (2) |
|  |  | Other | 0.118 | (2) |
|  | Shipping | Pallets | 0 | Professional judgment |
|  |  | Other | 1 | Professional judgment |
|  | Manufacturing | Furniture | 0.225 | (2) |
|  |  | Other | 0.775 | (2) |
| Non-Structural Panels | New Residential | Manufactured | 0.823 | (2) |
|  |  | Multi-family homes | 0.002 | (2) |
|  |  | Single family homes | 0.175 | (2) |
|  | Residential Upkeep | Manufactured | 0.823 | * |
|  |  | Multi-family homes | 0.002 | * |
|  |  | Single family homes | 0.175 | * |
|  | New Non-residential | Buildings | 0.006 | (2) |
|  |  | Other | 0.994 | (2) |
|  | Shipping | Pallets | 0 | Professional judgment |
|  |  | Other | 1 | Professional judgment |
|  | Manufacturing | Furniture | 0.438 | (2) |
|  |  | Other | 0.562 | (2) |
| Engineered Wood | New Residential | Manufactured | 0.05 | Professional judgment |
|  |  | Multi-family homes | 0.202 | (3) |
|  |  | Single family homes | 0.748 | (3) |
|  | Residential Upkeep | Manufactured | 0.05 | * |
|  |  | Multi-family homes | 0.202 | * |
|  |  | Single family homes | 0.748 | * |
|  | New Non-residential | Buildings | 1 | Professional judgment |
|  |  | Other | 0 | Professional judgment |
|  | Shipping | Pallets | 0 | Professional judgment |
|  |  | Other | 1 | Professional judgment |
|  | Manufacturing | Furniture | 0.5 | Professional judgment |
|  |  | Other | 0.5 | Professional judgment |
| Poles and Pilings | New Residential | Manufactured | 0 | Professional judgment |
|  |  | Multi-family homes | 0 | Professional judgment |
|  |  | Single family homes | 1 | Professional judgment |
|  | Residential Upkeep | Manufactured | 0 | * |
|  |  | Multi-family homes | 0 | * |
|  |  | Single family homes | 1 | * |
|  | New Non-residential | Buildings | 1 | Professional judgment |
|  |  | Other | 0 | Professional judgment |
|  | Shipping | Pallets | 0 | --- |
|  |  | Other | 1 | --- |
|  | Manufacturing | Furniture | 1 | Professional judgment |
|  |  | Other | 0 | Professional judgment |

*****Upkeep portions are assumed to be the same as new construction.

**Table S3.** Lifespans of single family housing parts

| **Housing Part** | **Material** | **Modal Estimate**  **(*mode*)** | **Scale Factor**  **(l)** | **Shape Factor**  **(*k*)** | **Source** |
| --- | --- | --- | --- | --- | --- |
| **Single Family Home (whole house)** | | **125** | **150** | **2.63** | (6) |
| Floors | Lumber | 112 | 135 | 2.63 | (6,7) professional judgment |
| Walls |  | 112 | 135 | 2.63 | (6,7) professional judgment |
| Roofs |  | 125 | 150 | 2.63 | (6,7) |
| Foundations |  | 125 | 150 | 2.63 | (6,7) |
| Decks |  | 13 | 16 | 2.63 | (5) |
| Exterior doors |  | 112 | 135 | 2.63 | (6,7) professional judgment |
| Interior doors |  | 112 | 135 | 2.63 | (6,7) professional judgment |
| Garage doors |  | 25 | 30 | 2.63 | (8) |
| Windows |  | 30 | 36 | 2.63 | (6,7) |
| Moulding |  | 112 | 135 | 2.63 | (6,7) professional judgment |
| Shelving |  | 100 | 120 | 2.63 | (6,7) professional judgment |
| Cabinets |  | 50 | 60 | 2.63 | (6,7) |
| Countertops |  | 100 | 120 | 2.63 | (6,7) professional judgment |
| Floors | Engineered Wood | 112 | 135 | 2.63 | (6,7) professional judgment |
| Walls |  | 112 | 135 | 2.63 | (6,7) professional judgment |
| Roofs |  | 125 | 150 | 2.63 | (6,7) |
| Foundations |  | 125 | 150 | 2.63 | (6,7) |
| Decks |  | 13 | 16 | 2.63 | (5) |
| Exterior doors |  | 112 | 135 | 2.63 | (6,7) professional judgment |
| Interior doors |  | 112 | 135 | 2.63 | (6,7) professional judgment |
| Garage doors |  | 25 | 30 | 2.63 | (8) |
| Windows |  | 30 | 36 | 2.63 | (6,7) |
| Moulding |  | 112 | 135 | 2.63 | (6,7) professional judgment |
| Shelving |  | 100 | 120 | 2.63 | (6,7) professional judgment |
| Cabinets |  | 50 | 60 | 2.63 | (6,7) |
| Countertops |  | 100 | 120 | 2.63 | (6,7) professional judgment |
| Floors | Softwood Plywood | 112 | 135 | 2.63 | (6,7) professional judgment |
| Walls |  | 112 | 135 | 2.63 | (6,7) professional judgment |
| Roofs |  | 125 | 150 | 2.63 | (6,7) |
| Foundations |  | 125 | 150 | 2.63 | (6,7) |
| Decks |  | 13 | 16 | 2.63 | (5) |
| Exterior doors |  | 112 | 135 | 2.63 | (6,7) professional judgment |
| Interior doors |  | 112 | 135 | 2.63 | (6,7) professional judgment |
| Garage doors |  | 25 | 30 | 2.63 | (8) |
| Windows |  | 30 | 36 | 2.63 | (6,7) |
| Moulding |  | 112 | 135 | 2.63 | (6,7) professional judgment |
| Shelving |  | 100 | 120 | 2.63 | (6,7) professional judgment |
| Cabinets |  | 40 | 48 | 2.63 | (6,7) professional judgment |
| Countertops |  | 100 | 120 | 2.63 | (6,7) professional judgment |
| Floors | OSB | 112 | 135 | 2.63 | (6,7) professional judgment |
| Walls |  | 112 | 135 | 2.63 | (6,7) professional judgment |
| Roofs |  | 125 | 150 | 2.63 | (6,7) |
| Foundations |  | 125 | 150 | 2.63 | (6,7) |
| Decks |  | 13 | 16 | 2.63 | (5) |
| Exterior doors |  | 112 | 135 | 2.63 | (6,7) professional judgment |
| Interior doors |  | 112 | 135 | 2.63 | (6,7) professional judgment |
| Garage doors |  | 25 | 30 | 2.63 | (8) |
| Windows |  | 30 | 36 | 2.63 | (6,7) |
| Moulding |  | 112 | 135 | 2.63 | (6,7) professional judgment |
| Shelving |  | 100 | 120 | 2.63 | (6,7) professional judgment |
| Cabinets |  | 40 | 48 | 2.63 | (6,7) professional judgment |
| Countertops |  | 100 | 120 | 2.63 | (6,7) professional judgment |
| Floors | Non-Structural Panels | 112 | 135 | 2.63 | (6,7) professional judgment |
| Walls |  | 112 | 135 | 2.63 | (6,7) professional judgment |
| Roofs |  | 125 | 150 | 2.63 | (6,7) |
| Foundations |  | 125 | 150 | 2.63 | (6,7) |
| Decks |  | 13 | 16 | 2.63 | (5) |
| Exterior doors |  | 112 | 135 | 2.63 | (6,7) professional judgment |
| Interior doors |  | 112 | 135 | 2.63 | (6,7) professional judgment |
| Garage doors |  | 25 | 30 | 2.63 | (8) |
| Windows |  | 30 | 36 | 2.63 | (6,7) |
| Moulding |  | 112 | 135 | 2.63 | (6,7) professional judgment |
| Shelving |  | 100 | 120 | 2.63 | (6,7) professional judgment |
| Cabinets |  | 40 | 48 | 2.63 | (6,7) professional judgment |
| Countertops |  | 100 | 120 | 2.63 | (6,7) professional judgment |
| Floors | Poles and Pilings | 125 | 150 | 2.63 | (6,7) professional judgment |
| Walls |  | 125 | 150 | 2.63 | (6,7) |
| Roofs |  | 125 | 150 | 2.63 | (6,7) |
| Foundations |  | 125 | 150 | 2.63 | (6,7) |
| Decks |  | 13 | 16 | 2.63 | (5) |
| Exterior doors |  | 125 | 150 | 2.63 | (6,7) |
| Interior doors |  | 125 | 150 | 2.63 | (6,7) |
| Garage doors |  | 25 | 30 | 2.63 | (8) |
| Windows |  | 30 | 36 | 2.63 | (6,7) |
| Moulding |  | 125 | 150 | 2.63 | (6,7) |
| Shelving |  | 100 | 120 | 2.63 | (6,7) professional judgment |
| Cabinets |  | 50 | 60 | 2.63 | (6,7) |
| Countertops |  | 100 | 120 | 2.63 | (6,7) professional judgment |

**Table S4.** Lifespans of multi-family housing parts

| **Housing Part** | **Material** | **Modal Estimate**  **(*mode*)** | **Scale Factor**  **(l)** | **Shape Factor**  **(*k*)** | **Source** |
| --- | --- | --- | --- | --- | --- |
| **Multi-Family Home (whole unit)** | | **110** | **132** | **2.63** | (6) professional judgment |
| Floors | Lumber | 99 | 119 | 2.63 | (6,7) professional judgment |
| Walls |  | 99 | 119 | 2.63 | (6,7) professional judgment |
| Roofs |  | 110 | 132 | 2.63 | (6,7) |
| Foundations |  | 110 | 132 | 2.63 | (6,7) |
| Decks |  | 13 | 16 | 2.63 | (5) |
| Exterior doors |  | 88 | 106 | 2.63 | (6,7) professional judgment |
| Interior doors |  | 88 | 106 | 2.63 | (6,7) professional judgment |
| Garage doors |  | 25 | 30 | 2.63 | (8) |
| Windows |  | 30 | 36 | 2.63 | (6,7) |
| Moulding |  | 88 | 106 | 2.63 | (6,7) professional judgment |
| Shelving |  | 77 | 92 | 2.63 | (6,7) professional judgment |
| Cabinets |  | 40 | 48 | 2.63 | (6,7) professional judgment |
| Countertops |  | 77 | 92 | 2.63 | (6,7) professional judgment |
| Floors | Engineered Wood | 99 | 119 | 2.63 | (6,7) professional judgment |
| Walls |  | 99 | 119 | 2.63 | (6,7) professional judgment |
| Roofs |  | 110 | 132 | 2.63 | (6,7) |
| Foundations |  | 110 | 132 | 2.63 | (6,7) |
| Decks |  | 13 | 16 | 2.63 | (5) |
| Exterior doors |  | 88 | 106 | 2.63 | (6,7) professional judgment |
| Interior doors |  | 88 | 106 | 2.63 | (6,7) professional judgment |
| Garage doors |  | 25 | 30 | 2.63 | (8) |
| Windows |  | 30 | 36 | 2.63 | (6,7) |
| Moulding |  | 88 | 106 | 2.63 | (6,7) professional judgment |
| Shelving |  | 77 | 92 | 2.63 | (6,7) professional judgment |
| Cabinets |  | 40 | 48 | 2.63 | (6,7) professional judgment |
| Countertops |  | 77 | 92 | 2.63 | (6,7) professional judgment |
| Floors | Softwood Plywood | 99 | 119 | 2.63 | (6,7) professional judgment |
| Walls |  | 99 | 119 | 2.63 | (6,7) professional judgment |
| Roofs |  | 110 | 132 | 2.63 | (6,7) |
| Foundations |  | 110 | 132 | 2.63 | (6,7) |
| Decks |  | 13 | 16 | 2.63 | (5) |
| Exterior doors |  | 88 | 106 | 2.63 | (6,7) professional judgment |
| Interior doors |  | 88 | 106 | 2.63 | (6,7) professional judgment |
| Garage doors |  | 25 | 30 | 2.63 | (8) |
| Windows |  | 30 | 36 | 2.63 | (6,7) |
| Moulding |  | 88 | 106 | 2.63 | (6,7) professional judgment |
| Shelving |  | 77 | 92 | 2.63 | (6,7) professional judgment |
| Cabinets |  | 30 | 36 | 2.63 | (6,7) professional judgment |
| Countertops |  | 77 | 92 | 2.63 | (6,7) professional judgment |
| Floors | OSB | 99 | 119 | 2.63 | (6,7) professional judgment |
| Walls |  | 99 | 119 | 2.63 | (6,7) professional judgment |
| Roofs |  | 110 | 132 | 2.63 | (6,7) |
| Foundations |  | 110 | 132 | 2.63 | (6,7) |
| Decks |  | 13 | 16 | 2.63 | (5) |
| Exterior doors |  | 88 | 106 | 2.63 | (6,7) professional judgment |
| Interior doors |  | 88 | 106 | 2.63 | (6,7) professional judgment |
| Garage doors |  | 25 | 30 | 2.63 | (8) |
| Windows |  | 30 | 36 | 2.63 | (6,7) |
| Moulding |  | 88 | 106 | 2.63 | (6,7) professional judgment |
| Shelving |  | 77 | 92 | 2.63 | (6,7) professional judgment |
| Cabinets |  | 30 | 36 | 2.63 | (6,7) professional judgment |
| Countertops |  | 77 | 92 | 2.63 | (6,7) professional judgment |
| Floors | Non-Structural Panels | 99 | 119 | 2.63 | (6,7) professional judgment |
| Walls |  | 99 | 119 | 2.63 | (6,7) professional judgment |
| Roofs |  | 110 | 132 | 2.63 | (6,7) |
| Foundations |  | 110 | 132 | 2.63 | (6,7) |
| Decks |  | 13 | 16 | 2.63 | (5) |
| Exterior doors |  | 88 | 106 | 2.63 | (6,7) professional judgment |
| Interior doors |  | 88 | 106 | 2.63 | (6,7) professional judgment |
| Garage doors |  | 25 | 30 | 2.63 | (8) |
| Windows |  | 30 | 36 | 2.63 | (6,7) |
| Moulding |  | 88 | 106 | 2.63 | (6,7) professional judgment |
| Shelving |  | 77 | 92 | 2.63 | (6,7) professional judgment |
| Cabinets |  | 30 | 36 | 2.63 | (6,7) professional judgment |
| Countertops |  | 77 | 92 | 2.63 | (6,7) professional judgment |
| Floors | Poles and Pilings | 110 | 132 | 2.63 | (6,7) |
| Walls |  | 110 | 132 | 2.63 | (6,7) |
| Roofs |  | 110 | 132 | 2.63 | (6,7) |
| Foundations |  | 110 | 132 | 2.63 | (6,7) |
| Decks |  | 13 | 16 | 2.63 | (5) |
| Exterior doors |  | 110 | 132 | 2.63 | (6,7) |
| Interior doors |  | 110 | 132 | 2.63 | (6,7) |
| Garage doors |  | 25 | 30 | 2.63 | (8) |
| Windows |  | 30 | 36 | 2.63 | (6,7) |
| Moulding |  | 110 | 132 | 2.63 | (6,7) |
| Shelving |  | 110 | 132 | 2.63 | (6,7) |
| Cabinets |  | 40 | 48 | 2.63 | (6,7) professional judgment |
| Countertops |  | 110 | 132 | 2.63 | (6,7) |

**Table S5.** Lifespans of other products

| **Housing Part** | **Modal Estimate**  **(*mode*)** | **Scale Factor (l)** | **Shape Factor**  **(*k*)** | **Source** |
| --- | --- | --- | --- | --- |
| Manufactured home | 70 | 84 | 2.63 | (6) professional judgment |
| Single family home upkeep | 37 | 45 | 2.63 | (6,9) |
| Multi-family home upkeep | 33 | 40 | 2.63 | (6,9) |
| Manufactured upkeep | 21 | 25 | 2.63 | (6,9) |
| Building | 70 | 84 | 2.63 | (6,10–12) |
| Other new structure | 60 | 72 | 2.63 | (6,13–15) |
| Other (non-residential upkeep, etc.) | 21 | 25 | 2.63 | (6,9–12) |
| Pallets | 1 | 1 | 2.63 | (6,16) |
| Other shipping | 2 | 2 | 2.63 | Professional judgment |
| Poles | 60 | 72 | 2.63 | (6,17) |
| Posts/pilings | 30 | 36 | 2.63 | (6,8,18) professional judgment |
| Bedding | 1 | 1 | 2.63 | Professional judgment |
| Landscaping | 2 | 2 | 2.63 | (6,19) |
| Corrugated boxes | 1 | 1 | 2.63 | (20) professional judgment |
| Sanitary products | 1 | 1 | 2.63 | (20) professional judgment |
| Packaging cartonboard | 1 | 1 | 2.63 | (20) professional judgment |
| Disposable food related products | 1 | 1 | 2.63 | (20,21) professional judgment |
| Miscellaneous paper products | 1 | 1 | 2.63 | (20) professional judgment |
| Furniture | 13 | 16 | 2.63 | (6,21) |
| Other manufacturing | 7 | 8 | 2.63 | (6,22–25) professional judgment |
| Miscellaneous | 4 | 5 | 2.63 | Professional judgment |

**Table S6.** Portion of transitioned product that is landfilled

| **Secondary Product** | **MSW Landfill** | **C&D Landfill** | **Source** |
| --- | --- | --- | --- |
| Single family homes | 0 | 0.722 | (20) |
| Multi-family homes | 0 | 0.722 | (20) |
| Manufactured home | 0 | 0.722 | (20) |
| Single family home upkeep | 0 | 0.722 | (20) |
| Multi-family home upkeep | 0 | 0.722 | (20) |
| Manufactured upkeep | 0 | 0.722 | (20) |
| Building | 0 | 0.722 | (20) |
| Other new structure | 0 | 0.722 | (20) |
| Other (non-residential upkeep, etc.) | 0 | 0.722 | (20) |
| Pallets | 0.007 | 0.003 | (26,27) |
| Other shipping | 0.632 | 0 | (28) |
| Poles | 0 | 0.32 | (17) |
| Posts/pilings | 0 | 0.7 | (18) |
| Bedding | 0.5 | 0 | Professional judgment |
| Landscaping | 0 | 0 | Professional judgment |
| Corrugated boxes | 0.028 | 0 | (29) |
| Sanitary products | 0.656 | 0 | (4,29,30) |
| Packaging cartonboard | 0.640 | 0 | (29) |
| Disposable food related products | 0.819 | 0 | (29) |
| Miscellaneous paper products | 0.578 | 0 | (29) |
| Furniture | 0.801 | 0 | (29) |
| Other manufacturing | 0.801 | 0 | (29) professional judgment |
| Miscellaneous | 0.801 | 0 | (29) professional judgment |

**Table S7.** Portion of transitioned product that is downcycled as landscaping

| **Secondary Product** | **Portion** | **Source** |
| --- | --- | --- |
| Single family homes | 0.061 | (20) |
| Multi-family homes | 0.061 | (20) |
| Manufactured home | 0.061 | (20) |
| Single family home upkeep | 0.061 | (20) |
| Multi-family home upkeep | 0.061 | (20) |
| Manufactured upkeep | 0.061 | (20) |
| Building | 0.061 | (20) |
| Other new structure | 0.061 | (20) |
| Other (non-residential upkeep, etc.) | 0.061 | (20) |
| Pallets | 0.109 | (27) |
| Other shipping | 0.138 | (27,28) professional judgment |
| Poles | 0.061 | * |
| Posts/pilings | -- | --- |
| Bedding | -- | --- |
| Landscaping | -- | --- |
| Corrugated boxes | 0 | Professional judgment |
| Sanitary products | 0 | Professional judgment |
| Packaging cartonboard | 0 | Professional judgment |
| Disposable food related products | 0 | Professional judgment |
| Miscellaneous paper products | 0 | Professional judgment |
| Furniture | -- | Professional judgment |
| Other manufacturing | -- | Professional judgment |
| Miscellaneous | 0 | Professional judgment |

*The rate of pole downcycling is assumed to be the same as the rate for C&D waste.

**Table S8.** Portion of transitioned product that is downcycled as bedding

| **Secondary Product** | **Portion** | **Source** |
| --- | --- | --- |
| Single family homes | 0 | --- |
| Multi-family homes | 0 | --- |
| Manufactured home | 0 | --- |
| Single family home upkeep | 0 | --- |
| Multi-family home upkeep | 0 | --- |
| Manufactured upkeep | 0 | --- |
| Building | 0 | --- |
| Other new structure | 0 | --- |
| Other (non-residential upkeep, etc.) | 0 | --- |
| Pallets | 0.012 | (27) |
| Other shipping | 0 | --- |
| Poles | 0 | --- |
| Posts/pilings | -- | --- |
| Bedding | -- | --- |
| Landscaping | -- | --- |
| Corrugated boxes | 0 | --- |
| Sanitary products | 0 | --- |
| Packaging cartonboard | 0 | --- |
| Disposable food related products | 0 | --- |
| Miscellaneous paper products | 0 | --- |
| Furniture | -- | --- |
| Other manufacturing | -- | --- |
| Miscellaneous | 0 | --- |

**Table S9.** Portion of transitioned product that is downcycled as posts and pilings

| **Secondary Product** | **Portion** | **Source** |
| --- | --- | --- |
| Single family homes | 0 | --- |
| Multi-family homes | 0 | --- |
| Manufactured home | 0 | --- |
| Single family home upkeep | 0 | --- |
| Multi-family home upkeep | 0 | --- |
| Manufactured upkeep | 0 | --- |
| Building | 0 | --- |
| Other new structure | 0 | --- |
| Other (non-residential upkeep, etc.) | 0 | --- |
| Pallets | 0 | --- |
| Other shipping | 0 | --- |
| Poles | 0.409 | (18,20) |
| Posts/pilings | -- | --- |
| Bedding | -- | --- |
| Landscaping | -- | --- |
| Corrugated boxes | 0 | --- |
| Sanitary products | 0 | --- |
| Packaging cartonboard | 0 | --- |
| Disposable food related products | 0 | --- |
| Miscellaneous paper products | 0 | --- |
| Furniture | -- | --- |
| Other manufacturing | -- | --- |
| Miscellaneous | 0 | --- |

**Table S10.** Portion of transitioned product that is downcycled as structural panels

| **Secondary Product** | **Portion** | **Source** |
| --- | --- | --- |
| Single family homes | 0 | Professional judgment |
| Multi-family homes | 0 | Professional judgment |
| Manufactured home | 0 | Professional judgment |
| Single family home upkeep | 0 | Professional judgment |
| Multi-family home upkeep | 0 | Professional judgment |
| Manufactured upkeep | 0 | Professional judgment |
| Building | 0 | Professional judgment |
| Other new structure | 0 | Professional judgment |
| Other (non-residential upkeep, etc.) | 0 | Professional judgment |
| Pallets | 0 | --- |
| Other shipping | 0 | --- |
| Poles | 0 | --- |
| Posts/pilings | -- | --- |
| Bedding | -- | --- |
| Landscaping | -- | --- |
| Corrugated boxes | 0 | --- |
| Sanitary products | 0 | --- |
| Packaging cartonboard | 0 | --- |
| Disposable food related products | 0 | --- |
| Miscellaneous paper products | 0 | --- |
| Furniture | -- | --- |
| Other manufacturing | -- | --- |
| Miscellaneous | 0 | --- |

**Table S11.** Portion of transitioned product that is downcycled as non-structural panels

| **Secondary Product** | **Portion** | **Source** |
| --- | --- | --- |
| Single family homes | 0.029 | (20) professional judgment |
| Multi-family homes | 0.029 | (20) professional judgment |
| Manufactured home | 0.029 | (20) professional judgment |
| Single family home upkeep | 0.029 | (20) professional judgment |
| Multi-family home upkeep | 0.029 | (20) professional judgment |
| Manufactured upkeep | 0.029 | (20) professional judgment |
| Building | 0.029 | (20) professional judgment |
| Other new structure | 0.029 | (20) professional judgment |
| Other (non-residential upkeep, etc.) | 0.029 | (20) professional judgment |
| Pallets | 0 | --- |
| Other shipping | 0 | --- |
| Poles | 0 | --- |
| Posts/pilings | -- | --- |
| Bedding | -- | --- |
| Landscaping | -- | --- |
| Corrugated boxes | 0 | --- |
| Sanitary products | 0 | --- |
| Packaging cartonboard | 0 | --- |
| Disposable food related products | 0 | --- |
| Miscellaneous paper products | 0 | --- |
| Furniture | -- | --- |
| Other manufacturing | -- | --- |
| Miscellaneous | 0 | --- |

**Table S12.** Fossil fuel emission factors

| **Primary Product** | **Procurement** | **Transportation** | **Production** | **Source** |
| --- | --- | --- | --- | --- |
| Lumber | 0.0186 | 0.0168 | 0.0882 | (31) |
| Plywood | 0.0715 | 0.0096 | 0.1906 | (32) |
| OSB | 0.0687 | 0.0060 | 0.1309 | (33) |
| Non-structural panels | 0.2007 | 0.0057 | 0.2360 | (34,35) |
| Engineered wood | --- | --- | 0.3902* | (36–41) |
| Poles | 0.0186 | 0.0168 | 0.0882 | ** |
| Paper and paper products | 0.0533 | 0.0484 | 0.4162 | (42) |
| Pellets | --- | --- | 0.0867*** | (43) |

Emission factors are in tCO_2_e emitted per tCO_2_e stored in final product; all fossil fuel emissions are assumed to be in CO_2_e in year 0; *emissions for engineered wood products included the procurement, transportation, and production emissions of the main primary product (*i.e.*, lumber, plywood, or OSB) plus the production emissions for engineered wood; **the emission factors for poles are assumed to be the same as the factors for sawtimber; ***a breakdown of emission factors for pellets by life stage was not available, but this factor includes procurement, transportation, and production emissions for pellets manufactured from roundwood; fossil fuel emissions for primary products manufactured from mill residue included the procurement and transportation emissions for the intended primary product and the production emissions for the actual primary product.

**Table S13.** Landfill decay parameters

| **Parameter** | **MSW Landfills** | **C&D Landfills** | **Source** |
| --- | --- | --- | --- |
| Fraction of paper carbon that will decay (DOC_f_) | 0.262 | 0.262 | (44) |
| Fraction of wood carbon that will decay (DOC_f_) | 0.029 | 0.029 | (45) |
| Half-life of decayable paper carbon (t_1/2_) | 15 | 15 | (44) |
| Half-life of decayable wood carbon (t_1/2_) | 30 | 30 | (44) |

**Table S14.** Landfill methane parameters

| **Parameter** | **MSW Landfills** | **C&D Landfills** | **Source** |
| --- | --- | --- | --- |
| F (portion of landfill gas that is CH_4_ by volume) | 0.5 | 0.5 | (46) |
| R (portion of generated CH_4_ that is recovered) | 0.6 | 0 | (47) |
| OX (portion of non-recovered CH_4_ that is oxidized) | 0.22 | 0.1 | (47) |
| Methane to CO_2_e conversion factor | 30 | 30 | (42) |

**Table S15.** Waste deduction parameters

| **Parameter** | **Portion** | **Source** |
| --- | --- | --- |
| Portion of carbon transitioned from housing pool at time of construction | 0.056 | (2,5,48) |
| Portion of carbon transitioned from other pools at time of manufacture (excludes poles, landscaping, and bedding) | 0.08 | (49) |

**Table S16.** Portion of primary product carbon used to produce engineered wood

| **Primary Product** | **Portion** | **Source** |
| --- | --- | --- |
| Lumber | 0.03 | (2) professional judgment |
| Softwood Plywood | 0.06 | (2) professional judgment |
| OSB | 0.1 | (2) professional judgment |

Engineered wood products include glued laminated timber (glulam), cross-laminated timber (CLT), nail-laminated timber (NLT), I-joists, laminated veneer lumber (LVL), parallel-strand lumber (PSL), laminated strand lumber (LSL), oriented strand lumber (OSL), OSB rim boards, and softwood plywood rim boards.

**Table S17.** Portion of carbon in logs of each size class that is used as each log type (*i.e.,* cull factors)

| **Log Type** | **Poletimber sized logs (13-22 cm DBH)** | **Sawtimber sized logs (23+ cm DBH)** | **Source** |
| --- | --- | --- | --- |
| Pulp Logs | 0.827 | 0.162 | (50) |
| Saw Logs | 0.042 | 0.692 | (50) |
| Veneer Logs | 0.046 | 0.086 | (50) |
| Pole Logs | 0.009 | 0.009 | (50) |
| Composite Logs | 0.044 | 0.028 | (50) |
| Bioenergy Logs | 0.032 | 0.022 | (50) |

DBH = diameter at breast height.

**Table S18.** Portion of log carbon used in primary products and mill residue

| **Log Type** | **Primary Product or Residue Type** | **Portion** | **Source** |
| --- | --- | --- | --- |
| Sawtimber logs |  |  |  |
| 23-32 cm DBH | Lumber | 0.35 | (31,51–53) professional judgment |
|  | Bark | 0.09 | (50) |
|  | Coarse residue | 0.31 | (50) |
|  | Fine residue | 0.25 | (50) |
| 33-47 cm DBH | Lumber | 0.40 | (31,51–53) professional judgment |
|  | Bark | 0.08 | (50) |
|  | Coarse residue | 0.29 | (50) |
|  | Fine residue | 0.23 | (50) |
| 48+ cm DBH | Lumber | 0.50 | (31,51–53) professional judgment |
|  | Bark | 0.07 | (50) |
|  | Coarse residue | 0.24 | (50) |
|  | Fine residue | 0.19 | (50) |
| Pulpwood logs | Pulp or paper | 0.89 | (50) |
|  | Bark | 0.11 | (50) |
| Veneer logs | Plywood | 0.41 | (50) |
|  | Bark | 0.09 | (50) |
|  | Coarse residue | 0.3 | (50) |
|  | Fine residue | 0.23 | (50) |
| Pole logs | Poles | 0.81 | (50) |
|  | Bark | 0.13 | (50) |
|  | Coarse residue | 0.06 | (50) |
| Bioenergy logs | Bioenergy products | 0.9 | (50) |
|  | Bark | 0.1 | (50) |
| Composite logs | OSB | 0.9 | (50) |
|  | Bark | 0.1 | (50) |

**Table S19.** Mill residue use

| **Log Type** | **Residue Type** | **Use** | **Portion** | **Source** |
| --- | --- | --- | --- | --- |
| Sawtimber logs | Bark | Landscaping | 0.48 | (50) |
|  |  | Miscellaneous | 0.02 | (50) |
|  |  | Fiber product | 0.01 | (50) |
|  |  | Pellets | 0.02 | (50) |
|  |  | Otherwise burned | 0.47 | (50) |
|  | Coarse residue | Landscaping | 0.01 | (50) |
|  |  | Miscellaneous | 0.095 | (50) |
|  |  | Fiber product | 0.79 | (50) |
|  |  | Animal bedding | 0.005 | (50) |
|  |  | Pellets | 0.02 | (50) |
|  |  | Otherwise burned | 0.08 | (50) |
|  | Fine residue | Landscaping | 0.01 | (50) |
|  |  | Miscellaneous | 0.03 | (50) |
|  |  | Fiber product | 0.11 | (50) |
|  |  | Animal bedding | 0.08 | (50) |
|  |  | Pellets | 0.19 | (50) |
|  |  | Otherwise burned | 0.58 | (50) |
| Pulpwood logs | Bark | Landscaping | 0.03 | (50) |
|  |  | Otherwise burned | 0.97 | (50) |
| Veneer logs | Bark | Landscaping | 0.08 | (50) |
|  |  | Otherwise burned | 0.92 | (50) |
|  | Coarse residue | Fiber product | 0.76 | (50) |
|  |  | Otherwise burned | 0.24 | (50) |
|  | Fine residue | Landscaping | 0.02 | (50) |
|  |  | Fiber product | 0.07 | (50) |
|  |  | Otherwise burned | 0.91 | (50) |
| Pole logs | Bark | Landscaping | 0.65 | (50) |
|  |  | Fiber product | 0.01 | (50) |
|  |  | Otherwise burned | 0.34 | (50) |
|  | Coarse residue | Landscaping | 0.15 | (50) |
|  |  | Miscellaneous | 0.08 | (50) |
|  |  | Fiber product | 0.5 | (50) |
|  |  | Otherwise burned | 0.27 | (50) |
| Bioenergy logs | Bark | Landscaping | 0.04 | (50) |
|  |  | Otherwise burned | 0.96 | (50) |
| Composite logs | Bark | Landscaping | 0.1 | (50) |
|  |  | Fiber product | 0.06 | (50) |
|  |  | Otherwise burned | 0.84 | (50) |

**Table S20.** “Fiber product” mill residue use

| **Residue Type** | **Use** | **Portion** | **Source** |
| --- | --- | --- | --- |
| Bark | Paper | 0.181 | (4) |
|  | Non-structural panels | 0.819 | (4) |
| Fine residue | Paper | 0.181 | (4) |
|  | Non-structural panels | 0.819 | (4) |
| Coarse residue | Paper | 0.954 | (4) |
|  | Non-structural panels | 0.046 | (4) |

**Table S21.** Specific gravity of loblolly pine wood

| **Parameter** | **Loblolly Pine** | **Source** |
| --- | --- | --- |
| Specific gravity | 0.47 | (31) |

**Table S22.** Recycling parameters

| **Product Category** | **Parameter** | **Value** | **Source** |
| --- | --- | --- | --- |
| Shipping products | Portion of pallets recycled as pallets | 0.7 | (27) |
|  | Number of times pallets are recycled | 3 | (27,54) |
| Pulp and paper products | Portion of corrugated boxes recycled as corrugated boxes | 0.914 | (55) |
|  | Number of times corrugated boxes are recycled | 5 | (56) |
|  | Portion of sanitary products recycled as sanitary products | 0 | (29) |
|  | Number of times sanitary products are recycled | 5 | (56) |
|  | Portion of packaging cartonboard recycled as packaging cartonboard | 0.208 | (29) |
|  | Number of packaging cartonboard is recycled | 5 | (56) |
|  | Portion of disposable food related products recycled as disposable food related products | 0 | (29) |
|  | Number of times disposable food related products are recycled | 5 | (56) |
|  | Portion of miscellaneous paper products recycled as miscellaneous paper products | 0.081 | (4,29) |
|  | Number of times miscellaneous paper is recycled | 5 | (56) |

**Table S23.** Primary product carbon usage in housing (structural vs. non-structural)

| **Housing Type** | **Primary Product** | **Structural** | **Non-Structural** | **Source** |
| --- | --- | --- | --- | --- |
| Single Family Home | Lumber | 0.926 | 0.074 | (1) |
|  | Engineered wood | 1 | 0 | Professional judgment |
|  | Structural panels | 0.992 | 0.008 | (1) |
|  | Non-structural panels | 0.57 | 0.43 | (1) |
|  | Poles and pilings | 1 | 0 | Professional judgment |
| Multi-Family Home | Lumber | 0.924 | 0.076 | (1) |
|  | Engineered wood | 1 | 0 | Professional judgment |
|  | Structural panels | 0.982 | 0.018 | (1) |
|  | Non-structural panels | 0.441 | 0.559 | (1) |
|  | Poles and pilings | 1 | 0 | Professional judgment |

**Table S24.** Portion of structural housing carbon in each application

| **Primary Product** | **Application** | **Single Family** | **Multi-Family** | **Source** |
| --- | --- | --- | --- | --- |
| Softwood lumber | Floors | 0.109 | 0.188 | (3) |
|  | Walls | 0.443 | 0.553 | (3) |
|  | Roofs | 0.351 | 0.254 | (3) |
|  | Foundations | 0.008 | 0.002 | (3) |
|  | Decks | 0.088 | 0.003 | (3) |
| Engineered wood | Floors | 0.862 | 0.906 | (3) |
|  | Walls | 0.089 | 0.059 | (3) |
|  | Roofs | 0.049 | 0.035 | (3) |
|  | Foundations | 0.000 | 0.000 | (3) |
|  | Decks | 0.000 | 0.000 | (3) |
| Softwood plywood | Floors | 0.339 | 0.453 | (3) |
|  | Walls | 0.203 | 0.118 | (3) |
|  | Roofs | 0.455 | 0.429 | (3) |
|  | Foundations | 0.003 | 0.000 | (3) |
|  | Decks | 0.000 | 0.000 | (3) |
| OSB | Floors | 0.236 | 0.427 | (3) |
|  | Walls | 0.340 | 0.153 | (3) |
|  | Roofs | 0.424 | 0.420 | (3) |
|  | Foundations | 0.000 | 0.000 | (3) |
|  | Decks | 0.000 | 0.000 | (3) |
| Non-structural panels | Floors | 0.918 | 0.563 | (3) |
|  | Walls | 0.082 | 0.438 | (3) |
|  | Roofs | 0.000 | 0.000 | (3) |
|  | Foundations | 0.000 | 0.000 | (3) |
|  | Decks | 0.000 | 0.000 | (3) |
| Poles and Pilings | Floors | 0.100 | 0.100 | Professional judgment |
|  | Walls | 0.600 | 0.600 | Professional judgment |
|  | Roofs | 0.100 | 0.100 | Professional judgment |
|  | Foundations | 0.100 | 0.100 | Professional judgment |
|  | Decks | 0.100 | 0.100 | Professional judgment |

**Table S25.** Portion of non-structural housing carbon in each application

| **Primary Product** | **Application** | **Single Family** | **Multi-Family** | **Source** |
| --- | --- | --- | --- | --- |
| Softwood lumber | Exterior doors | 0.059 | 0.045 | (1) |
|  | Interior doors | 0.397 | 0.338 | (1) |
|  | Garage doors | 0.046 | 0.030 | (1) |
|  | Windows | 0.100 | 0.030 | (1) |
|  | Moulding | 0.231 | 0.361 | (1) |
|  | Shelving | 0.037 | 0.023 | (1) |
|  | Cabinets | 0.130 | 0.173 | (1) |
|  | Countertops | 0.000 | 0.000 | (1) |
| Engineered wood | Exterior doors | 0.000 | 0.000 | (1) |
|  | Interior doors | 0.000 | 0.000 | (1) |
|  | Garage doors | 0.000 | 0.000 | (1) |
|  | Windows | 0.000 | 0.000 | (1) |
|  | Moulding | 0.000 | 0.000 | (1) |
|  | Shelving | 0.000 | 0.000 | (1) |
|  | Cabinets | 0.000 | 0.000 | (1) |
|  | Countertops | 0.000 | 0.000 | (1) |
| Structural panels  (OSB and softwood plywood) | Exterior doors | 0.034 | 0.042 | (1) |
|  | Interior doors | 0.171 | 0.208 | (1) |
|  | Garage doors | 0.078 | 0.042 | (1) |
|  | Windows | 0.000 | 0.000 | (1) |
|  | Moulding | 0.000 | 0.000 | (1) |
|  | Shelving | 0.410 | 0.458 | (1) |
|  | Cabinets | 0.000 | 0.000 | (1) |
|  | Countertops | 0.307 | 0.250 | (1) |
| Non-structural panels | Exterior doors | 0.096 | 0.030 | (1) |
|  | Interior doors | 0.212 | 0.077 | (1) |
|  | Garage doors | 0.072 | 0.012 | (1) |
|  | Windows | 0.000 | 0.000 | (1) |
|  | Moulding | 0.000 | 0.000 | (1) |
|  | Shelving | 0.108 | 0.098 | (1) |
|  | Cabinets | 0.256 | 0.692 | (1) |
|  | Countertops | 0.256 | 0.090 | (1) |
| Poles and Pilings | Exterior doors | 0.000 | 0.000 | Professional judgment |
|  | Interior doors | 0.000 | 0.000 | Professional judgment |
|  | Garage doors | 0.000 | 0.000 | Professional judgment |
|  | Windows | 0.300 | 0.300 | Professional judgment |
|  | Moulding | 0.700 | 0.700 | Professional judgment |
|  | Shelving | 0.000 | 0.000 | Professional judgment |
|  | Cabinets | 0.000 | 0.000 | Professional judgment |
|  | Countertops | 0.000 | 0.000 | Professional judgment |

**Tables S26-48. Results Tables**

**Table S26.** Carbon storage and pulse emissions from loblolly and shortleaf pine timber harvested in 2020

| **Years Since Harvest** | **Total Storage In Use** | **Total Storage In Landfills** | **CO_2_ Emissions from Products** | **CO_2_ Emissions from Landfills** | **CH_4_ Emissions from Landfills** |
| --- | --- | --- | --- | --- | --- |
|  | **MtC** | **MtC** | **MtCO_2_e** | **MtCO_2_e** | **MtCO_2_e** |
| 0 | 29.65 | 0.13 | -36.24 | 0.00 | 0.00 |
| 1 | 25.28 | 2.63 | -6.83 | 0.00 | 0.00 |
| 2 | 19.77 | 5.54 | -9.49 | -0.09 | -0.18 |
| 3 | 18.26 | 5.88 | -4.06 | -0.19 | -0.38 |
| 4 | 17.15 | 6.08 | -3.15 | -0.19 | -0.38 |
| 5 | 16.30 | 6.22 | -2.42 | -0.18 | -0.37 |
| 6 | 15.45 | 6.33 | -2.51 | -0.18 | -0.36 |
| 7 | 14.37 | 6.43 | -3.43 | -0.17 | -0.34 |
| 8 | 12.66 | 6.53 | -5.74 | -0.16 | -0.33 |
| 9 | 10.43 | 6.64 | -7.61 | -0.16 | -0.32 |
| 10 | 8.49 | 6.72 | -6.63 | -0.15 | -0.31 |
| 20 | 6.29 | 7.05 | -0.13 | -0.10 | -0.21 |
| 30 | 5.39 | 7.48 | -0.16 | -0.06 | -0.14 |
| 40 | 4.56 | 7.94 | -0.16 | -0.04 | -0.10 |
| 50 | 3.86 | 8.36 | -0.15 | -0.03 | -0.07 |
| 60 | 3.32 | 8.70 | -0.13 | -0.02 | -0.05 |
| 70 | 2.90 | 8.97 | -0.12 | -0.01 | -0.04 |
| 80 | 2.57 | 9.18 | -0.11 | -0.01 | -0.03 |
| 90 | 2.28 | 9.38 | -0.10 | -0.01 | -0.02 |
| 100 | 2.00 | 9.57 | -0.09 | 0.00 | -0.02 |
| 110 | 1.73 | 9.75 | -0.08 | 0.00 | -0.02 |
| 120 | 1.47 | 9.93 | -0.07 | 0.00 | -0.02 |

**Table S27.** Division of carbon from loblolly and shortleaf pine timber harvested in 2020 by primary product category

| **Primary Product** | **Carbon Division** |
| --- | --- |
| Lumber | 15.78% |
| Plywood | 2.52% |
| OSB | 2.68% |
| Non-structural panels | 1.53% |
| Engineered wood | 0.95% |
| Poles | 0.71% |
| Pulp and paper products | 47.58% |
| Landscaping | 2.30% |
| Bedding | 0.84% |
| Miscellaneous | 1.54% |
| Pellets | 7.72% |
| Otherwise Burned | 16.04% |

**Table S28.** Division of carbon from loblolly and shortleaf pine timber harvested in 2020 by secondary product category

| **Secondary Product** | **Carbon Division** | |
| --- | --- | --- |
| Single family homes | | 6.89% |
| Multi-family homes | | 1.06% |
| Manufactured home | | 0.34% |
| Single family home upkeep | | 4.84% |
| Multi-family home upkeep | | 0.70% |
| Manufactured upkeep | | 0.49% |
| Building | | 1.01% |
| Other new structure | | 0.49% |
| Other (non-residential upkeep, etc.) | | 1.64% |
| Pallets | | 2.26% |
| Other shipping | | 0.52% |
| Poles | | 0.25% |
| Posts/Pilings | | 0.42% |
| Bedding | | 0.84% |
| Landscaping | | 2.30% |
| Corrugated boxes | | 26.87% |
| Sanitary products | | 10.53% |
| Packaging cardboard | | 5.46% |
| Disposable food related products | | 3.07% |
| Miscellaneous paper products | | 1.65% |
| Furniture | | 0.66% |
| Other manufacturing | | 1.46% |
| Miscellaneous | | 1.42% |
| Total In Use | | 75.17% |

**Table S29.** Total carbon storage from 100 green tonnes of loblolly pine logs (tC)

| **Years Since Harvest** | **Pulp Logs** | **CNS Logs** | **Saw Logs** | **Large Saw Logs** | **Veneer Logs** | **Pole Logs** | **Composite Logs** | **Bioenergy Logs** |
| --- | --- | --- | --- | --- | --- | --- | --- | --- |
| 0 | 22.35 | 17.75 | 18.20 | 19.11 | 16.24 | 22.44 | 22.08 | 0.10 |
| 1 | 20.45 | 16.87 | 17.36 | 18.33 | 15.76 | 22.16 | 22.03 | 0.09 |
| 2 | 18.03 | 15.43 | 15.98 | 17.07 | 15.04 | 21.22 | 21.82 | 0.05 |
| 3 | 17.21 | 14.56 | 15.14 | 16.30 | 14.67 | 20.32 | 21.59 | 0.02 |
| 4 | 16.47 | 13.97 | 14.56 | 15.74 | 14.39 | 19.94 | 21.46 | 0.00 |
| 5 | 15.85 | 13.56 | 14.14 | 15.31 | 14.18 | 19.86 | 21.37 | 0.00 |
| 6 | 15.19 | 13.14 | 13.72 | 14.86 | 13.97 | 19.82 | 21.30 | 0.00 |
| 7 | 14.19 | 12.69 | 13.27 | 14.42 | 13.67 | 19.76 | 21.24 | 0.00 |
| 8 | 12.40 | 12.09 | 12.70 | 13.91 | 13.16 | 19.67 | 21.19 | 0.00 |
| 9 | 9.98 | 11.34 | 12.00 | 13.32 | 12.49 | 19.56 | 21.14 | 0.00 |
| 10 | 7.87 | 10.70 | 11.40 | 12.80 | 11.91 | 19.45 | 21.10 | 0.00 |
| 20 | 5.93 | 9.91 | 10.63 | 12.07 | 11.19 | 18.69 | 20.72 | 0.00 |
| 30 | 5.63 | 9.59 | 10.30 | 11.70 | 10.89 | 17.44 | 20.29 | 0.00 |
| 40 | 5.45 | 9.34 | 10.02 | 11.38 | 10.65 | 16.02 | 19.87 | 0.00 |
| 50 | 5.33 | 9.14 | 9.80 | 11.12 | 10.45 | 14.82 | 19.51 | 0.00 |
| 60 | 5.26 | 8.98 | 9.63 | 10.92 | 10.29 | 13.90 | 19.20 | 0.00 |
| 70 | 5.21 | 8.87 | 9.50 | 10.77 | 10.16 | 13.15 | 18.93 | 0.00 |
| 80 | 5.18 | 8.79 | 9.41 | 10.65 | 10.04 | 12.51 | 18.69 | 0.00 |
| 90 | 5.16 | 8.71 | 9.32 | 10.55 | 9.93 | 11.96 | 18.45 | 0.00 |
| 100 | 5.15 | 8.64 | 9.25 | 10.46 | 9.82 | 11.54 | 18.21 | 0.00 |
| 110 | 5.14 | 8.58 | 9.18 | 10.37 | 9.72 | 11.23 | 17.98 | 0.00 |
| 120 | 5.14 | 8.52 | 9.11 | 10.29 | 9.61 | 11.03 | 17.75 | 0.00 |

**Table S30.** Annual biogenic CO_2_ pulse emissions from 100 green tonnes of loblolly pine logs (tCO_2_e)

| **Years Since Harvest** | **Pulp Logs** | **CNS Logs** | **Saw Logs** | **Large Saw Logs** | **Veneer Logs** | **Pole Logs** | **Composite Logs** | **Bioenergy Logs** |
| --- | --- | --- | --- | --- | --- | --- | --- | --- |
| 0 | -9.79 | -26.65 | -24.99 | -21.67 | -32.18 | -9.45 | -10.74 | -91.43 |
| 1 | -6.96 | -3.21 | -3.09 | -2.85 | -1.78 | -1.03 | -0.26 | -0.03 |
| 2 | -8.85 | -5.31 | -5.09 | -4.65 | -2.66 | -3.42 | -0.82 | -0.13 |
| 3 | -2.96 | -3.20 | -3.08 | -2.85 | -1.38 | -3.32 | -0.88 | -0.14 |
| 4 | -2.68 | -2.17 | -2.14 | -2.08 | -1.03 | -1.37 | -0.53 | -0.05 |
| 5 | -2.23 | -1.54 | -1.57 | -1.62 | -0.78 | -0.31 | -0.37 | -0.01 |
| 6 | -2.37 | -1.53 | -1.56 | -1.64 | -0.79 | -0.17 | -0.33 | 0.00 |
| 7 | -3.63 | -1.67 | -1.66 | -1.64 | -1.11 | -0.22 | -0.27 | 0.00 |
| 8 | -6.53 | -2.23 | -2.12 | -1.90 | -1.86 | -0.34 | -0.23 | 0.00 |
| 9 | -8.82 | -2.74 | -2.57 | -2.21 | -2.47 | -0.44 | -0.22 | 0.00 |
| 10 | -7.73 | -2.38 | -2.22 | -1.91 | -2.17 | -0.43 | -0.21 | 0.00 |
| 20 | -0.12 | -0.17 | -0.19 | -0.21 | -0.16 | -0.58 | -0.24 | 0.00 |
| 30 | -0.07 | -0.18 | -0.20 | -0.23 | -0.18 | -1.14 | -0.31 | 0.00 |
| 40 | -0.05 | -0.17 | -0.18 | -0.22 | -0.16 | -1.74 | -0.30 | 0.00 |
| 50 | -0.03 | -0.14 | -0.15 | -0.18 | -0.14 | -2.36 | -0.27 | 0.00 |
| 60 | -0.02 | -0.10 | -0.12 | -0.14 | -0.11 | -2.95 | -0.23 | 0.00 |
| 70 | -0.01 | -0.08 | -0.09 | -0.11 | -0.09 | -3.38 | -0.20 | 0.00 |
| 80 | -0.01 | -0.06 | -0.07 | -0.08 | -0.08 | -3.51 | -0.18 | 0.00 |
| 90 | 0.00 | -0.05 | -0.06 | -0.07 | -0.08 | -3.31 | -0.18 | 0.00 |
| 100 | 0.00 | -0.05 | -0.05 | -0.07 | -0.08 | -2.84 | -0.18 | 0.00 |
| 110 | 0.00 | -0.04 | -0.05 | -0.06 | -0.08 | -2.23 | -0.18 | 0.00 |
| 120 | 0.00 | -0.04 | -0.05 | -0.06 | -0.08 | -1.59 | -0.17 | 0.00 |

**Table S31.** Annual biogenic CH_4_ pulse emissions from 100 green tonnes of loblolly pine logs (tCO_2_e)

| **Years Since Harvest** | **Pulp Logs** | **CNS Logs** | **Saw Logs** | **Large Saw Logs** | **Veneer Logs** | **Pole Logs** | **Composite Logs** | **Bioenergy Logs** |
| --- | --- | --- | --- | --- | --- | --- | --- | --- |
| 0 | 0.00 | 0.00 | 0.00 | 0.00 | 0.00 | 0.00 | 0.00 | 0.00 |
| 1 | 0.00 | 0.00 | 0.00 | 0.00 | 0.00 | 0.00 | -0.01 | 0.00 |
| 2 | -0.21 | -0.06 | -0.05 | -0.04 | -0.05 | -0.01 | -0.01 | 0.00 |
| 3 | -0.45 | -0.12 | -0.11 | -0.09 | -0.10 | -0.01 | -0.01 | 0.00 |
| 4 | -0.45 | -0.12 | -0.11 | -0.09 | -0.11 | -0.01 | -0.01 | 0.00 |
| 5 | -0.44 | -0.11 | -0.11 | -0.09 | -0.10 | -0.01 | -0.01 | 0.00 |
| 6 | -0.42 | -0.11 | -0.10 | -0.09 | -0.10 | -0.01 | -0.01 | 0.00 |
| 7 | -0.41 | -0.11 | -0.10 | -0.08 | -0.10 | -0.01 | -0.01 | 0.00 |
| 8 | -0.39 | -0.10 | -0.10 | -0.08 | -0.09 | -0.01 | -0.01 | 0.00 |
| 9 | -0.38 | -0.10 | -0.09 | -0.08 | -0.09 | -0.01 | -0.01 | 0.00 |
| 10 | -0.36 | -0.10 | -0.09 | -0.08 | -0.09 | -0.01 | -0.01 | 0.00 |
| 20 | -0.23 | -0.07 | -0.07 | -0.06 | -0.06 | -0.02 | -0.02 | 0.00 |
| 30 | -0.15 | -0.05 | -0.05 | -0.05 | -0.05 | -0.04 | -0.03 | 0.00 |
| 40 | -0.09 | -0.04 | -0.04 | -0.04 | -0.04 | -0.06 | -0.04 | 0.00 |
| 50 | -0.06 | -0.04 | -0.04 | -0.04 | -0.03 | -0.07 | -0.04 | 0.00 |
| 60 | -0.04 | -0.03 | -0.03 | -0.04 | -0.03 | -0.06 | -0.04 | 0.00 |
| 70 | -0.02 | -0.03 | -0.03 | -0.03 | -0.03 | -0.06 | -0.04 | 0.00 |
| 80 | -0.01 | -0.02 | -0.02 | -0.03 | -0.02 | -0.05 | -0.04 | 0.00 |
| 90 | -0.01 | -0.02 | -0.02 | -0.02 | -0.02 | -0.04 | -0.04 | 0.00 |
| 100 | -0.01 | -0.02 | -0.02 | -0.02 | -0.02 | -0.03 | -0.04 | 0.00 |
| 110 | 0.00 | -0.01 | -0.02 | -0.02 | -0.02 | -0.03 | -0.04 | 0.00 |
| 120 | 0.00 | -0.01 | -0.01 | -0.02 | -0.02 | -0.02 | -0.04 | 0.00 |

**Table S32.** Total biogenic and fossil fuel annual pulse emissions from 100 green tonnes of loblolly pine logs (tCO_2_e)

| **Years Since Harvest** | **Pulp Logs** | **CNS Logs** | **Saw Logs** | **Large Saw Logs** | **Veneer Logs** | **Pole Logs** | **Composite Logs** | **Bioenergy Logs** |
| --- | --- | --- | --- | --- | --- | --- | --- | --- |
| 0 | -52.07 | -44.23 | -42.17 | -38.05 | -54.70 | -20.10 | -31.18 | -98.59 |
| 1 | -6.96 | -3.21 | -3.09 | -2.85 | -1.78 | -1.03 | -0.26 | -0.03 |
| 2 | -9.07 | -5.36 | -5.14 | -4.69 | -2.72 | -3.43 | -0.83 | -0.13 |
| 3 | -3.41 | -3.31 | -3.19 | -2.94 | -1.49 | -3.33 | -0.89 | -0.14 |
| 4 | -3.12 | -2.28 | -2.25 | -2.17 | -1.14 | -1.38 | -0.54 | -0.05 |
| 5 | -2.67 | -1.66 | -1.67 | -1.71 | -0.88 | -0.33 | -0.38 | -0.01 |
| 6 | -2.80 | -1.64 | -1.67 | -1.73 | -0.89 | -0.18 | -0.34 | 0.00 |
| 7 | -4.03 | -1.78 | -1.76 | -1.72 | -1.20 | -0.23 | -0.28 | 0.00 |
| 8 | -6.92 | -2.33 | -2.22 | -1.99 | -1.96 | -0.35 | -0.24 | 0.00 |
| 9 | -9.20 | -2.84 | -2.66 | -2.29 | -2.56 | -0.46 | -0.23 | 0.00 |
| 10 | -8.09 | -2.48 | -2.32 | -1.99 | -2.26 | -0.45 | -0.22 | 0.00 |
| 20 | -0.35 | -0.25 | -0.25 | -0.27 | -0.22 | -0.60 | -0.26 | 0.00 |
| 30 | -0.22 | -0.24 | -0.25 | -0.28 | -0.23 | -1.18 | -0.34 | 0.00 |
| 40 | -0.14 | -0.21 | -0.23 | -0.26 | -0.20 | -1.80 | -0.34 | 0.00 |
| 50 | -0.09 | -0.17 | -0.19 | -0.22 | -0.17 | -2.42 | -0.31 | 0.00 |
| 60 | -0.06 | -0.13 | -0.15 | -0.18 | -0.14 | -3.01 | -0.28 | 0.00 |
| 70 | -0.03 | -0.10 | -0.11 | -0.14 | -0.12 | -3.43 | -0.24 | 0.00 |
| 80 | -0.02 | -0.08 | -0.09 | -0.11 | -0.11 | -3.56 | -0.22 | 0.00 |
| 90 | -0.01 | -0.07 | -0.08 | -0.10 | -0.10 | -3.35 | -0.22 | 0.00 |
| 100 | -0.01 | -0.06 | -0.07 | -0.09 | -0.10 | -2.88 | -0.22 | 0.00 |
| 110 | -0.01 | -0.06 | -0.07 | -0.08 | -0.10 | -2.25 | -0.21 | 0.00 |
| 120 | 0.00 | -0.06 | -0.06 | -0.08 | -0.09 | -1.62 | -0.21 | 0.00 |

**Table S33.** Portion of primary product carbon remaining in use up to 120 years after production

| **Years Since Harvest** | **Lumber** | **Plywood** | **OSB** | **Non-Structural Panels** | **Engineered Wood** | **Poles** | **Pulp and Paper Products** | **Landscaping and Bedding** | **Miscellaneous** |
| --- | --- | --- | --- | --- | --- | --- | --- | --- | --- |
| 0 | 0.932 | 0.944 | 0.944 | 0.936 | 0.948 | 0.948 | 1.000 | 1.000 | 0.920 |
| 1 | 0.913 | 0.939 | 0.939 | 0.933 | 0.948 | 0.948 | 0.789 | 0.806 | 0.905 |
| 2 | 0.882 | 0.920 | 0.920 | 0.920 | 0.947 | 0.948 | 0.539 | 0.400 | 0.832 |
| 3 | 0.858 | 0.898 | 0.898 | 0.900 | 0.946 | 0.947 | 0.493 | 0.121 | 0.688 |
| 4 | 0.834 | 0.884 | 0.884 | 0.879 | 0.945 | 0.946 | 0.458 | 0.016 | 0.495 |
| 5 | 0.807 | 0.874 | 0.874 | 0.854 | 0.945 | 0.945 | 0.431 | 0.001 | 0.302 |
| 6 | 0.778 | 0.863 | 0.863 | 0.823 | 0.944 | 0.942 | 0.402 | 0.000 | 0.152 |
| 7 | 0.754 | 0.852 | 0.852 | 0.788 | 0.944 | 0.940 | 0.358 | 0.000 | 0.062 |
| 8 | 0.737 | 0.841 | 0.841 | 0.752 | 0.944 | 0.936 | 0.277 | 0.000 | 0.020 |
| 9 | 0.725 | 0.830 | 0.830 | 0.715 | 0.943 | 0.932 | 0.166 | 0.000 | 0.005 |
| 10 | 0.714 | 0.821 | 0.821 | 0.679 | 0.942 | 0.927 | 0.069 | 0.000 | 0.001 |
| 20 | 0.628 | 0.747 | 0.747 | 0.396 | 0.930 | 0.826 | 0.000 | 0.000 | 0.000 |
| 30 | 0.539 | 0.668 | 0.668 | 0.191 | 0.904 | 0.652 | 0.000 | 0.000 | 0.000 |
| 40 | 0.452 | 0.591 | 0.591 | 0.093 | 0.866 | 0.468 | 0.000 | 0.000 | 0.000 |
| 50 | 0.375 | 0.524 | 0.524 | 0.060 | 0.818 | 0.334 | 0.000 | 0.000 | 0.000 |
| 60 | 0.314 | 0.468 | 0.468 | 0.044 | 0.761 | 0.253 | 0.000 | 0.000 | 0.000 |
| 70 | 0.271 | 0.423 | 0.422 | 0.031 | 0.698 | 0.200 | 0.000 | 0.000 | 0.000 |
| 80 | 0.238 | 0.383 | 0.382 | 0.022 | 0.630 | 0.156 | 0.000 | 0.000 | 0.000 |
| 90 | 0.210 | 0.345 | 0.344 | 0.014 | 0.559 | 0.116 | 0.000 | 0.000 | 0.000 |
| 100 | 0.184 | 0.307 | 0.306 | 0.009 | 0.487 | 0.082 | 0.000 | 0.000 | 0.000 |
| 110 | 0.159 | 0.270 | 0.269 | 0.005 | 0.417 | 0.055 | 0.000 | 0.000 | 0.000 |
| 120 | 0.136 | 0.233 | 0.232 | 0.003 | 0.351 | 0.035 | 0.000 | 0.000 | 0.000 |

Some values for year 0 are less than 1 because of construction and manufacturing waste deductions.

**Table S34.** Portion of primary product carbon remaining in landfills up to 120 years after production

| **Years Since Harvest** | **Lumber** | **Plywood** | **OSB** | **Non-Structural Panels** | **Engineered Wood** | **Poles** | **Pulp and Paper Products** | **Landscaping and Bedding** | **Miscellaneous** |
| --- | --- | --- | --- | --- | --- | --- | --- | --- | --- |
| 0 | 0.012 | 0.019 | 0.019 | 0.000 | 0.031 | 0.000 | 0.000 | 0.000 | 0.000 |
| 1 | 0.013 | 0.022 | 0.022 | 0.002 | 0.031 | 0.000 | 0.127 | 0.062 | 0.012 |
| 2 | 0.018 | 0.036 | 0.036 | 0.013 | 0.031 | 0.000 | 0.272 | 0.131 | 0.070 |
| 3 | 0.023 | 0.051 | 0.051 | 0.027 | 0.031 | 0.001 | 0.282 | 0.134 | 0.186 |
| 4 | 0.029 | 0.060 | 0.060 | 0.043 | 0.031 | 0.001 | 0.284 | 0.134 | 0.340 |
| 5 | 0.034 | 0.067 | 0.067 | 0.063 | 0.031 | 0.002 | 0.283 | 0.134 | 0.495 |
| 6 | 0.041 | 0.074 | 0.074 | 0.086 | 0.031 | 0.004 | 0.282 | 0.134 | 0.614 |
| 7 | 0.049 | 0.082 | 0.082 | 0.114 | 0.032 | 0.006 | 0.280 | 0.134 | 0.686 |
| 8 | 0.057 | 0.091 | 0.091 | 0.142 | 0.032 | 0.008 | 0.280 | 0.134 | 0.719 |
| 9 | 0.065 | 0.099 | 0.099 | 0.172 | 0.033 | 0.011 | 0.280 | 0.133 | 0.731 |
| 10 | 0.073 | 0.107 | 0.107 | 0.200 | 0.033 | 0.015 | 0.281 | 0.133 | 0.734 |
| 20 | 0.138 | 0.163 | 0.163 | 0.418 | 0.043 | 0.084 | 0.262 | 0.133 | 0.731 |
| 30 | 0.205 | 0.223 | 0.223 | 0.572 | 0.062 | 0.203 | 0.249 | 0.132 | 0.727 |
| 40 | 0.271 | 0.280 | 0.280 | 0.646 | 0.091 | 0.326 | 0.241 | 0.132 | 0.725 |
| 50 | 0.329 | 0.331 | 0.331 | 0.670 | 0.128 | 0.411 | 0.236 | 0.131 | 0.723 |
| 60 | 0.374 | 0.372 | 0.373 | 0.681 | 0.171 | 0.455 | 0.232 | 0.131 | 0.722 |
| 70 | 0.407 | 0.406 | 0.407 | 0.689 | 0.218 | 0.478 | 0.230 | 0.131 | 0.720 |
| 80 | 0.431 | 0.436 | 0.436 | 0.695 | 0.269 | 0.492 | 0.229 | 0.131 | 0.719 |
| 90 | 0.452 | 0.464 | 0.465 | 0.700 | 0.323 | 0.502 | 0.228 | 0.131 | 0.719 |
| 100 | 0.470 | 0.492 | 0.493 | 0.703 | 0.377 | 0.509 | 0.228 | 0.131 | 0.718 |
| 110 | 0.489 | 0.520 | 0.521 | 0.705 | 0.429 | 0.513 | 0.227 | 0.131 | 0.717 |
| 120 | 0.506 | 0.547 | 0.548 | 0.706 | 0.479 | 0.516 | 0.227 | 0.130 | 0.717 |

**Table S35.** Portion of primary product carbon remaining in use and in landfills up to 120 years after production

| **Years Since Harvest** | **Lumber** | **Plywood** | **OSB** | **Non-Structural Panels** | **Engineered Wood** | **Poles** | **Pulp and Paper Products** | **Landscaping and Bedding** | **Miscellaneous** |
| --- | --- | --- | --- | --- | --- | --- | --- | --- | --- |
| 0 | 0.944 | 0.962 | 0.962 | 0.936 | 0.978 | 0.948 | 1.000 | 1.000 | 0.920 |
| 1 | 0.926 | 0.961 | 0.961 | 0.936 | 0.977 | 0.948 | 0.916 | 0.868 | 0.917 |
| 2 | 0.899 | 0.955 | 0.955 | 0.932 | 0.976 | 0.948 | 0.810 | 0.531 | 0.903 |
| 3 | 0.881 | 0.949 | 0.949 | 0.928 | 0.975 | 0.948 | 0.775 | 0.255 | 0.874 |
| 4 | 0.861 | 0.944 | 0.944 | 0.922 | 0.975 | 0.947 | 0.742 | 0.150 | 0.835 |
| 5 | 0.841 | 0.940 | 0.940 | 0.916 | 0.975 | 0.947 | 0.714 | 0.135 | 0.797 |
| 6 | 0.819 | 0.936 | 0.936 | 0.909 | 0.975 | 0.946 | 0.684 | 0.134 | 0.766 |
| 7 | 0.802 | 0.933 | 0.933 | 0.902 | 0.975 | 0.945 | 0.638 | 0.134 | 0.748 |
| 8 | 0.794 | 0.931 | 0.931 | 0.894 | 0.975 | 0.944 | 0.557 | 0.134 | 0.739 |
| 9 | 0.789 | 0.929 | 0.929 | 0.886 | 0.975 | 0.943 | 0.446 | 0.133 | 0.736 |
| 10 | 0.786 | 0.927 | 0.927 | 0.878 | 0.975 | 0.941 | 0.350 | 0.133 | 0.735 |
| 20 | 0.764 | 0.909 | 0.909 | 0.811 | 0.973 | 0.907 | 0.262 | 0.133 | 0.731 |
| 30 | 0.742 | 0.888 | 0.888 | 0.759 | 0.966 | 0.846 | 0.249 | 0.132 | 0.727 |
| 40 | 0.720 | 0.869 | 0.869 | 0.736 | 0.956 | 0.776 | 0.241 | 0.132 | 0.725 |
| 50 | 0.701 | 0.853 | 0.853 | 0.729 | 0.944 | 0.717 | 0.236 | 0.131 | 0.723 |
| 60 | 0.687 | 0.839 | 0.839 | 0.724 | 0.930 | 0.672 | 0.232 | 0.131 | 0.722 |
| 70 | 0.676 | 0.828 | 0.828 | 0.720 | 0.915 | 0.635 | 0.230 | 0.131 | 0.720 |
| 80 | 0.668 | 0.818 | 0.818 | 0.716 | 0.898 | 0.603 | 0.229 | 0.131 | 0.719 |
| 90 | 0.661 | 0.808 | 0.808 | 0.714 | 0.880 | 0.576 | 0.228 | 0.131 | 0.719 |
| 100 | 0.654 | 0.798 | 0.798 | 0.712 | 0.862 | 0.555 | 0.228 | 0.131 | 0.718 |
| 110 | 0.647 | 0.789 | 0.788 | 0.710 | 0.845 | 0.540 | 0.227 | 0.131 | 0.717 |
| 120 | 0.641 | 0.779 | 0.779 | 0.709 | 0.828 | 0.530 | 0.227 | 0.130 | 0.717 |

Some values for year 0 are less than 1 because of construction and manufacturing waste deductions.

**Table S36.** Sensitivity of emissions from loblolly and shortleaf pine wood products to all tested parameters

| **Ranking** | **Parameter** | **Bounds** | **Units** | **µ** | **µ*** | **σ** |
| --- | --- | --- | --- | --- | --- | --- |
| 1 | DOC_f_ for paper in MSW landfills | 0.25 - 0.93 | portion | -16.68 | 16.68 | 4.49 |
| 2 | portion of CH_4_ recovered (R) in MSW landfills | 0.4019 - 0.8019 | portion | 9.62 | 9.62 | 4.71 |
| 3 | lifespan of corrugated boxes | 0.5 - 3 | years | 8.29 | 8.29 | 1.5 |
| 4 | downcycling to non-structural manufacturing of C&D waste | 0 - 0.129 | portion | -4.84 | 4.84 | 1.35 |
| 5 | production FF for pulp and paper | 0.3746 - 0.4579 | tCO2e emitted/tCO2e in product | -4.81 | 4.81 | 0.29 |
| 6 | portion of CH_4_ oxidized (OX) in MSW landfills | 0.0221 - 0.4221 | portion | 4.73 | 4.73 | 2.67 |
| 7 | average sawmill efficiency | 0.3 - 0.5 | portion | 4.45 | 4.45 | 1.11 |
| 8 | DOC_f_ for wood in C&D landfills | 0 - 0.5 | portion | -4.19 | 4.19 | 1.16 |
| 9 | recycling rate for corrugated boxes | 0.814 - 1 | portion | 3.98 | 3.98 | 2.12 |
| 10 | recycling times for corrugated boxes | 3 - 7 | times | 3.97 | 3.97 | 2.46 |
| 11 | lifespan of single family home upkeep | 12 - 62 | years | 2.77 | 2.77 | 1.1 |
| 12 | pulp mill efficiency | 0.79 - 0.99 | portion | -2.68 | 2.68 | 1.23 |
| 13 | construction waste | 0 - 0.156 | portion | -2.61 | 2.61 | 0.44 |
| 14 | DOC_f_ for wood in MSW landfills | 0 - 0.5 | portion | -2.49 | 2.49 | 0.71 |
| 15 | half-life of landfilled paper | 12 - 18 | years | 2.4 | 2.4 | 1.21 |
| 16 | landfill rate of sanitary products | 0.456 - 0.856 | portion | 0.94 | 1.48 | 1.51 |
| 17 | downcycling to non-structural manufacturing of pallets | 0 - 0.1 | portion | -1.37 | 1.37 | 0.4 |
| 18 | landfill rate of corrugated boxes | 0 - 0.128 | portion | 0.16 | 1.15 | 1.43 |
| 19 | portion of product used for engineered wood | 0 - +20 | portion, change in portion | -1.14 | 1.14 | 0.71 |
| 20 | landfill rate of C&D waste | 0.522 - 0.922 | portion | 1.06 | 1.11 | 1.02 |
| 21 | landfill rate of packaging cartonboard | 0.44 - 0.84 | portion | 0.24 | 1.01 | 1.21 |
| 22 | shape factor (*k*) for weibull distribution | 2.1 - 3.16 | -- | -0.95 | 0.95 | 0.51 |
| 23 | lifespan of sanitary products | 0.5 - 3 | years | 0.91 | 0.91 | 0.33 |
| 24 | lifespan of other construction | 7 - 35 | years | 0.9 | 0.9 | 0.4 |
| 25 | transportation FF for pulp and paper | 0.0435 - 0.0587 | tCO2e emitted/tCO2e in product | -0.86 | 0.86 | 0.05 |
| 26 | manufacturing waste | 0 - 0.28 | portion | -0.81 | 0.81 | 0.27 |
| 27 | veneer mill efficiency | 0.31 - 0.51 | portion | 0.76 | 0.76 | 0.15 |
| 28 | half-life of landfilled wood | 24 - 36 | years | 0.74 | 0.74 | 0.43 |
| 29 | landfill rate of furniture | 0.601 - 1 | portion | -0.73 | 0.73 | 0.43 |
| 30 | portion of coarse fiber res to composites | 0 - 0.146 | portion | 0.65 | 0.65 | 0.29 |
| 31 | procurement FF for pulp and paper | 0.048 - 0.0587 | tCO2e emitted/tCO2e in product | -0.61 | 0.61 | 0.04 |
| 32 | portion of CH_4_ oxidized (OX) in C&D landfills | 0 - 0.3 | portion | 0.6 | 0.6 | 0.49 |
| 33 | downcycling to bedding rate of C&D waste | 0 - 0.1 | portion | -0.58 | 0.58 | 0.42 |
| 34 | lifespan of packaging cartonboard | 0.5 - 3 | years | 0.58 | 0.58 | 0.25 |
| 35 | lifespan of furniture | 10.4 - 15.6 | years | -0.56 | 0.56 | 0.39 |
| 36 | lifespan of pallets | 0.5 - 3 | years | 0.5 | 0.5 | 0.15 |
| 37 | landfill rate of disposable food paper | 0.619 - 1 | portion | 0.05 | 0.48 | 0.62 |
| 38 | lifespan of bedding | 0.5 - 3 | years | -0.48 | 0.48 | 0.3 |
| 39 | portion of CH_4_ recovered (R) in C&D landfills | 0 - 0.2 | portion | 0.46 | 0.46 | 0.3 |
| 40 | lifespan of other manufacturing | 5.6 - 8.4 | years | -0.44 | 0.44 | 0.28 |
| 41 | landfill rate of miscellaneous | 0.601 - 1 | portion | 0.42 | 0.42 | 0.14 |
| 42 | lifespan of single family home | 100 - 150 | years | 0.41 | 0.41 | 0.23 |
| 43 | lifespan of multi-family home upkeep | 11 - 55 | years | 0.39 | 0.39 | 0.21 |
| 44 | landfill rate of other manufacturing | 0.601 - 1 | portion | -0.34 | 0.39 | 0.36 |
| 45 | composite mill efficiency | 0.8 - 1 | portion | 0.35 | 0.35 | 0.08 |
| 46 | production FF for lumber | 0.0794 - 0.097 | tCO2e emitted/tCO2e in product | -0.35 | 0.35 | 0.06 |
| 47 | downcycling to non-structural manufacturing of other shipping | 0 - 0.1 | portion | -0.34 | 0.34 | 0.07 |
| 48 | downcycling to posts/pilings of poles | 0.209 - 0.609 | portion | -0.31 | 0.31 | 0.1 |
| 49 | lifespan of manufactured home upkeep | 7 - 35 | years | 0.29 | 0.29 | 0.14 |
| 50 | landfill rate of landscaping | 0 - 0.2 | portion | 0 | 0.27 | 0.33 |
| 51 | lifespan of disposable food paper | 0.5 - 3 | years | 0.26 | 0.26 | 0.09 |
| 52 | recycling times for pallets | 1 - 5 | times | 0.25 | 0.25 | 0.16 |
| 53 | landfill rate of miscellaneous paper | 0.378 - 0.778 | portion | 0.1 | 0.25 | 0.29 |
| 54 | lifespan of poles | 48 - 72 | years | 0.24 | 0.24 | 0.1 |
| 55 | recycling rate for packaging cartonboard | 0.008 - 0.408 | portion | 0.24 | 0.24 | 0.11 |
| 56 | landfill rate of bedding | 0.3 - 0.7 | portion | 0.01 | 0.24 | 0.29 |
| 57 | lifespan of landscaping | 1 - 4 | years | 0.01 | 0.21 | 0.25 |
| 58 | downcycling to bedding rate of pallets | 0 - 0.112 | portion | -0.2 | 0.2 | 0.16 |
| 59 | portion of fine fiber res to composites | 0.619 - 1 | portion | 0.18 | 0.2 | 0.16 |
| 60 | downcycling to landscaping rate of C&D waste | 0 - 0.161 | portion | -0.13 | 0.17 | 0.21 |
| 61 | production FF for pellets | 0.0781 - 0.0954 | tCO2e emitted/tCO2e in product | -0.16 | 0.16 | 0.01 |
| 62 | production FF for engineered wood | 0.3511 - 0.4292 | tCO2e emitted/tCO2e in product | -0.16 | 0.16 | 0.19 |
| 63 | lifespan of miscellaneous paper | 0.5 - 3 | years | 0.16 | 0.16 | 0.04 |
| 64 | landfill rate of other shipping | 0.432 - 0.832 | portion | 0.15 | 0.15 | 0.05 |
| 65 | landfill rate of posts/pilings | 0.5 - 0.9 | portion | -0.15 | 0.15 | 0.09 |
| 66 | lifespan of buildings | 56 - 84 | years | 0.14 | 0.14 | 0.07 |
| 67 | landfill rate of pallets | 0 - 0.11 | portion | 0.13 | 0.13 | 0.06 |
| 68 | bioenergy mill efficiency | 0.8 - 1 | portion | -0.13 | 0.13 | 0.01 |
| 69 | recycling rate for pallets | 0.6 - 0.8 | portion | 0.13 | 0.13 | 0.09 |
| 70 | production FF for plywood | 0.1716 - 0.2097 | tCO2e emitted/tCO2e in product | -0.13 | 0.13 | 0.02 |
| 71 | production FF for non-structural panels | 0.2124 - 0.2596 | tCO2e emitted/tCO2e in product | -0.1 | 0.1 | 0.03 |
| 72 | lifespan of multi-family home | 88 - 132 | years | 0.09 | 0.09 | 0.05 |
| 73 | production FF for OSB | 0.1178 - 0.144 | tCO2e emitted/tCO2e in product | -0.09 | 0.09 | 0.01 |
| 74 | recycling rate for sanitary products | 0 - 0.1 | portion | 0.09 | 0.09 | 0.05 |
| 75 | lifespan of miscellaneous | 2 - 6 | years | 0.09 | 0.09 | 0.04 |
| 76 | procurement FF for non-structural panels | 0.1806 - 0.2207 | tCO2e emitted/tCO2e in product | -0.08 | 0.08 | 0.02 |
| 77 | downcycling to landscaping rate of pallets | 0.009 - 0.209 | portion | -0.07 | 0.08 | 0.09 |
| 78 | procurement FF for lumber | 0.0167 - 0.0204 | tCO2e emitted/tCO2e in product | -0.07 | 0.07 | 0.01 |
| 79 | transportation FF for lumber | 0.0151 - 0.0184 | tCO2e emitted/tCO2e in product | -0.06 | 0.06 | 0.01 |
| 80 | lifespan of other new structures | 48 - 72 | years | 0.06 | 0.06 | 0.03 |
| 81 | downcycling to bedding rate of other shipping | 0 - 0.1 | portion | -0.05 | 0.05 | 0.03 |
| 82 | lifespan of other shipping | 1 - 4 | years | 0.05 | 0.05 | 0.02 |
| 83 | procurement FF for OSB | 0.0618 - 0.0755 | tCO2e emitted/tCO2e in product | -0.05 | 0.05 | 0 |
| 84 | lifespan of single family home parts unconnected | -0.4 | % change in years | 0.05 | 0.05 | 0.02 |
| 85 | procurement FF for plywood | 0.0644 - 0.0787 | tCO2e emitted/tCO2e in product | -0.05 | 0.05 | 0.01 |
| 86 | pole mill efficiency | 0.71 - 0.91 | portion | 0.02 | 0.05 | 0.05 |
| 87 | recycling rate for miscellaneous paper | 0 - 0.281 | portion | 0.04 | 0.04 | 0.02 |
| 88 | downcycling to landscaping rate of other shipping | 0 - 0.338 | portion | -0.04 | 0.04 | 0.04 |
| 89 | lifespan of manufactured home | 56 - 84 | years | 0.04 | 0.04 | 0.02 |
| 90 | downcycling to non-structural manufacturing of poles | 0 - 0.1 | portion | -0.04 | 0.04 | 0.01 |
| 91 | lifespan of posts/pilings | 24 - 36 | years | -0.03 | 0.04 | 0.04 |
| 92 | production FF for poles | 0.0705 - 0.1058 | tCO2e emitted/tCO2e in product | -0.03 | 0.03 | 0 |
| 93 | recycling rate for disposable food paper | 0 - 0.1 | portion | 0.02 | 0.02 | 0.01 |
| 94 | landfill rate of poles | 0.12 - 0.52 | portion | 0.01 | 0.01 | 0.01 |
| 95 | portion of bark fiber res to composites | 0.619 - 1 | portion | 0.01 | 0.01 | 0.01 |
| 96 | procurement FF for poles | 0.0149 - 0.0223 | tCO2e emitted/tCO2e in product | -0.01 | 0.01 | 0 |
| 97 | transportation FF for plywood | 0.0086 - 0.0105 | tCO2e emitted/tCO2e in product | -0.01 | 0.01 | 0 |
| 98 | transportation FF for poles | 0.0134 - 0.0201 | tCO2e emitted/tCO2e in product | -0.01 | 0.01 | 0 |
| 99 | downcycling to bedding rate of poles | 0 - 0.1 | portion | 0 | 0 | 0 |
| 100 | transportation FF for OSB | 0.0054 - 0.0066 | tCO2e emitted/tCO2e in product | 0 | 0 | 0 |
| 101 | downcycling to landscaping rate of poles | 0 - 0.261 | portion | 0 | 0 | 0 |
| 102 | transportation FF for non-structural panels | 0.0051 - 0.0062 | tCO2e emitted/tCO2e in product | 0 | 0 | 0 |
| 103 | recycling times for packaging cartonboard | 3 - 7 | times | 0 | 0 | 0 |
| 104 | lifespan of multi-family home parts unconnected | -0.4 | % change in years | 0 | 0 | 0 |
| 105 | recycling times for miscellaneous paper | 3 - 7 | times | 0 | 0 | 0 |
| 106 | recycling times for sanitary products | 3 - 7 | times | 0 | 0 | 0 |
| 107 | recycling times for disposable food paper | 3 - 7 | times | 0 | 0 | 0 |

DOC_f_ = the fraction of landfilled organic carbon that will decay over time; fiber res = mill residue that is used for a fiber product (pulp and paper or non-structural panels); MSW = municipal solid waste; C&D = construction and demolition; average sawmill efficiency = sawmill efficiency for 33-47 cm saw logs, with smaller logs having -0.05 efficiency and larger logs having +0.1 efficiency; FF = fossil fuel emission rate; the magnitude of µ* indicates the influence of the parameter; the sign of µ indicates the direction of the effect; σ indicates interaction of the parameter with other parameters; the bounds indicate the tested range of parameter values (see section 2.3 of main text); the model output of which sensitivity was evaluated was the total (biogenic + fossil fuel) annual emission pulses from southwide loblolly and shortleaf pine harvested wood products produced over 120 years, discounted at 3%.

**Table S37.** Carbon storage from four silvicultural management scenarios, 60 years since initial planting (tC ha^-1^)

| **Silvicultural Regime** | ***In Situ* Carbon Storage** | ***Ex Situ* Carbon Storage** | **Total Carbon Storage** |
| --- | --- | --- | --- |
| Pulp-20 | 83.85 | 10.91 | 94.76 |
| Pulp-24 | 114.2 | 20.61 | 134.81 |
| ST-20 | 90.95 | 12.07 | 103.02 |
| ST-24 | 105.45 | 16.88 | 122.33 |

**Table S38.** Carbon storage from four silvicultural management scenarios, 120 years since initial planting (tC ha^-1^)

| **Silvicultural Regime** | ***In Situ* Carbon Storage** | ***Ex Situ* Carbon Storage** | **Total Carbon Storage** |
| --- | --- | --- | --- |
| Pulp-20 | 83.85 | 29.76 | 113.61 |
| Pulp-24 | 114.20 | 54.05 | 168.25 |
| ST-20 | 90.95 | 36.46 | 127.41 |
| ST-24 | 105.45 | 49.31 | 154.76 |

**Table S39.** Total carbon that enters the market or is burned by primary product for four silvicultural management scenarios (tC ha^-1^)

| **Primary Product** | **Pulp-20** | **Pulp-24** | **ST-20** | **ST-24** |
| --- | --- | --- | --- | --- |
| Lumber | 28.5 | 56.9 | 43.2 | 58.2 |
| Plywood | 7.1 | 11.4 | 8.0 | 10.4 |
| OSB | 9.5 | 12.9 | 8.5 | 11.0 |
| Non-structural panels | 3.4 | 6.5 | 4.8 | 6.3 |
| Engineered Wood | 2.4 | 3.9 | 2.8 | 3.7 |
| Poles | 2.3 | 3.4 | 2.3 | 3.0 |
| Pulp and paper products | 183.9 | 236.9 | 152.8 | 196.0 |
| Landscaping | 5.4 | 9.9 | 7.1 | 9.3 |
| Bedding | 1.8 | 3.6 | 2.6 | 3.4 |
| Miscellaneous | 3.3 | 6.5 | 4.8 | 6.3 |
| Pellets | -12.4 | -19.9 | -13.9 | -18.1 |
| Other burned | -45.5 | -71.5 | -49.5 | -64.3 |
| Total | 189.5 | 260.4 | 173.5 | 225.2 |

Includes six harvests for pulp regimes and four thins and final harvests for sawtimber regimes. Negative values indicate carbon (tC/ha) that was burned and, thus, emitted to the atmosphere.

**Table S40.** Total carbon that enters the market by secondary product category for four silvicultural management scenarios (tC ha^-1^)

| **Primary Product** | **Pulp-20** | **Pulp-24** | **ST-20** | **ST-24** |
| --- | --- | --- | --- | --- |
| New residential construction | 19.0 | 32.9 | 23.9 | 31.8 |
| Residential upkeep | 12.6 | 23.2 | 17.2 | 22.9 |
| Non-residential construction | 6.9 | 12.4 | 9.1 | 12.1 |
| Shipping | 5.4 | 10.3 | 7.7 | 10.4 |
| Poles, posts, and pilings | 2.2 | 3.2 | 2.2 | 2.8 |
| Bedding and landscaping | 7.1 | 13.4 | 9.8 | 12.8 |
| Corrugated boxes | 103.1 | 133.5 | 86.3 | 110.7 |
| Sanitary Products | 41.1 | 52.6 | 33.8 | 43.4 |
| Packaging cartonboard | 21.5 | 27.4 | 17.6 | 22.5 |
| Other pulp and paper | 18.1 | 23.4 | 15.1 | 19.4 |
| Manufacturing | 4.6 | 8.4 | 6.2 | 8.2 |
| Miscellaneous | 3.0 | 6.0 | 4.4 | 5.8 |

Includes six harvests for pulp regimes and four thins and final harvests for sawtimber regimes.

**Table S41.** Carbon storage in use from three wood usage scenarios and a business as usual (BAU) scenario (MtC)

| **Years Since Harvest** | **BAU** | **> CNS** | **> Lifespans** | **> Bioenergy** |
| --- | --- | --- | --- | --- |
| 0 | 29.65 | 29.49 | 29.66 | 23.47 |
| 1 | 25.28 | 25.09 | 26.16 | 20.37 |
| 2 | 19.77 | 19.52 | 21.56 | 16.36 |
| 3 | 18.26 | 18.00 | 20.24 | 15.15 |
| 4 | 17.15 | 16.88 | 19.28 | 14.26 |
| 5 | 16.30 | 16.02 | 18.61 | 13.58 |
| 6 | 15.45 | 15.17 | 18.01 | 12.90 |
| 7 | 14.37 | 14.08 | 17.49 | 12.08 |
| 8 | 12.66 | 12.35 | 17.01 | 10.84 |
| 9 | 10.43 | 10.10 | 16.56 | 9.26 |
| 10 | 8.49 | 8.14 | 16.12 | 7.88 |
| 20 | 6.29 | 5.97 | 6.51 | 6.11 |
| 30 | 5.39 | 5.14 | 5.71 | 5.23 |
| 40 | 4.56 | 4.35 | 4.93 | 4.43 |
| 50 | 3.86 | 3.68 | 4.24 | 3.75 |
| 60 | 3.32 | 3.16 | 3.66 | 3.22 |
| 70 | 2.90 | 2.77 | 3.20 | 2.82 |
| 80 | 2.57 | 2.46 | 2.85 | 2.50 |
| 90 | 2.28 | 2.17 | 2.55 | 2.21 |
| 100 | 2.00 | 1.91 | 2.28 | 1.94 |
| 110 | 1.73 | 1.65 | 2.02 | 1.68 |
| 120 | 1.47 | 1.41 | 1.78 | 1.44 |

CNS = chip-n-saw; reference inputs for the scenarios were loblolly and shortleaf pine timber harvested in the southern US in 2020.

**Table S42.** Carbon storage in landfills from three wood usage scenarios and a business as usual (BAU) scenario (MtC)

| **Years Since Harvest** | **BAU** | **> CNS** | **> Lifespans** | **> Bioenergy** |
| --- | --- | --- | --- | --- |
| 0 | 0.13 | 0.12 | 0.11 | 0.12 |
| 1 | 2.63 | 2.65 | 2.03 | 1.87 |
| 2 | 5.54 | 5.58 | 4.30 | 3.92 |
| 3 | 5.88 | 5.93 | 4.61 | 4.19 |
| 4 | 6.08 | 6.14 | 4.82 | 4.37 |
| 5 | 6.22 | 6.28 | 4.97 | 4.51 |
| 6 | 6.33 | 6.40 | 5.11 | 4.63 |
| 7 | 6.43 | 6.51 | 5.22 | 4.73 |
| 8 | 6.53 | 6.61 | 5.31 | 4.83 |
| 9 | 6.64 | 6.72 | 5.39 | 4.93 |
| 10 | 6.72 | 6.81 | 5.45 | 5.01 |
| 20 | 7.05 | 7.09 | 6.02 | 5.43 |
| 30 | 7.48 | 7.47 | 6.42 | 5.91 |
| 40 | 7.94 | 7.91 | 6.91 | 6.40 |
| 50 | 8.36 | 8.30 | 7.39 | 6.84 |
| 60 | 8.70 | 8.62 | 7.80 | 7.19 |
| 70 | 8.97 | 8.87 | 8.14 | 7.45 |
| 80 | 9.18 | 9.07 | 8.41 | 7.67 |
| 90 | 9.38 | 9.26 | 8.63 | 7.86 |
| 100 | 9.57 | 9.44 | 8.84 | 8.05 |
| 110 | 9.75 | 9.61 | 9.03 | 8.23 |
| 120 | 9.93 | 9.78 | 9.22 | 8.41 |

CNS = chip-n-saw; reference inputs for the scenarios were loblolly and shortleaf pine timber harvested in the southern US in 2020.

**Table S43.** Total carbon storage from three wood usage scenarios and a business as usual (BAU) scenario (MtC)

| **Years Since Harvest** | **BAU** | **> CNS** | **> Lifespans** | **> Bioenergy** |
| --- | --- | --- | --- | --- |
| 0 | 0.13 | 0.12 | 0.11 | 0.12 |
| 1 | 2.63 | 2.65 | 2.03 | 1.87 |
| 2 | 5.54 | 5.58 | 4.30 | 3.92 |
| 3 | 5.88 | 5.93 | 4.61 | 4.19 |
| 4 | 6.08 | 6.14 | 4.82 | 4.37 |
| 5 | 6.22 | 6.28 | 4.97 | 4.51 |
| 6 | 6.33 | 6.40 | 5.11 | 4.63 |
| 7 | 6.43 | 6.51 | 5.22 | 4.73 |
| 8 | 6.53 | 6.61 | 5.31 | 4.83 |
| 9 | 6.64 | 6.72 | 5.39 | 4.93 |
| 10 | 6.72 | 6.81 | 5.45 | 5.01 |
| 20 | 7.05 | 7.09 | 6.02 | 5.43 |
| 30 | 7.48 | 7.47 | 6.42 | 5.91 |
| 40 | 7.94 | 7.91 | 6.91 | 6.40 |
| 50 | 8.36 | 8.30 | 7.39 | 6.84 |
| 60 | 8.70 | 8.62 | 7.80 | 7.19 |
| 70 | 8.97 | 8.87 | 8.14 | 7.45 |
| 80 | 9.18 | 9.07 | 8.41 | 7.67 |
| 90 | 9.38 | 9.26 | 8.63 | 7.86 |
| 100 | 9.57 | 9.44 | 8.84 | 8.05 |
| 110 | 9.75 | 9.61 | 9.03 | 8.23 |
| 120 | 9.93 | 9.78 | 9.22 | 8.41 |

CNS = chip-n-saw; reference inputs for the scenarios were loblolly and shortleaf pine timber harvested in the southern US in 2020.

**Table S44.** Biogenic CO_2_ emissions from wood products (excluding landfills) from three wood usage scenarios and a business as usual (BAU) scenario (MtCO_2_e)

| **Years Since Harvest** | **BAU** | **> CNS** | **> Lifespans** | **> Bioenergy** |
| --- | --- | --- | --- | --- |
| 0 | -36.24 | -36.85 | -36.30 | -58.89 |
| 1 | -6.83 | -6.88 | -5.87 | -4.98 |
| 2 | -9.49 | -9.57 | -8.54 | -7.15 |
| 3 | -4.06 | -4.10 | -3.61 | -3.32 |
| 4 | -3.15 | -3.16 | -2.66 | -2.48 |
| 5 | -2.42 | -2.41 | -1.80 | -1.86 |
| 6 | -2.51 | -2.50 | -1.60 | -1.91 |
| 7 | -3.43 | -3.44 | -1.41 | -2.52 |
| 8 | -5.74 | -5.78 | -1.34 | -4.06 |
| 9 | -7.61 | -7.67 | -1.32 | -5.33 |
| 10 | -6.63 | -6.69 | -1.34 | -4.65 |
| 20 | -0.13 | -0.12 | -0.37 | -0.13 |
| 30 | -0.16 | -0.15 | -0.39 | -0.16 |
| 40 | -0.16 | -0.15 | -0.42 | -0.16 |
| 50 | -0.15 | -0.14 | -0.39 | -0.15 |
| 60 | -0.13 | -0.12 | -0.34 | -0.13 |
| 70 | -0.12 | -0.11 | -0.28 | -0.12 |
| 80 | -0.11 | -0.10 | -0.24 | -0.10 |
| 90 | -0.10 | -0.09 | -0.20 | -0.10 |
| 100 | -0.09 | -0.08 | -0.19 | -0.09 |
| 110 | -0.08 | -0.07 | -0.17 | -0.08 |
| 120 | -0.07 | -0.06 | -0.16 | -0.07 |

CNS = chip-n-saw; reference inputs for the scenarios were loblolly and shortleaf pine timber harvested in the southern US in 2020.

**Table S45.** Biogenic CO_2_ emission pulses from landfills from three wood usage scenarios and a business as usual (BAU) scenario (MtCO_2_e)

| **Years Since Harvest** | **BAU** | **> CNS** | **> Lifespans** | **> Bioenergy** |
| --- | --- | --- | --- | --- |
| 0 | 0.00 | 0.00 | 0.00 | 0.00 |
| 1 | 0.00 | 0.00 | 0.00 | 0.00 |
| 2 | -0.09 | -0.09 | -0.07 | -0.06 |
| 3 | -0.19 | -0.19 | -0.14 | -0.13 |
| 4 | -0.19 | -0.19 | -0.14 | -0.13 |
| 5 | -0.18 | -0.18 | -0.14 | -0.13 |
| 6 | -0.18 | -0.18 | -0.14 | -0.12 |
| 7 | -0.17 | -0.17 | -0.13 | -0.12 |
| 8 | -0.16 | -0.16 | -0.13 | -0.11 |
| 9 | -0.16 | -0.16 | -0.12 | -0.11 |
| 10 | -0.15 | -0.15 | -0.12 | -0.11 |
| 20 | -0.10 | -0.10 | -0.09 | -0.07 |
| 30 | -0.06 | -0.06 | -0.05 | -0.04 |
| 40 | -0.04 | -0.04 | -0.04 | -0.03 |
| 50 | -0.03 | -0.03 | -0.02 | -0.02 |
| 60 | -0.02 | -0.02 | -0.02 | -0.01 |
| 70 | -0.01 | -0.01 | -0.01 | -0.01 |
| 80 | -0.01 | -0.01 | -0.01 | -0.01 |
| 90 | -0.01 | -0.01 | -0.01 | 0.00 |
| 100 | 0.00 | 0.00 | 0.00 | 0.00 |
| 110 | 0.00 | 0.00 | 0.00 | 0.00 |
| 120 | 0.00 | 0.00 | 0.00 | 0.00 |

CNS = chip-n-saw; reference inputs for the scenarios were loblolly and shortleaf pine timber harvested in the southern US in 2020.

**Table S46.** Biogenic CH_4_ emission pulses from landfills from three wood usage scenarios and a business as usual (BAU) scenario (MtCO_2_e)

| **Years Since Harvest** | **BAU** | **> CNS** | **> Lifespans** | **> Bioenergy** |
| --- | --- | --- | --- | --- |
| 0 | 0.00 | 0.00 | 0.00 | 0.00 |
| 1 | 0.00 | 0.00 | 0.00 | 0.00 |
| 2 | -0.18 | -0.18 | -0.14 | -0.12 |
| 3 | -0.38 | -0.38 | -0.29 | -0.26 |
| 4 | -0.38 | -0.38 | -0.29 | -0.26 |
| 5 | -0.37 | -0.37 | -0.29 | -0.25 |
| 6 | -0.36 | -0.36 | -0.28 | -0.25 |
| 7 | -0.34 | -0.35 | -0.27 | -0.24 |
| 8 | -0.33 | -0.33 | -0.26 | -0.23 |
| 9 | -0.32 | -0.32 | -0.25 | -0.22 |
| 10 | -0.31 | -0.31 | -0.24 | -0.21 |
| 20 | -0.21 | -0.21 | -0.18 | -0.14 |
| 30 | -0.14 | -0.14 | -0.12 | -0.10 |
| 40 | -0.10 | -0.10 | -0.08 | -0.07 |
| 50 | -0.07 | -0.07 | -0.06 | -0.05 |
| 60 | -0.05 | -0.05 | -0.04 | -0.04 |
| 70 | -0.04 | -0.04 | -0.03 | -0.03 |
| 80 | -0.03 | -0.03 | -0.03 | -0.03 |
| 90 | -0.02 | -0.02 | -0.02 | -0.02 |
| 100 | -0.02 | -0.02 | -0.02 | -0.02 |
| 110 | -0.02 | -0.02 | -0.02 | -0.02 |
| 120 | -0.02 | -0.01 | -0.01 | -0.01 |

CNS = chip-n-saw; reference inputs for the scenarios were loblolly and shortleaf pine timber harvested in the southern US in 2020.

**Table S47.** Fossil fuel emission pulses from procurement, transportation, and production from three wood usage scenarios and a business as usual (BAU) scenario (MtCO_2_e)

| **Years Since Harvest** | **BAU** | **> CNS** | **> Lifespans** | **> Bioenergy** |
| --- | --- | --- | --- | --- |
| 0 | -43.33 | -43.66 | -43.33 | -34.09 |

Reference inputs for the scenarios were loblolly and shortleaf pine timber harvested in the southern US in 2020.

**Table S48.** Total (biogenic + fossil fuel) emission pulses from three wood usage scenarios and a business as usual (BAU) scenario (MtCO_2_e)

| **Years Since Harvest** | **BAU** | **> CNS** | **> Lifespans** | **> Bioenergy** |
| --- | --- | --- | --- | --- |
| 0 | -79.57 | -80.51 | -79.62 | -92.98 |
| 1 | -6.83 | -6.88 | -5.88 | -4.98 |
| 2 | -9.76 | -9.84 | -8.74 | -7.34 |
| 3 | -4.62 | -4.67 | -4.04 | -3.70 |
| 4 | -3.71 | -3.73 | -3.10 | -2.86 |
| 5 | -2.97 | -2.96 | -2.23 | -2.24 |
| 6 | -3.04 | -3.03 | -2.01 | -2.28 |
| 7 | -3.95 | -3.96 | -1.81 | -2.87 |
| 8 | -6.23 | -6.28 | -1.73 | -4.40 |
| 9 | -8.08 | -8.15 | -1.70 | -5.66 |
| 10 | -7.10 | -7.16 | -1.70 | -4.97 |
| 20 | -0.44 | -0.43 | -0.63 | -0.34 |
| 30 | -0.37 | -0.36 | -0.56 | -0.30 |
| 40 | -0.30 | -0.29 | -0.53 | -0.26 |
| 50 | -0.25 | -0.23 | -0.47 | -0.22 |
| 60 | -0.20 | -0.19 | -0.40 | -0.19 |
| 70 | -0.17 | -0.16 | -0.33 | -0.16 |
| 80 | -0.15 | -0.14 | -0.27 | -0.14 |
| 90 | -0.13 | -0.12 | -0.23 | -0.12 |
| 100 | -0.11 | -0.11 | -0.21 | -0.11 |
| 110 | -0.10 | -0.09 | -0.19 | -0.10 |
| 120 | -0.09 | -0.08 | -0.18 | -0.08 |

Reference inputs for the scenarios were loblolly and shortleaf pine timber harvested in the southern US in 2020.


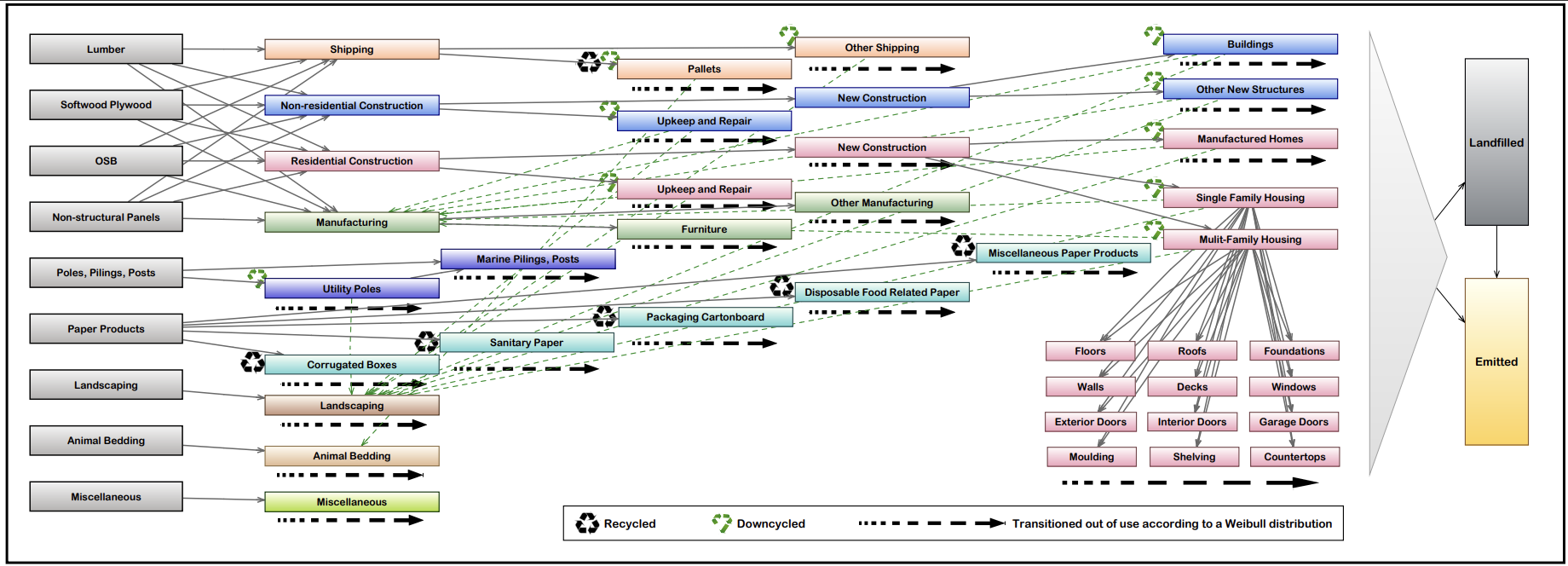


**Figure S1.** Flow of carbon from primary products to eventual emission. Shows the transition to secondary products, recycling, downcycling, landfilling, and emission.

**References**

1. Anderson RG, McKeever DB. Wood used in new Residential Construction in the United States 1988. 1988;

2. Brandeis C, Taylor M, Abt KL, Alderman D, Buehlmann U. Status and Trends for the U.S. Forest Products Sector: A Technical Document Supporting the Forest Service 2020 RPA Assessment [Internet]. Asheville, NC: U.S. Department of Agriculture, Forest Service, Southern Research Station; 2021 Jan [cited 2022 Nov 7] p. SRS-GTR-258. Report No.: SRS-GTR-258. Available from: https://www.fs.usda.gov/treesearch/pubs/61862

3. McKeever DB, Elling J. Wood Products and Other Building Materials Used in New Residential Construction in the United States, with comparison to previous studies 2012. APA - The Engineered Wood Assoc. 2015;75 p.

4. Fastmarkets RISI. Mill Asset Database [Internet]. 2023. Available from: https://www.risiinfo.com/millassets/mill-groups/capacity.html?sid=934424

5. US EPA, Zimmer T, Weitz K, Padhye A, Sifleet S. Wood Waste Inventory: Final Report. 2018 Jul. Report No.: EPA/600/R-18/262.

6. Ianchenko A, Simonen K, Barnes C. Residential Building Lifespan and Community Turnover. J Archit Eng. 2020 Sep;26(3):04020026.

7. Seiders DD, Ahluwalia G, Melman S, Quint R, Chaluvadi A, Liang M, et al. National Association of Home Builders/ Bank of America Home Equity Study of Life Expectancy of Home Components. Jackson J, editor. 2007 Feb;

8. InterNACHI. Standard Estimated Life Expectancy Chart for Homes [Internet]. International Association of Certified Home Inspectors; n.d. Available from: https://www.nachi.org/life-expectancy.htm

9. Skog KE. Sequestration of carbon in harvested wood products for the United States. Forest Products Journal; 2008.

10. Arehart JH, Pomponi F, D’Amico B, Srubar WV. Structural material demand and associated embodied carbon emissions of the United States building stock: 2020–2100. Resources, Conservation and Recycling. 2022 Nov;186:106583.

11. Janjua SY, Sarker PK, Biswas WK. Sustainability implications of service life on residential buildings – An application of life cycle sustainability assessment framework. Environmental and Sustainability Indicators. 2021 Jun;10:100109.

12. O’Connor J. Survey on actual service lives for North American buildings. 2004 Oct;

13. Bigelow J, Lebow S, Clausen CA, Greimann L, Wipf TJ. Preservation Treatment for Wood Bridge Application. Transportation Research Record. 2009 Jan;2108(1):77–85.

14. Gibson JR. Built to Last: Challenges and Opportunities for Climate-Smart Infrastructure in California [Internet]. Union of Concerned Scientists; 2017 Nov. Available from: www.ucsusa.org/climate-smart-infrastructure

15. Smith ST. Economic Evaluation of Treated Wood and Galvanized Steel Guardrail Posts [Internet]. Washington State Department of Transportation (WSDOT); 2013 May. Available from: https://preservedwood.org/portals/0/documents/EconEval_Guardrail.pdf

16. Bilbao A. Environmental Impact Analysis of Alternative Pallet Management Systems. Rochester Institute of Technology; 2011.

17. Bolin CA, Smith ST. Life cycle assessment of pentachlorophenol-treated wooden utility poles with comparisons to steel and concrete utility poles. Renewable and Sustainable Energy Reviews. 2011 Jun;15(5):2475–86.

18. Bolin CA, Smith ST. Life Cycle Assessment of CCA-Treated Wood Marine Piles in the U.S. with Comparisons to Concrete, Galvanized Steel, and Plastic Marine Piles. J of Marine Env Eng. 2012;9:239–60.

19. Bell N, Sullivan D, Cook T. Mulching Woody Ornamentals with Organic Materials [Internet]. OSU Extension Service; 2009. Available from: https://catalog.extension.oregonstate.edu/ec1629

20. EPA. Advancing Sustainable Materials Management: 2018 Fact Sheet. 2020 Dec;

21. Palma VM, Pettenella DM, Zanetti M, Animon I, Tracogna A. Estimating wooden kitchen furniture’s contribution to climate change mitigation. int forest rev. 2017 Jun 1;19(2):224–33.

22. Albastroiu Nastase I, Negrutiu C, Felea M, Acatrinei C, Cepoi A, Istrate A. Toward a Circular Economy in the Toy Industry: The Business Model of a Romanian Company. Sustainability. 2021 Dec 21;14(1):22.

23. Hunt B. Depreciation of Musical Instruments [Internet]. Institute for Music Leadership. 2006 [cited 2023 Jun 1]. Available from: https://iml.esm.rochester.edu/polyphonic-archive/article/depreciation-of-musical-instruments/#:~:text=Musical%20instruments%20are%20considered%20to,using%20the%20half%2Dyear%20convention.

24. Peacock J. Do Caskets Decompose in the Ground? (+ Materials Compared) [Internet]. 2023 [cited 2023 Jun 1]. Available from: https://afteryourtime.com/do-caskets-decompose/#:~:text=to%20four%20years.-,How%20Long%20Do%20Wooden%20Caskets%20Last%3F,last%20less%20than%20ten%20years.

25. Shantz J. How Long Does a Kayak Last? 5 Awesome Materials Examined [Internet]. Boating Guide. 2023 [cited 2023 Jun 1]. Available from: https://boating.guide/how-long-does-a-kayak-last/#gsc.tab=0

26. Araman PA, Bush RJ, Hammett AL, Hager EB. WOOD PALLETS AND LANDFILLS – STATUS AND OPPORTUNITIES FOR ECONOMIC RECOVERY AND RECYCLING. USFS. 1998;

27. Gerber N, Horvath L, Araman P, Gething B. Investigation of new and recovered wood shipping platforms in the United States. BioRes. 2020 Mar 6;15(2):2818–38.

28. Shiner Z, Horvath L, Araman P, Gething B. An investigation of wood pallets landfilled and recovered at US municipal solid waste facilities. BioRes. 2021 Jan 10;16(1):1496–522.

29. EPA. Advancing Sustainable Materials Management: 2018 Tables and Figures [Internet]. 2020. (Assessing Trends in Materials Generation and Management in the United States). Available from: https://www.epa.gov/facts-and-figures-about-materials-waste-and-recycling/advancing-sustainable-materials-management

30. Fourcassier S, Douziech M, Pérez-López P, Schiebinger L. Menstrual products: A comparable Life Cycle Assessment. Cleaner Environmental Systems. 2022 Dec;7:100096.

31. Milota M, Puettmann M. Life cycle assessment for the production of southeastern softwood lumber. 2019. (CORRIM Final Report).

32. Puettmann M, Kaestner D, Taylor A. Life Cycle Assessment for the Production of Southeast Softwood Plywood. 2020 Mar. (CORRIM Final Report).

33. Puettmann M, Kaestner D, Taylor A. Life Cycle Assessment for the Production of Oriented Strandboard Production. 2020 Mar. (CORRIM Final Report).

34. Puettmann M, Salazar J. Cradle to Gate Life Cycle Assessment of North American Particleboard Production. 2018 Oct. (CORRIM Final Report).

35. Puettmann M, Salazar J. Cradle to Gate Life Cycle Assessment of North American Medium Density Fiberboard Production. 2019 Mar. (CORRIM Final Report).

36. Bergman RD, Alanya-Rosenbaum S. Cradle-to-Gate Life Cycle Assessment of Laminated Veneer Lumber (LVL) Produced in the Southeast Region of the United States. 2017 Apr. (CORRIM Final Report).

37. CORRIM. Cradle to Gate Life Cycle Assessment of North American Laminated Strand Lumber Production. 2015 Nov. (CORRIM Final Report).

38. Puettmann M, Bergman R, Oneil E. Cradle-To-Gate Life Cycle Assessment of North American Hardboard and Engineered Wood Siding and Trim Production. Seattle, WA: University of Washington; 2016 Jul. (CORRIM Final Report).

39. Puettmann M, Oneil E, Johnson L. Cradle to Gate Life Cycle Assessment of Glue-Laminated Timbers Production from the Southeast. 2013 Jan. (CORRIM Final Report).

40. Puettmann M, Bergman R, Oneil E. Cradle to Gate Life Cycle Assessment of North American Cellulosic Fiberboard Production. 2016 Jan. (CORRIM Final Report).

41. Puettmann M, Sinha A, Ganguly I. Life Cycle Assessment of Cross Laminated Timbers Produced in Oregon. 2018 Feb. (CORRIM Final Report).

42. Tomberlin KE, Venditti R, Yao Y. Life cycle carbon footprint analysis of pulp and paper grades in the United States using production-line-based data and integration. BioRes. 2020 Apr 7;15(2):3899–914.

43. Morrison B, Golden JS. Southeastern United States wood pellets as a global energy resource: a cradle-to-gate life cycle assessment derived from empirical data. International Journal of Sustainable Energy. 2018 Feb 7;37(2):134–46.

44. Micales JA, Skog KE. The decomposition of forest products in landfills. International Biodeterioration & Biodegradation. 1997 Jan;39(2–3):145–58.

45. O’Dwyer J, Walshe D, Byrne KA. Wood waste decomposition in landfills: An assessment of current knowledge and implications for emissions reporting. Waste Management. 2018 Mar;73:181–8.

46. Pingoud K, Wagner F. Methane Emissions from Landfills and Carbon Dynamics of Harvested Wood Products: The First-Order Decay Revisited. Mitig Adapt Strat Glob Change. 2006 Sep;11(5–6):961–78.

47. EPA. Inventory of U.S. Greenhouse Gas Emissions and Sinks: 1990-2021. U.S. Environmental Protection Agency, EPA; 2023. Report No.: EPA 430-R-23-002.

48. Smith JE, Heath LS, Skog KE, Birdsey RA. Methods for calculating forest ecosystem and harvested carbon with standard estimates for forest types of the United States [Internet]. Newtown Square, PA: U.S. Department of Agriculture, Forest Service, Northeastern Research Station; 2006 p. NE-GTR-343. Report No.: NE-GTR-343. Available from: https://www.fs.usda.gov/treesearch/pubs/22954

49. Stockmann KD, Anderson NM, Skog KE, Healey SP, Loeffler DR, Jones G, et al. Estimates of carbon stored in harvested wood products from the United States forest service northern region, 1906-2010. Carbon Balance Manage. 2012 Dec;7(1):1.

50. USFS. Timber Products Output [Internet]. 2020. Available from: https://www.fia.fs.usda.gov/program-features/tpo/

51. Milota MR. GATE-TO-GATE LIFE-CYCLE INVENTORY OF SOFTWOOD LUMBER PRODUCTION. WOOD AND FIBER SCIENCE. 2005;37:11.

52. Spelter H. Profile 2007: Softwood Sawmills in the United States and Canada. :69.

53. Steele PH. Factors determining lumber recovery in sawmilling [Internet]. Madison, WI: U.S. Department of Agriculture, Forest Service, Forest Products Laboratory; 1984 [cited 2022 Nov 7] p. FPL-GTR-39. Report No.: FPL-GTR-39. Available from: https://www.fs.usda.gov/treesearch/pubs/8907

54. Carrano AL, Thorn BK, Woltag H. Characterizing the Carbon Footprint of Wood Pallet Logistics. For Prod J. 2014;64(7/8).

55. AF&PA. How Does AF&PA Calculate Paper and Cardboard Recycling Rates? [Internet]. 2022 [cited 2023 Jun 1]. Available from: https://www.afandpa.org/news/2022/how-does-afpa-calculate-paper-and-cardboard-recycling-rates

56. Schmidt JH, Holm P, Merrild A, Christensen P. Life cycle assessment of the waste hierarchy – A Danish case study on waste paper. Waste Management. 2007 Jan;27(11):1519–30.
